# Plant phytochrome interactions decode light and temperature signals

**DOI:** 10.1093/plcell/koae249

**Published:** 2024-09-11

**Authors:** Chengwei Yi, Uwe Gerken, Kun Tang, Michael Philipp, Matias D Zurbriggen, Jürgen Köhler, Andreas Möglich

**Affiliations:** Department of Biochemistry, University of Bayreuth, 95447 Bayreuth, Germany; Lehrstuhl für Spektroskopie weicher Materie, Universität Bayreuth, 95447 Bayreuth, Germany; Institute of Synthetic Biology, Heinrich Heine University Düsseldorf, 40225 Düsseldorf, Germany; Lehrstuhl für Spektroskopie weicher Materie, Universität Bayreuth, 95447 Bayreuth, Germany; Institute of Synthetic Biology, Heinrich Heine University Düsseldorf, 40225 Düsseldorf, Germany; CEPLAS – Cluster of Excellence on Plant Sciences, Heinrich Heine University Düsseldorf, 40225 Düsseldorf, Germany; Lehrstuhl für Spektroskopie weicher Materie, Universität Bayreuth, 95447 Bayreuth, Germany; Bayerisches Polymer Institut, Universität Bayreuth, 95447 Bayreuth, Germany; Bayreuther Institut für Makromolekülforschung, Universität Bayreuth, 95447 Bayreuth, Germany; Department of Biochemistry, University of Bayreuth, 95447 Bayreuth, Germany; Bayreuth Center for Biochemistry & Molecular Biology, Universität Bayreuth, 95447 Bayreuth, Germany; North-Bavarian NMR Center, Universität Bayreuth, 95447 Bayreuth, Germany

## Abstract

Plant phytochromes perceive red and far-red light to elicit adaptations to the changing environment. Downstream physiological responses revolve around red-light-induced interactions with phytochrome-interacting factors (PIFs). Phytochromes double as thermoreceptors, owing to the pronounced temperature dependence of thermal reversion from the light-adapted Pfr to the dark-adapted Pr state. Here, we assess whether thermoreception may extend to the phytochrome:PIF interactions. While the association between Arabidopsis (*Arabidopsis thaliana*) PHYTOCHROME B (PhyB) and several PHYTOCHROME-INTERACTING FACTOR (PIF) variants moderately accelerates with temperature, the dissociation does more so, thus causing net destabilization of the phytochrome:PIF complex. Markedly different temperature profiles of PIF3 and PIF6 might underlie stratified temperature responses in plants. Accidentally, we identify a photoreception mechanism under strong continuous light, where the extent of phytochrome:PIF complexation decreases with red-light intensity rather than increases. Mathematical modeling rationalizes this attenuation mechanism and ties it to rapid red-light-driven Pr⇄Pfr interconversion and complex dissociation out of Pr. Varying phytochrome abundance, e.g. during diurnal and developmental cycles, and interaction dynamics, e.g. across different PIFs, modify the nature and extent of attenuation, thus permitting light-response profiles more malleable than possible for the phytochrome Pr⇄Pfr interconversion alone. Our data and analyses reveal a photoreception mechanism with implications for plant physiology, optogenetics, and biotechnological applications.

## Teaser

The interplay of plant phytochromes with their interacting factors contributes to thermoreception and underlies an additional mode of photoreception under strong light.

## Introduction

**Figure 1.**
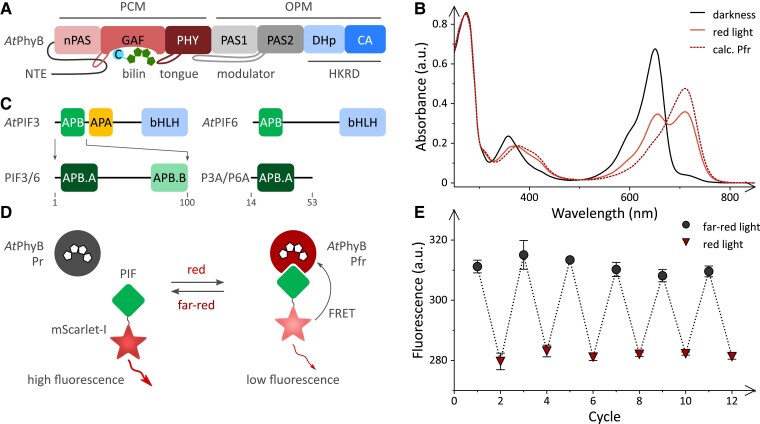
Analysis of light-triggered interactions between Arabidopsis PhyB and its phytochrome-interacting factors (PIF) 3 and 6. **A)** Domain architecture of *At*PhyB comprising an N-terminal extension (NTE), the nPAS-GAF-PHY photosensory core module (PCM), and an output module (OPM) consisting of PAS1, PAS2, and the histidine-kinase-related domains (HKRD) DHp and CA. The bilin within the GAF moiety is indicated, as are the PHY tongue and the PAS2 modulator loop (Li et al. 2022). **B)** Absorbance spectrum of the *At*PhyB PCM in darkness (solid black line). Exposure to red light leads to population of a 0.27:0.73 Pr:Pfr photostationary mixture (solid red line). The calculated spectrum of the pure Pfr state is shown as a dashed brown line. **C)** Domain architecture of Arabidopsis PIF 3 and 6 with C-terminal basic helix–loop–helix (bHLH) DNA-binding entities. The N-terminal segment comprising the APB motif suffices for light-activated binding to *At*PhyB. PIF3 additionally contains an APA motif responsible for interactions with *At*PhyA. The APB motif subdivides into APB.A and APB.B parts, with the former predominantly mediating the *At*PhyB interaction (Golonka et al. 2019). Throughout this work, the variants PIF3 and PIF6 (residues 1 to 100), as well as P3A and P6A (residues 14 to 53), lacking APB.B were studied. **D)** To analyze the *At*PhyB:PIF interaction dynamics, the PIF variants were fused at their C termini to mScarlet-I. This fluorescent protein and the Pfr state of *At*PhyB form a Förster resonance energy transfer (FRET) pair with a characteristic distance *R*_0_ of 58 Å (see Supplementary Fig. S1C). Red light drives the Pr→Pfr conversion of *At*PhyB and prompts its association with PIF variants. The mScarlet-I label is thereby brought into spatial vicinity of *At*PhyB, and its fluorescence reduces because of FRET. Far-red light promotes the Pfr→Pr reversion, thus triggering dissociation of the *At*PhyB:PIF complex and an increase of mScarlet-I fluorescence. **E)** In the presence of 2 *μ*m  *At*PhyB PCM, the fluorescence of 20 nm P6A-mScarlet-I alternated between high and low values upon illumination with far-red (733 nm) and red light (658 nm), respectively (odd and even cycle numbers shown as black circles and brown triangles, respectively). Data in panel **E** represent mean ± SD of 3 independent experiments.

Numerous organisms harness sensory photoreceptors to derive spatial and temporal cues from incident light for the vital adaptation of behavior, development, and physiology (Hegemann 2008; Möglich et al. 2010). Phytochromes (Phy), originally identified as photosensitive pigments in land plants (Butler et al. 1959) and later in bacteria (Hughes et al. 1997; Davis et al. 1999), afford sensation of red and far-red (i.e. near-infrared) light (Rockwell and Lagarias 2010; Burgie and Vierstra 2014; Legris et al. 2019; Rockwell and Lagarias 2020). Phys generally exhibit bipartite architecture with an N-terminal photosensory core module (PCM, also referred to as photosensory module [PSM]) and a C-terminal output, or, effector, module (OPM). The PCM comprises the structurally homologous PAS (Per-ARNT-Sim (Möglich et al. 2009)), GAF (cGMP-specific phosphodiesterases, adenylyl cyclases, and FhlA (Aravind and Ponting 1997)), and PHY domains (Essen et al. 2008; Yang et al. 2008) (Fig. 1A). Covalently bound as prosthetic groups and embedded in the GAF domain, linear tetrapyrrole (i.e. bilin) chromophores allow the absorption of red and far-red light. In darkness, conventional Phys adopt their red-absorbing Pr state which is characterized by a *Z* configuration of the C15 = C16 double bond within the bilin cofactor. Red light drives the photoconversion to the Pfr state with a 15*E*-configured bilin. Reversion to the dark-adapted Pr state occurs thermally in the dark-recovery reaction or can be driven by far-red light. Bilin isomerization couples to the PCM and subsequently to the OPM via the so-called PHY tongue, a polypeptide stretch extending from the PHY domain and contacting the preceding GAF domain and the enclosed chromophore (Takala et al. 2014). The relative uniformity of the PCMs across bacteria and plants (Essen et al. 2008; Yang et al. 2008; Burgie et al. 2014; Nagano et al. 2020) contrasts with the structural and functional diversity of OPMs evidenced in different Phys. Whereas the PCMs of the architecturally simpler bacterial Phys commonly couple to enzymatic output functionalities, the OPMs of plant Phys are likely devoid of catalytic activity (Li et al. 2022) although individual studies assigned them serine/threonine kinase activity early on (Yeh and Lagarias 1998) (Fig. 1A). Rather, plant Phys elicit light-dependent downstream adaptations via protein:protein interactions, nuclear import and export, and proteolytic degradation (Pham et al. 2018; Legris et al. 2019). Transcending their origins, plant Phys and the light-inducible interactions they enter can be also leveraged for optogenetics in heterologous organisms (Shimizu-Sato et al. 2002; Deisseroth et al. 2006; Levskaya et al. 2009; Müller et al. 2013a, 2013b; Golonka et al. 2019; Tang et al. 2021), i.e. for controlling by light cellular physiology and parameters.

Of the 5 phytochromes present in Arabidopsis (*Arabidopsis thaliana*), designated PhyA through PhyE, PhyA and PhyB are best-studied and of prime physiological importance (Li et al. 2011; Legris et al. 2019). PhyA elicits responses to low light intensities, known as the very low fluence response (VLFR), or to higher intensities when the ratio of red relative to far-red light is low, a reaction known as the far-red high-irradiance response (FR-HIR). By contrast, PhyB accounts for the so-called low fluence response (LFR) at higher ratios of red to far-red light and across a wide range of fluence rates. Additionally, PhyB carries the HIR response at high ratios of red to far-red light. Equally recent and groundbreaking cryo-electron microscopy data revealed at full length the 3-dimensional structures of *A. thaliana* PhyB (*At*PhyB) (Li et al. 2022) and PhyA (*At*PhyA) (Burgie et al. 2023). Despite distinct physiological responses and fluence-rate sensitivities, the 2 Phys adopt overall similar homodimeric structures. Unexpectedly, the PCMs are not arranged in parallel, as seen for bacterial Phys (Essen et al. 2008; Yang et al. 2008; Gourinchas et al. 2017) and as suggested by isolated PCM structures of plant Phys (Burgie et al. 2014; Nagano et al. 2020), but in head-to-tail manner. Within the dimeric receptor, the PCMs provide a platform onto which the C-terminal histidine-kinase-related domains (HKRD), comprising DHp and CA entities, assemble in head-to-head orientation. Intriguingly, many light-dependent Phy responses can occur in the absence of the HKRDs and the PAS1 and PAS2 domains directly preceding them. Specifically, in both *At*PhyA and *At*PhyB, the isolated PCMs and an N-terminal extension (NTE) alone can mediate light-dependent interactions with phytochrome-interacting factors (PIFs) (Shimizu-Sato et al. 2002; Levskaya et al. 2009; Pham et al. 2018). That notwithstanding, the C-terminal OPM is centrally engaged in light-dependent interactions with PIFs as well (Ni et al. 1998; Ni et al. 1999). Many downstream effects and physiological light responses strictly hinge on the OPM, e.g. the light-induced, phytochrome-mediated degradation of PIFs (Park et al. 2012, 2018; Qiu et al. 2019; Yoo et al. 2021).

The recent structural and functional data (Li et al. 2022; Burgie et al. 2023) assign to the HKRD an important role as a dimerization nexus. Beyond receptor dimerization, interactions between the HKRD and the PCM platform apparently determine the lifetime of the Pfr state after red-light activation. Tantalizingly, the HKRDs seem to substantially speed up the Pfr→Pr dark recovery, with the magnitude of this effect in *At*PhyA and *At*PhyB being potentially connected with the extent of structural contacts between the PCM platform and the HKRDs. This has important consequences because the Pfr→Pr recovery reaction of Phys is intricately linked to their action as thermoreceptors, a role recently emerging for PhyB (Jung et al. 2016; Legris et al. 2016; Kerbler and Wigge 2023) and possibly other Phys. Notably, the kinetics of recovery govern how long the Pfr state persists following activation by red light. The recovery process of *At*PhyB is associated with a sizeable activation energy of almost 100 kJ mol^−1^, and over the temperature range from 4 to 27 °C, the dwell time in the Pfr state diminishes from around 12 h to less than 1 h (Jung et al. 2016). Importantly, the recovery not only plays out at night but also during the day while the plant is still exposed to (red) light. Under constant illumination, a photostationary equilibrium arises between the light-driven Pr⇄Pfr interconversion and the temperature-dependent Pfr→Pr recovery. PhyB thereby crucially contributes to the sensing and integration of light and temperature cues.

While the importance of the recovery reaction to thermosensing is now well established, it is less clear whether and to what extent downstream Phy interactions, prominently with their PIF partners, are also relevant. That is not least because quantitative data on these interactions, let alone at different temperatures, are sparse (Golonka et al. 2019; Golonka et al. 2020). To address this dearth and to assess whether Phy:PIF interactions could principally contribute to thermosensing and the interplay with light signals, we studied the interaction dynamics between *At*PhyB and PIFs by fluorescence spectroscopy at different light fluence rates and temperatures. Whereas the association kinetics moderately sped up between 15 and 30 °C, the velocity of the dissociation reaction rose more strongly, and consequently the PIF affinity of the Pfr state dropped. While the association rate constants increased faster with temperature for PIF3 than for PIF6 variants, the opposite proved true for the dissociation reaction. As a corollary, the *At*PhyB interaction with PIF6 exhibited a more pronounced temperature dependence than that with PIF3. In the high-fluence regime, the *At*PhyB:PIF interaction unexpectedly weakened with red-light intensity rather than strengthened. Mathematical modeling rationalizes these findings and ties them to rapid red-light-driven and bidirectional interconversion between the Pr and Pfr states. The underlying attenuation mechanism holds important caveats for the use of Phys in optogenetics and likely also impacts the sensing and integration of light and temperature signals in plants. Plant Phys may thus decode varying red-light fluences and temperatures into physiological responses by an additional, as-of-yet unappreciated and hence unexplored molecular mechanism.

## Results

### Fluorescence-based monitoring of *At*PhyB interactions

To gain additional insight into the interplay of photo- and thermoreception by plant phytochromes, we set out to investigate the interaction dynamics between *At*PhyB and several PIF variants at different light fluence rates and temperatures. As amply demonstrated, e.g. in Shimizu-Sato et al. (2002), Levskaya et al. (2009), and Müller et al. (2013a, 2013b), the N-terminal portion of *At*PhyB lacking PAS1, PAS2, and the HKRDs suffices for red-light-dependent PIF interactions. Accordingly, we at present studied N-terminal *At*PhyB fragments, encompassing the NTE and the PCM, and ligated with phycocyanobilin as chromophore (Golonka et al. 2019; Golonka et al. 2020) (Fig. 1A). For simplicity, we refer to this protein as *At*PhyB in the following. In darkness, *At*PhyB assumes its Pr state with a Q-band absorbance maximum around 650 nm (Fig. 1B). Exposure to a red-light-emitting diode (LED, [658 ± 10] nm) (Supplementary Fig. S1A) gave rise to a Pr:Pfr mixture at a 0.27:0.73 ratio, as determined by absorbance measurements (Butler et al. 1964). The inability to fully populate the Pfr state under red light is well documented and stems from the spectral overlap of Pr and Pfr absorbance (Butler et al. 1959; Butler et al. 1964; Rockwell and Lagarias 2010). Given the bathochromic shift of the Pfr state with a Q-band maximum at 710 nm, all wavelengths within the UV/vis spectral region which the Pr state substantially absorbs excite the Pfr state as well, if to often lower extent. Notably, increasing the light intensity accelerates the rate of Pr⇄Pfr interconversion but does not affect the ratio of these states at photostationary equilibrium. Illumination with a far-red LED emitting at (733 ± 12) nm (Supplementary Fig. S1A) drove *At*PhyB fully back to its Pr dark-adapted state. If kept in darkness, the Pfr state thermally reverts to the more stable Pr state with a time constant > 200 h at 15 °C (Supplementary Fig. S1B). At 30 °C, the recovery reaction sped up 10-fold. Notably, inclusion of the OPM accelerates this process, and the full-length receptor recovers to its Pr state considerably faster (Li et al. 2022).

Analogous to the Phys, the PIF proteins undergo light-regulated interactions in the absence of their C-terminal halves. Specifically, the first hundred amino acids of the Arabidopsis PIFs 1 to 8 contain the so-called APB motif which is responsible for interactions with *At*PhyB (Fig. 1C); by contrast, the APA motif that mediates binding to *At*PhyA is only present in *At*PIF1 and *At*PIF3 (Pham et al. 2018). The APB motif subdivides into the APB.A and APB.B parts, where APB.A is chiefly required for *At*PhyB binding, and APB.B modulates the strength and light dependence of the interaction (Golonka et al. 2019). Provided APB.A is retained, *At*PIF 3 and 6, and possibly other PIFs, can be stripped down to around 25 amino acids that still exhibit light-activated binding to *At*PhyB, if at somewhat reduced efficiency. In the present study, we focused on *At*PIF3 and *At*PIF6, given their importance for plant photoadaptation and optogenetics (Shimizu-Sato et al. 2002; Levskaya et al. 2009; Müller et al. 2013a, 2013b; Pham et al. 2018). For each of the 2 PIFs, we generated 2 versions that comprised, respectively, the first 100 amino acids, referred to as PIF3 and PIF6 in the following, or merely the APB.A portion, referred to as P3A (residues 14-55) and P6A (residues 14-53) (Fig. 1C).

To allow the dynamic monitoring of the interaction with *At*PhyB, we covalently linked the PIF variants at their C termini to the fluorescent protein mScarlet-I (Bindels et al. 2017), which has excitation and emission spectra similar to the homotetrameric *Ds*Red (Strack et al. 2008) we used previously (Golonka et al. 2020) but assumes monomeric state (Fig. 1D). Due to the spectral overlap between the respective emission and absorbance spectra, mScarlet-I and the *At*PhyB Pfr state form a Förster resonance energy transfer (FRET) pair with a characteristic distance *R*_0_ of 58 Å (Supplementary Fig. S1C). The interaction between *At*PhyB and the PIF proteins can hence be tracked by steady-state and time-resolved intensity measurements as a decrease of the mScarlet-I fluorescence. In the presence of a 2 *μ*m excess of *At*PhyB, the fluorescence of P6A-mScarlet-I diminished by 14% under red light (658 nm) compared to far-red light (Fig. 1E). These fluorescence intensity changes reflect the *At*PhyB:PIF interaction and were fully reversible over repeated cycles of red and far-red illumination. When titrating with increasing *At*PhyB concentrations under red light at 15 °C, the P6A-mScarlet-I fluorescence monotonically decreased (Supplementary Fig. S2). Evaluation according to a single-site binding isotherm yielded a dissociation constant of (180 ± 110) nm. Correcting for the partial population of the Pfr state under red light (Fig. 1B), a dissociation constant of (130 ± 80) nm results. This value is a tad lower than but in overall good agreement with our earlier affinity determination using a yellow-fluorescent protein, somewhat higher temperature, and anisotropy measurements (Golonka et al. 2019).

### Light fluence rate modulates kinetics and extent of the *At*PhyB:PIF interaction

**Figure 2.**
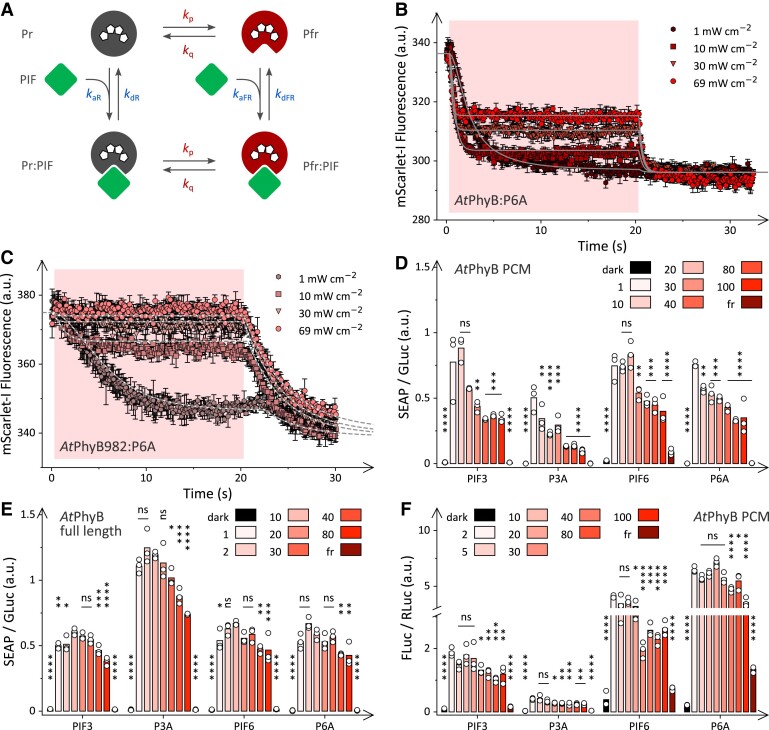
Light-dependent *At*PhyB:PIF interaction dynamics. **A)** Schematic of the *At*PhyB:PIF interaction dynamics. Red light drives both the forward Pr→Pfr and the reverse Pfr→Pr conversion of *At*PhyB with rate constants *k*_p_ and *k*_q_, respectively. In both the Pr and Pfr states, *At*PhyB may bind the PIF protein with kinetics determined by the bimolecular rate constants *k*_aR_ and *k*_aFR_. Once formed, the *At*PhyB:PIF complex may dissociate in unimolecular reactions with rate constants *k*_dR_ and *k*_dFR_ in the Pr and Pfr states, respectively. **B)** 20 nm P6A-mScarlet-I and 1,000 nm  *At*PhyB were incubated at 15 °C, and the mScarlet-I fluorescence was measured at (565 ± 20) nm excitation and (600 ± 20) nm emission. During the initial 20 s of the experiment (pink shaded area), the samples were illuminated with 658-nm light at different intensities (from bottom to top 1 mW cm^−2^ [dark brown hexagons], 10 mW cm^−2^ [medium brown squares], 30 mW cm^−2^ [light brown triangles], and 69 mW cm^−2^ [red circles]). The solid lines represent a global fit of the data to the numeric solution of the reaction scheme in panel **A**. **C)** As in panel **B** but for 20 nm P6A-mScarlet-I mixed with 2,000 nm  *At*PhyB (1-982). The global fit is shown as the set of dashed lines. Data in panels **B** and **C** represent mean ± SD of 3 independent experiments. **D)** CHO-K1 cells were transfected with a construct encoding the *At*PhyB PCM linked to a VP16 transactivation domain and a given PIF variant connected to the erythromycin repressor DNA-binding domain (see Supplementary Fig. S6A and B). Upon red-light-induced heterodimerization, the 2 components drove the expression of a secreted alkaline phosphatase (SEAP) reporter. The reporter signal was measured for systems based on PIF3, P3A, PIF6, and P6A in darkness, at 660-nm light intensities ranging from 1 to 100 *μ*E s^−1^ m^−2^ as listed in the figure, or under 20 *μ*E s^−1^ m^−2^ 740-nm light. Values obtained at the individual light conditions were normalized to a constitutively expressed *Gaussia* luciferase (GLuc) and compared to the SEAP signal under 1 *μ*E s^−1^ m^−2^ 660-nm light. Asterisks denote 1-way ANOVA significance levels of *P < 0.05, **P < 0.01, ***P < 0.001, and ****P < 0.0005. Data points represent biologically independent triplicate measurements. **E)** As in panel **B** but with the VP16 transactivation domain linked to full-length *At*PhyB rather than the *At*PhyB PCM. Results were compared to those at a red-light intensity of 10 *μ*E s^−1^ m^−2^, with significance levels calculated as in panel **D**. **F)** Arabidopsis protoplasts were transfected with a construct encoding the *At*PhyB PCM linked to a VP16 transactivation domain and a given PIF variant connected to the erythromycin DNA-binding domain (see Supplementary Fig. S6A and C). Together, the 2 components controlled the expression of a firefly luciferase reporter (FLuc). As in panel **C**, the cells were incubated under different illumination conditions, and the FLuc reporter signal was measured and normalized to a constitutively expressed *Renilla* luciferase (RLuc). Results were compared to those at a red-light intensity of 2 *μ*E s^−1^ m^−2^, with significance levels calculated as in panel **D**. Bars in panels **D** to **F** represent the mean of 3 biologically independent measurements which are shown as white circles.

Next, we assessed the kinetics of association and dissociation of the *At*PhyB:PIF complex following exposure to red and far-red light, respectively. For such measurements, it is imperative that the *At*PhyB photoconversion (rate constants *k*_p_ for the Pr→Pfr conversion and *k*_q_ for the Pfr→Pr reversion) be fast relative to the subsequent association (rate constant *k*_a_) and dissociation events (rate constant *k*_d_), lest the kinetics that can be resolved become limited (Golonka et al. 2020) (Fig. 2A). We therefore first probed the kinetics of Pr→Pfr conversion under red light by absorbance spectroscopy (Supplementary Fig. S3A). Under constant red light (658 nm) at intensities of 1, 10, 30, and 69 mW cm^−2^, the Pfr state, monitored at 715 nm, built up in single-exponential fashion with rate constants of 0.19, 1.3, 2.5, and 5.2 s^−1^. Apart from absorbance measurements, the Pr:Pfr ratio can also be followed by the weak *At*PhyB fluorescence in its Pr state (Supplementary Fig. S4A). Doing so has the decisive advantage that the same instrumental setup applies as for the subsequent interaction measurements by FRET. When exposed to 658-nm light at intensities of 1, 10, 30, and 69 mW cm^−2^, the Pr fluorescence of *At*PhyB monotonically decreased with rate constants of 0.25, 1.6, 4.2, and 6.2 s^−1^ to reach a constant plateau (Supplementary Fig. S4A). Overall, the velocities of the Pr→Pfr photoconversion determined for the individual light intensities by absorbance and fluorescence are thus in reasonable agreement, considering the somewhat different illumination geometries between the respective spectrophotometers used for the 2 measurements. This suggests that the Pr-state fluorescence accurately reports on the *At*PhyB Pr population. Given the 0.1-s time resolution of the fluorescence measurements, the values determined for the higher light intensities likely underestimate the true rate constants (see below). Subsequent illumination with 733-nm light restored the initial Pr-state fluorescence.

We then investigated the light-triggered association reaction between *At*PhyB and P6A-mScarlet-I in the same experimental setup used for the above fluorescence experiments. Notably, the probe light employed for mScarlet-I detection (565 nm, 0.05 mW cm^−2^) induces gradual Pr→Pfr photoconversion, as seen in a slow drop of the fluorescence signal at a rate of 0.036 s^−1^ (Supplementary Fig. S5A). When red light (658 nm) was applied, the mScarlet-I fluorescence monotonically decayed, reflecting complex formation between *At*PhyB and P6A (Fig. 2B). At 1 mW cm^−2^ applied red light, a rate constant of 0.46 s^−1^ resulted which is much faster than that for the above gradual activation by the instrument probe light and in fact close to that seen for Pr→Pfr photoconversion at the same illumination conditions, see above. At this light intensity, the observable kinetics are thus severely limited by the initial photoconversion, rate constant *k*_p_, and accordingly little suited for gaining insight into the association and dissociation kinetics, rate constants *k*_a_ and *k*_d_ (Fig. 2A). We therefore increased the red-light intensity successively to 10, 30, and 69 mW cm^−2^ and observed mScarlet-I fluorescence decays with rate constants of 1.5, 2.6, and 3.6 s^−1^, respectively. Notably, the last 2 rates are considerably slower than for the Pr→Pfr photoconversion at the same light intensities, thus indicating that the observable kinetics can in principle report on the association and dissociation processes. However, the increase of red light from 1 mW cm^−2^ to higher intensities incurred 2 initially baffling phenomena. First, the amplitude of the mScarlet-I fluorescence decay diminished with increasing light fluence rate. Put another way, under constant illumination, the extent of *At*PhyB:P6A complex formation decreased with red-light intensity. Second, once illumination ceased, a secondary decrease of mScarlet-I fluorescence occurred, and the final signal was independent of the initial red-light fluence rate. These data indicate another association process in darkness which at first glance seems to run counter to numerous studies that well established the red-light activation of the *At*PhyB:PIF interaction (Shimizu-Sato et al. 2002; Li et al. 2011; Pham et al. 2018).

To rationalize these counterintuitive findings, we modeled the processes in the *At*PhyB:PIF system upon red-light exposure (see Materials and methods, equations (13 to 17)). Key to explaining the experimental data is the notion that red light not only promotes the forward Pr→Pfr conversion but also the backward Pfr→Pr reversion (Fig. 2A). Notably, the bidirectional photoconversion accounts for the mixed Pr:Pfr absorbance spectra under red light (see Fig. 1B). Although the principal aspect is hence intuitively familiar, its mechanistic implications for downstream processes and the interactions with partner proteins are less so. To obtain a quantitative understanding, we modeled the photoconversion between Pr and Pfr as a reversible first-order reaction with microscopic rate constants *k*_p_ and *k*_q_ assumed to scale linearly with the applied red-light intensity. The association between *At*PhyB and P6A-mScarlet-I was cast as a bimolecular reaction with rate constants *k*_aR_ and *k*_aFR_ in the Pr and Pfr states, respectively. Likewise, the dissociation of the *At*PhyB:P6A complex was expressed as a unimolecular reaction with rate constants *k*_dR_ in the Pr and *k*_dFR_ in Pfr state. As *At*PhyB only weakly interacts with PIF proteins in the Pr state (Golonka et al. 2019), the rate constant *k*_aR_ was set to zero. Moreover, the ratio of *k*_p_ over *k*_q_ was constrained at 0.73/0.27 to account for the Pfr:Pr proportion observed spectroscopically at photostationary state when illuminating with 658-nm light (see Fig. 1B). Notably, variation of the red-light intensity had only minor impact on the photostationary Pr:Pfr ratio of the *At*PhyB PCM (Supplementary Fig. S3B). The resulting system of ordinary differential equations (ODE) was numerically solved and used to globally fit the association kinetics acquired at red-light intensities of 1, 10, 30, and 69 mW cm^−2^. Despite using as floating parameters only 2 amplitude terms and the 4 rate constants *k*_p_, *k*_aFR_, *k*_dFR_, and *k*_dR_, the global fit described the experimental data remarkably well and quantitatively. This starkly contrasts with the simpler conventional model for light-dependent interactions that disregards light-driven dissociation of the Phy:PIF complex and utterly fails to account for the experimental data. Inspection of the global fit results reveals the likely basis for the unexpected experimental observations. Although increasing the intensity of applied red light does not alter the Pr:Pfr ratio at photostationary state (see Supplementary Fig. S3B), it does accelerate the bidirectional Pr⇄Pfr photoconversion. At high light fluence rates, the light-driven Pfr→Pr reversion becomes sufficiently fast to compete with the dynamics of *At*PhyB:P6A complex formation. Once *At*PhyB is rapidly converted to the Pr state, it can either be fast reactivated to the Pfr state, or the complex dissociates. Depending on the concentrations of *At*PhyB and P6A and the microscopic rate constants *k*_aFR_, *k*_dR_, and *k*_dFR_, the association leading to the *At*PhyB:P6A complex in the Pfr state may be slower than the dissociation of this complex within the Pr state. In the regime of strong red light, the *At*PhyB:PIF complex will hence be depleted although the Pr:Pfr ratio at photostationary state remains constant. Once illumination ceases, the association and dissociation processes play out without interference by photoconversion. This notion readily explains why the fluorescence readings after light application converged to the same value, independent of the initially applied red-light fluence rate.

Notwithstanding their pervasive use in biotechnology and optogenetics (Shimizu-Sato et al. 2002; Levskaya et al. 2009; Müller et al. 2013a, 2013b), the isolated PCMs of plant phytochromes differ from more extended protein constructs by being monomeric rather than homodimeric (Li et al. 2022). We therefore extended our interaction studies to an *At*PhyB variant that comprises the first 982 residues and thus includes the PAS1, PAS2, and DHp domains in addition to the N-terminal PCM and the NTE (see Fig. 1A). Crucially, this variant, denoted *At*PhyB (1-982), exhibits enhanced dimerization propensity (Li et al. 2022). When exposed to red light, *At*PhyB (1-982) converted to the Pfr state but to somewhat lower extent than the isolated PCM (Supplementary Fig. S3C). The Pfr:Pr distribution assumed at photostationary state was 0.29:0.71 at a red-light power of 1 mW cm^−2^. Interestingly, successively lower Pfr fractions of 0.67, 0.62, and 0.55 resulted after illumination with red light at intensities of 10, 30, and 69 mW cm^−2^, respectively. The origin of the slight decrease of Pfr proportion at higher light powers is elusive but may be linked to the dimeric nature of the extended *At*PhyB (1-982) variant. Upon exposure to continuous red light of these intensities (Fig. 2C), *At*PhyB (1-982) also underwent biphasic interaction kinetics with P6A, characterized by a first association phase during illumination, and a second ensuing after light shut off. As for the shorter *At*PhyB variant, the PIF interaction trajectories of *At*PhyB (1-982) under 1, 10, 30, and 69 mW cm^−2^ red light showed successive attenuation and could be globally described by the same mechanistic model we advanced above, thus further strengthening it. Remarkably, the attenuation of the PIF interaction during illumination at high powers was even more dramatic for *At*PhyB (1-982) compared to the PCM construct which is readily explained by its considerably slower association kinetics. These slower kinetics are directly apparent in the second phase of the experimental data after illumination ceases as a much more protracted decay of the mScarlet-I fluorescence signal. Moreover, independent analyses, reported below, confirm the much-slower association reaction of *At*PhyB (1-982).

Notably, the same mechanistic model used for the *At*PhyB PCM also accounted for the light-dependent PIF association dynamics of the *At*PhyB (1-982) variant although it neglects phytochrome dimerization. In a similar vein, this model does not regard heterogeneity within either the Pr or Pfr states which has been documented for both plant and (cyano)bacterial phytochromes (Sineshchekov 1995; Kirpich et al. 2018). Such heterogeneity may arise from differences in the protonation and conformation of the bilin chromophore and adjacent residues. It is currently unclear if these intermediate states arise in the present experiments nor whether they can still participate in photoreception and PIF interaction (i.e. they would be “on-pathway”) or not (i.e. “off-pathway”). Notably, after light shut off in the above experiments, all time traces fast coalesced to the same final fluorescence value (see Fig. 2B and C) which indicates that heterogenous intermediates, to the extent they are present, either do not much differ in their photochemical and interaction traits, or rapidly equilibrate with each other. Taken together, more complex models that explicitly include dimerization, Pr:Pfr heterodimers, and heterogeneity within either Pr or Pfr may be evidently considered in principle. Given that the simpler mechanism well describes the data, we currently deem the use of more elaborate models unwarranted.

### Light fluence modulates optogenetic responses in mammalian and plant cells

As a core tenet, the above mechanistic model (see Fig. 2A) posits that the observed attenuation under strong light is engrained in the Phy:PIF interaction. Hence, similar effects are also expected for other scenarios and applications that rely on this interaction. To put this notion to the experimental test, we investigated the response of an optogenetic circuit that harnesses *At*PhyB:PIF pairs and mediates the red-light-dependent gene expression in mammalian cells (Fig. 2D, Supplementary Fig. S6) (Müller et al. 2013a, 2013b; Golonka et al. 2019). In this setup, one of several PIF3/6 variants is linked to an erythromycin repressor DNA-binding domain (DBD) and thereby directed to the operator region upstream of a minimal promoter driving the expression of a secreted alkaline phosphatase (SEAP) reporter gene (Supplementary Fig. S6A and B). Upon red-light stimulation (660 nm), a VP16 *trans*-activation domain fused to the *At*PhyB PCM is recruited to the target operator and thereby ramps up SEAP expression. For P3A, PIF3, P6A, and PIF6, constant red light prompted up to several hundred-fold elevated SEAP activity compared to darkness (Fig. 2D). When the intensity of red light was increased beyond 1 *μ*E s^−1^ m^−2^ (corresponding to 0.018 mW cm^−2^), for the individual PIF variants the SEAP activity dropped by between 2- and 5-fold at 100 *μ*E s^−1^ m^−2^. By contrast, positive-control cells harboring VP16 directly fused to the DBD exhibited no reduction in SEAP activity over these light intensities, thus ruling out phototoxicity as the origin of the attenuation effect (Supplementary Fig. S6B). We next extended the experiments in mammalian cells to full-length *At*PhyB. When combined with the different PIF3/6 variants, full-length *At*PhyB prompted an increase of SEAP reporter activity under red light which reached a maximum at an intensity around 2 to 10 *μ*E s^−1^ m^−2^, depending on PIF variant (Fig. 2E). At higher light intensities, the SEAP reporter activity dropped again by up to 40%, overall similar as for the isolated *At*PhyB PCM but less pronounced.

We next investigated whether the attenuation observed at high light intensity also occurs in Arabidopsis protoplasts. Using a closely similar approach as in the mammalian cells, *At*PhyB PCM together with one of several PIF variants mediated the expression of a luciferase reporter (Supplementary Fig. S6C) (Müller et al. 2014). Red light at 2 *μ*E s^−1^ m^−2^ intensity elicited a several 10-fold luciferase up-regulation relative to darkness (Fig. 2F). Elevating the light intensity beyond this intensity incurred a drop in luciferase expression by factors between 1.5 and 1.8. Although less pronounced than in mammalian cells, the plant protoplasts thus also exhibited a reduction of reporter-gene expression at higher light doses. Taken together, these data concur with the above fluorescence-based analyses in mammalian cells in that the system output does not increase monotonically with light dose but passes through a maximum. In all cases, the unexpected attenuation of the output signal at higher light intensities in cellular contexts can be explained by our mechanistic model.

We further confirmed the robustness of the mechanistic model in experiments at the elevated temperatures of 22 °C and 30 °C (Supplementary Fig. S5B to E). For both the *At*PhyB PCM and (1-982), the PIF interaction exhibited attenuation under strong continuous light. Compared to 15 °C (see Fig. 2B and C), the attenuation was somewhat less pronounced, which owes to an acceleration of the association kinetics at the higher temperatures (see below).

### Temperature dependence of the *At*PhyB:PIF interaction kinetics

**Figure 3.**
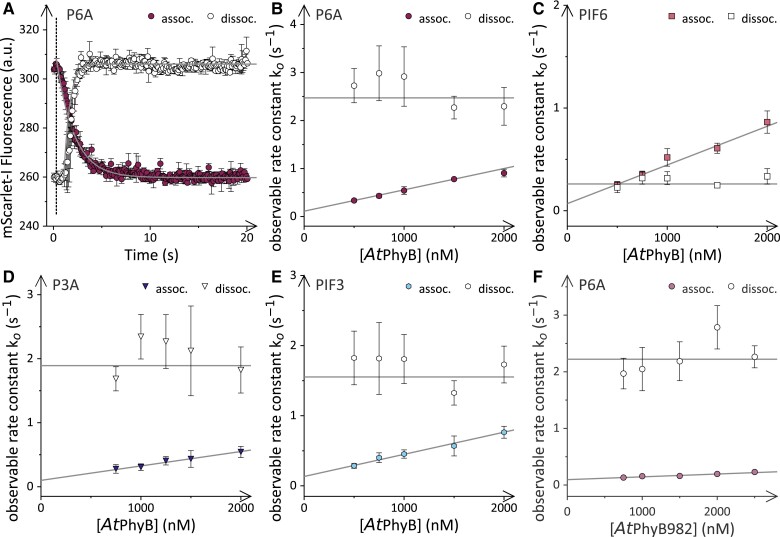
Bimolecular association and unimolecular dissociation kinetics of the *At*PhyB:PIF complex. **A)** 20 nm P6A-mScarlet-I were incubated at 15 °C in the presence of 1,000 nm  *At*PhyB PCM. Using mScarlet-I fluorescence as readout, the association kinetics (filled purple circles) were recorded upon red-light illumination (658 nm, 0.5 s, 69 mW cm^−2^), where the dashed line marks the onset of illumination. A fit of the data to a single-exponential function yielded the observable rate constant *k*_oFR_. The dissociation kinetics (open circles) were acquired under constant far-red light (733 nm, 42 mW cm^−2^) and evaluated according to a sequential reaction model to determine the observable rate constant *k*_oR_. Data in panel **A** represent mean ± SD of 3 independent experiments. **B)** The rate constants *k*_oFR_ (filled purple circles) and *k*_oR_ (open circles) for the *At*PhyB:P6A interaction upon red-light exposure as a function of *At*PhyB PCM concentration. Data correspond to mean ± SD of 3 biologically independent measurements. The lines show fits to linear functions. The bimolecular association rate constant upon red-light exposure, *k*_aFR_, determined by data fitting was adjusted for the fractional Pfr population of around 73% under these conditions (see Fig. 1B and Table 1). **C)** As panel **B** but for PIF6-mScarlet-I instead of P6A-mScarlet-I. **D)** As panel **B** but for P3A-mScarlet-I. **E)** As panel **B** but for PIF3-mScarlet-I. **F)** As panel **B** but for the interaction of P6A-mScarlet-I with *At*PhyB (1-982) rather than the *At*PhyB PCM.

The kinetic model (see Fig. 2A) pinpoints an experimental challenge for the fluorescence-based investigation of the Phy:PIF interaction dynamics, but it also identifies a solution. To sufficiently resolve the kinetics of PIF association and dissociation, it is imperative that the applied red-light dose be high such as to equilibrate the Pr⇄Pfr photoconversion fast. However, at these conditions the *At*PhyB:PIF complex is subject to rapid depletion via light-driven Pfr→Pr reversion and subsequent dissociation out of Pr, see above. As a remedy, we implemented an optical shutter to control light exposure with a time resolution of around 10 ms, thus enabling illumination for brief periods at high intensity. Of added benefit, potential contributions of the turn-on and turn-off kinetics of the LED light source are thereby eliminated. As monitored by the *At*PhyB fluorescence, the 658-nm LED set at its maximal intensity of 69 mW cm^−2^ drove the Pr→Pfr photoconversion with a rate of 10 s^−1^ (Supplementary Fig. S4B). For the subsequent experiments, we thus opted for an initial 0.5-s time window of red-light illumination and monitored the *At*PhyB:PIF association dynamics immediately afterwards (Fig. 3A). To this end, 20 nm P6A-mScarlet-I were incubated at 15 °C with increasing *At*PhyB amounts between 500 nm and 2 *μ*m. The samples were first illuminated with far-red light (733 nm) to revert *At*PhyB to its Pr state, followed by 0.5 s exposure to red light (658 nm) and measurement of the mScarlet-I fluorescence (Fig. 3A). Given the molar excess of *At*PhyB over P6A-mScarlet-I, we regarded the concentration of free *At*PhyB constant over time and evaluated the fluorescence data according to a pseudo-first-order model to determine the observable rate constant *k*_oFR_ (Fig. 3B). A linear fit of *k*_oFR_ as a function of *At*PhyB concentration allowed the determination of the bimolecular association rate constant *k*_aFR_ and the unimolecular dissociation rate constant *k*_dFR_ as the slope and ordinate offset, respectively. Doing so yielded values of *k*_aFR_ = (4.4 ± 0.2) × 10^5^ m^−1^ s^−1^ and *k*_dFR_ = (0.11 ± 0.03) s^−1^ for the *At*PhyB:P6A interaction at 15 °C. As above in the equilibrium experiments, we adjusted *k*_aFR_ for the fractional Pfr population under red light and thus determined a value of *k*_aFR_ = (6.1 ± 0.3) × 10^5^ m^−1^ s^−1^ (see Table 1). In the following, all experimentally determined association rate constants are corrected for the Pr:Pfr ratio at photostationary state in this manner. From the rate constants *k*_aFR_ and *k*_dFR_, we calculated the dissociation constant as (183 ± 48) nm, slightly weaker but in reasonable agreement with the equilibrium titration (see Supplementary Fig. S2).

**Table 1.** Interaction of AtPhyB with the PIF3 and PIF6 variants at 15 °C

PIF variant	*k*_aFR_ (m^−1^ s^−1^)^a^	*k*_dFR_ (s^−1^)	*K*_d_ (nm)^b^	*k*_dR_ (s^−1^)
P6A	(6.1 ± 0.3) × 10^5c^	0.11 ± 0.03	183 ± 48	2.5 ± 0.1
PIF6	(5.2 ± 0.4) × 10^5^	0.07 ± 0.03	133 ± 55	0.26 ± 0.02
P3A	(3.1 ± 0.4) × 10^5^	0.10 ± 0.03	322 ± 109	1.9 ± 0.1
PIF3	(4.3 ± 0.2) × 10^5^	0.13 ± 0.02	308 ± 42	1.6 ± 0.1

^a^The rate constants *k*_aFR_ have been corrected for the 0.73:0.27 Pfr:Pr ratio *At*PhyB assumes under 658-nm light (see main text).

^b^Calculated from the rate constants *k*_aFR_ and *k*_dFR_.

^c^Confidence intervals denote the asymptotic standard errors from nonlinear least-squares fitting of the underlying data.

To investigate the dissociation kinetics of the *At*PhyB:P6A complex, we first determined how fast *At*PhyB reverts from a Pr:Pfr mixture to the pure Pr state when exposed to 733-nm light. Given that this wavelength does not substantially drive the Pr→Pfr conversion, the experiments were conducted under constant 733-nm illumination without shutter control. By monitoring the weak Pr fluorescence, we observed that the light-induced Pfr→Pr reversion of *At*PhyB followed monoexponential kinetics with a rate constant *k*_q_ of 2.4 s^−1^ at the maximal LED intensity of 42 mW cm^−2^ (Supplementary Fig. S4C). We then exposed the above *At*PhyB:P6A mixtures to red light to partially populate the Pfr state, before switching to illumination with 733-nm light and recording mScarlet-I fluorescence (Fig. 3A). The observable fluorescence kinetics were fitted to a consecutive model that considers initial Pfr→Pr reversion, governed by *k*_q_, and the subsequent dissociation of the *At*PhyB:P6A complex (Golonka et al. 2020). The observable rate constant *k*_oR_ reflecting complex formation and dissolution did not vary with *At*PhyB concentration which immediately indicates that the unimolecular dissociation dominates the association reaction in the Pr state. The dissociation rate constant thus equaled *k*_oR_ and amounted to (2.5 ± 0.1) s^−1^ for the *At*PhyB:P6A complex in the Pr state at 15 °C. As this value is close to the rate constant *k*_q_ for Pfr→Pr photoconversion, our measurements likely underestimate the true speed of *At*PhyB:P6A dissociation in the Pr state severely.

**Figure 4.**
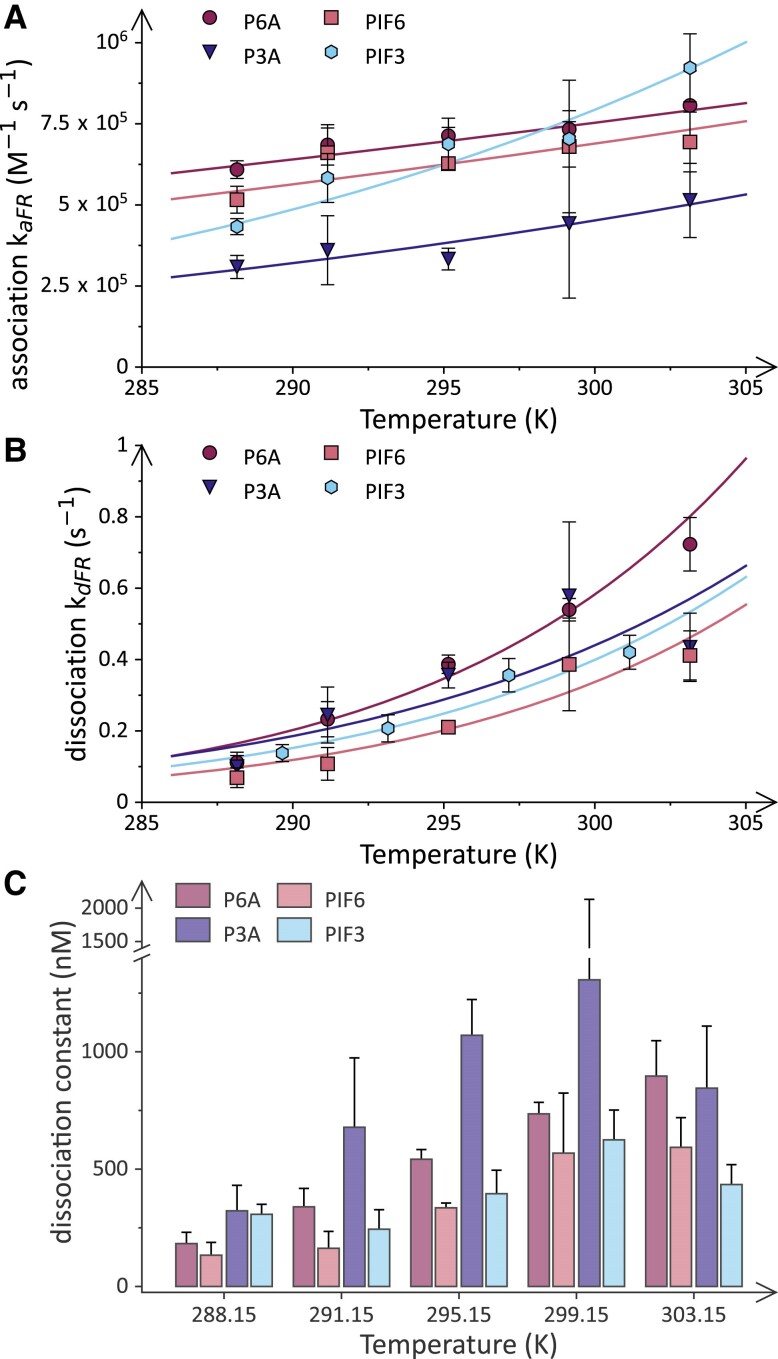
Temperature dependence of the *At*PhyB:PIF interaction dynamics. **A)** The bimolecular association rate constant *k*_aFR_ upon red-light activation as a function of temperature. Data for the P6A interaction are shown as purple circles, for P3A as blue triangles, for PIF6 as red squares, and for PIF3 as light blue hexagons. The lines denote fits to the Arrhenius equation. **B)** As in panel **A** but for the unimolecular dissociation rate constant *k*_dFR_ upon red-light activation. Data in panels **A** and **B** represent the results from nonlinear least-squares fitting of the underlying data with the error bar denoting the asymptotic standard errors. **C)** The dissociation constant *K*_d_ for the interaction of *At*PhyB in its Pfr state with P6A (purple bars, leftmost), PIF6 (red bars, second from left), P3A (blue bars, third from left), and PIF3 (light blue bars, rightmost) as a function of temperature. The *K*_d_ values were calculated as the quotient of *k*_dFR_ over *k*_aFR_ (see data in panels **A** and **B**) with propagation of the parameter errors.

To glean additional insight, we then assessed the *At*PhyB light response and its interactions with PIF partners at several temperatures up to 30 °C. Steady-state absorbance spectroscopy revealed little variation with temperature of the Pfr:Pr photostationary state the *At*PhyB PCM attains under red light (Supplementary Fig. S3D). We next probed the light-induced association and dissociation kinetics and found them to speed up with temperature, as expected (Supplementary Fig. S7). Whereas the association in the Pfr state became 1.3-fold faster between 15 and 30 °C, the corresponding dissociation reaction accelerated 6.5-fold. Consequently, the stability of the *At*PhyB:P6A complex decreased at elevated temperatures, with a dissociation constant of around 900 nm at 30 °C. The evaluation of the rate constants as a function of temperature yielded Arrhenius activation energies of (1.2 ± 0.2) × 10^4^ J mol^−1^ and (7.1 ± 0.8) × 10^4^ J mol^−1^ for the association and dissociation, respectively (Fig. 4, Table 2). Upon accounting for the decrease in solvent viscosity between 15 and 30 °C, the association rate constant hardly varied with temperature anymore. Uncertainties in the determination of the dissociation rate constant *k*_dR_ in the Pr state precluded the calculation of reliable activation energies for this process.

**Table 2.** Arrhenius activation energies of the AtPhyB:PIF interaction dynamics

PIF variant	*E_A_* (J mol^−1^)	
	Pfr association (*k*_aFR_)	Pfr dissociation (*k*_dFR_)
P6A	(1.2 ± 0.2) × 10^4a^	(7.1 ± 0.8) × 10^4^
PIF6	(1.1 ± 0.5) × 10^4^	(7.2 ± 1.7) × 10^4^
P3A	(2.5 ± 0.5) × 10^4^	(4.7 ± 2.4) × 10^4^
PIF3	(3.1 ± 0.5) × 10^4^	(5.3 ± 1.7) × 10^4^

^a^Confidence intervals denote the asymptotic standard errors from nonlinear least-squares fitting of the underlying data.

Next, we extended the analysis of the temperature-dependent association dynamics to the PIF variants PIF3, P3A, and PIF6 (Figs. 3 and 4, Supplementary Figs. S8 to S11). At all tested temperatures, PIF6 interacted with *At*PhyB somewhat more strongly [*K*_d_ = (133 ± 55) nm at 15 °C] compared to P6A which concurs with our previous analyses using a different fluorescent label (Golonka et al. 2019) (Fig. 3C and Supplementary Fig. S9). The stronger affinity can be chiefly attributed to decelerated dissociation kinetics while the association reaction also slowed down slightly, consistent with the presence of the APB.B motif in PIF6 compared to P6A (Golonka et al. 2019) (Table 1). The activation energies connected with the association and dissociation reactions in the Pfr state were closely similar for P6A and PIF6, and the underlying rate constants for these 2 PIF variants varied with temperature in lockstep (Fig. 4A and B). Compared to P6A and PIF6, the P3A and PIF3 variants exhibited weaker *At*PhyB interactions at 15 °C with *K*_d_ values on the order of 300 nm (Table 1, Fig. 3D and E and Supplementary Figs. S10 and S11). The slightly reduced affinity principally owes to slower association kinetics than for PIF6 and P6A while the dissociation kinetics were similarly fast. Intriguingly, the activation energies for the interaction of *At*PhyB with P3A or PIF3 substantially differed from those for P6A and PIF6. While the association reaction for the PIF3 variants varied more strongly with temperature than that for the PIF6 variants, the opposite proved true for the dissociation reactions. As a corollary, the affinity between *At*PhyB and P3A or PIF3 decreased to lesser extent between 15 and 30 °C by 1.4- to 2.6-fold, rather than the around 5-fold reduction in the case of P6A and PIF6.

We also probed the association dynamics of the larger *At*PhyB (1-982) protein as a function of temperature (Fig. 3F, Supplementary Fig. S12, Table 3). The Pfr:Pr ratio assumed under red-light illumination amounted to 0.55:0.45 at 15 °C but slightly increased to around 0.65:0.35 at temperatures between 18 and 30 °C (Supplementary Fig. S3E). At 15 °C, the bimolecular association proceeded with a rate constant of *k*_aFR_ = (9.0 ± 0.2) × 10^4^ m^−1^ s^−1^ which is markedly slower than the corresponding reaction for the *At*PhyB PCM. Notably, the slower association kinetics are also directly reflected in the strong attenuation of the interaction under strong continuous light evidenced above (see Fig. 2C). Owing to the slow association reaction, the affinity of *At*PhyB (1-982) for P6A is comparatively low at 1,400 nm. Over the temperature range from 15 to 30 °C, the affinity dropped by 3-fold, chiefly owing to a 5-fold higher dissociation rate constant *k*_dFR_ while *k*_aFR_ varied less strongly with temperature (Table 3, Supplementary Fig. S13).

**Table 3.** Collated parameters for the AtPhyB (1-982):P6A interaction

Parameter	Value
*k*_aFR_ (m^−1^ s^−1^)^a,b^	(9.0 ± 0.2) × 10^4c^
*E_A_ k*_aFR_ (J mol^−1^)	(2.3 ± 0.4) × 10^4^
*k*_dFR_ (s^−1^)^b^	0.095 ± 0.01
*E_A_ k*_dFR_ (J mol^−1^)	(7.5 ± 0.6) × 10^4^
*K*_d_ (nm)^d^	1,050 ± 240
*k*_dR_ (s^−1^)^b^	2.2 ± 0.1

^a^The rate constant *k*_aFR_ has been corrected for the 0.55:0.45 Pfr:Pr ratio adopted under 658-nm light.

^b^Determined at 15 °C.

^c^Confidence intervals denote the asymptotic standard errors from nonlinear least-squares fitting of the underlying data.

^d^Calculated from the rate constants *k*_aFR_ and *k*_dFR_.

### Temperature effects on diffusion and bimolecular encounter rates

**Figure 5.**
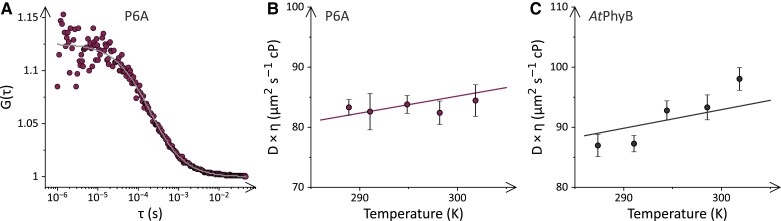
Hydrodynamics of *At*PhyB and PIF variants at different temperatures. **A)** Fluorescence autocorrelation of 20 nm P6A-mScarlet-I at 15 °C. Fluorescence was detected at 510 nm excitation through a 526-nm long-pass emission filter. **B)** Diffusion coefficients of P6A-mScarlet-I multiplied by viscosity at different temperatures. Data represent mean ± SD of 3 measurements. The line denotes a fit to the Stokes–Einstein equation. **C)** As in panel **B** but for *At*PhyB. Fluorescence was excited at 640 nm and detected through a 659-nm long-pass filter.

We next addressed to what extent the different association dynamics and temperature responses evident among the PIF3 and PIF6 variants owe to differences in the diffusion of these proteins (Fig. 5). All other factors equal, the bimolecular association reaction is expected to inversely scale with solvent viscosity. We employed fluorescence-correlation spectroscopy (FCS) to determine the transversal diffusion coefficients for P3A, PIF3, P6A, and PIF6. A total of 20 nm of each variant, labeled with mScarlet-I as before, were incubated at different temperatures, and fluorescence was recorded over 3 min. Diffusion constants were determined based on the autocorrelation of the fluorescence data. For the P3A and P6A variants, diffusion coefficients *D* around 80 *μ*m^2^ s^−1^ resulted (Fig. 5B and Supplementary Fig. S14B). The slight increase of *D* with temperature was evaluated according to the Stokes–Einstein equation while assuming spherical particle shape. This evaluation yielded a hydrodynamic radius *R*_h_ of 2.3 nm for both P3A-mScarlet-I and P6A-mScarlet-I (Supplementary Table S1) which is consistent with the known molecular dimensions of the fluorescent protein (Bindels et al. 2017). For PIF3, we determined lower diffusion coefficients around 75 *μ*m^2^ s^−1^ and a larger hydrodynamic radius of 2.7 nm (Supplementary Fig. S14C), in line with the bigger size of this PIF variant. In case of PIF6 (Supplementary Fig. S14A), yet lower diffusion coefficients around 58 *μ*m^2^ s^−1^ and a higher *R*_h_ of 3.2 nm resulted. The substantially different parameters are likely due to dimerization of this PIF variant as observed previously (Golonka et al. 2019). We also performed FCS measurements on *At*PhyB and determined diffusion coefficients between 88 *μ*m^2^ s^−1^ at 16 °C and 98 *μ*m^2^ s^−1^ at 29 °C (Fig. 5C). Notably, *D* thus increased more strongly with temperature than expected based on the Stokes–Einstein relation. We entertained several potential explanations for these observations. First, owing to the weak quantum yield of the Pr-state fluorescence, the FCS experiments required a relatively high *At*PhyB concentration of 200 nm. Hence, the variation of the diffusion coefficient with temperature might be connected to a change in the monomer-dimer equilibrium of the *At*PhyB PCM (Golonka et al. 2019). However, the amplitude of the FCS autocorrelation function, which is inversely proportional to the number of diffusing molecules within the focal volume, remained constant with temperature, which speaks against changes in the *At*PhyB oligomeric state. Second, the red laser light required for monitoring the Pr-state fluorescence can also drive the Pr⇄Pfr photoconversion which may compromise data evaluation. Measurements at 3-fold reduced laser power however yielded similar values for *D* at the different temperatures, thus making it unlikely that Pr⇄Pfr photoconversion significantly impacted these experiments. Third, *At*PhyB might undergo refolding across this temperature range. However, circular dichroism spectroscopy revealed little conformational changes over the relevant range and identified a midpoint of thermal unfolding of (49.9 ± 0.2)°C, well above the highest temperature presently assessed (Supplementary Fig. S15). More subtle temperature-dependent structural and dynamic changes can however not be ruled out. Although the molecular reasons underlying the moderate deviation from linearity remain unclear, we evaluated the data at the different temperatures according to Stokes–Einstein and calculated an apparent *R*_h_ = 2.5 nm.

Knowledge of *D* and *R*_h_ for the interacting proteins permits the calculation of diffusional encounter rates according to Smoluchowski (1918). This evaluation yielded closely similar bimolecular encounter rates around 5.6 × 10^6^ m^−1^ s^−1^ at 15 °C for all 4 *At*PhyB:PIF combinations studied at present (Supplementary Table S1). The diffusional collision rates are therefore between 9 to 12 times larger than the experimentally determined rate constants for the bimolecular association reaction of *At*PhyB in its Pfr state with the PIF variants P6A, PIF6, and PIF3 (see Table 1). By contrast, the bimolecular association rate constant for the P3A interaction was around 18-fold lower than the diffusional encounter rate. At elevated temperatures, the diffusional encounter rate increased in lockstep with the bimolecular association rate constant for P6A and PIF6. These data reflect that for these 2 PIF variants the temperature dependence of the association reaction can be fully ascribed to the decrease in solvent viscosity at higher temperatures, as already noted above. By contrast, in the case of P3A and PIF3 the ratio of encounter over association rate constant decreased successively, indicating that temperature increases favor productive diffusional encounters and thereby the formation of the *At*PhyB:PIF complex, again consistent with the above activation energy analyses.

In the case of the *At*PhyB (1-982) variant, *R*_h_ amounted to 4.0 nm which is consistent with the larger protein size and enhanced dimerization propensity compared to the *At*PhyB PCM (Supplementary Fig. S13D). Based on this value, we calculated a bimolecular encounter rate between *At*PhyB (1-982) and P6A of 5.9 × 10^6^ m^−1^ s^−1^ at 15 °C, similar to the above rates for the *At*PhyB PCM. Intriguingly, the much-slower association kinetics of *At*PhyB (1-982) (see Fig. 3F, Table 3) relative to the *At*PhyB PCM therefore do not owe to overall reduced diffusional encounters but to a lower number of productive ones.

## Discussion

### Phytochrome:PIF interactions track the temperature

**Figure 6.**
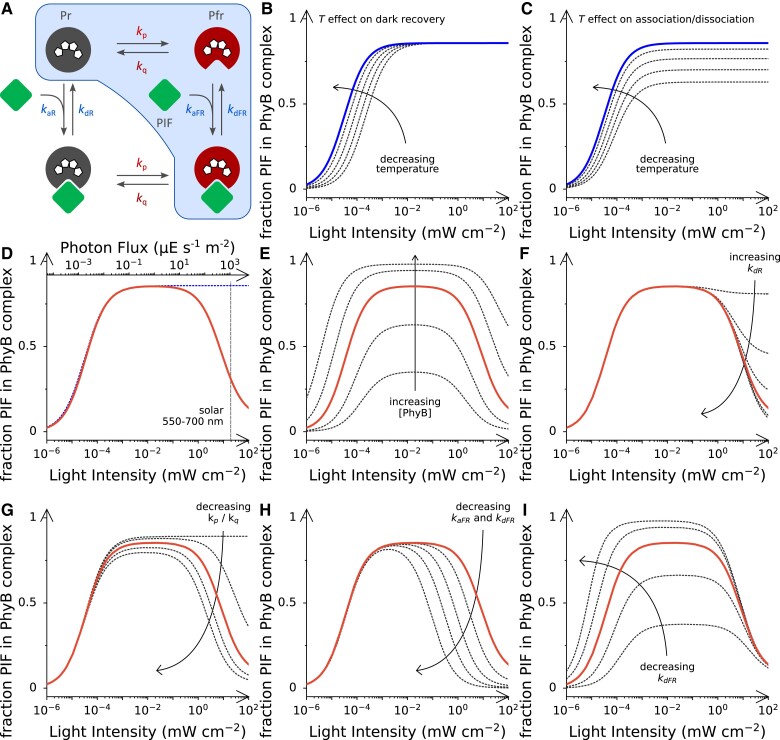
Interplay of light and temperature signals in plant phytochromes and interacting factors. **A)** Schematic of the *At*PhyB:PIF interaction dynamics. The blue box denotes the conventional, simplified scheme that neglects Pr⇄Pfr interconversion of AtPhyB when in complex with PIF. The simplified scheme underpins the simulations in panels **B** and **C**, and the full scheme underlies those in panels **D** to **I**. In panel **B** and all subsequent ones, the logarithmic abscissa denotes the applied red-light intensity (658 nm), and the ordinate shows the proportion of the PIF protein in complex with the *At*PhyB PCM. Due to much higher PIF affinity of the Pfr state vs. the Pr state, essentially all *At*PhyB molecules in the PIF complex reside in their Pfr state. Unless stated otherwise, all calculations are based on the experimentally determined parameters for the interaction between the *At*PhyB PCM and PIF6 at 15 °C (Supplementary Table S2). **B)** Impact of temperature on dark-recovery rate and PIF complex formation. Note that only the dark recovery is assumed to vary with temperature (solid blue line: 15 °C; dotted black lines: 18, 22, 26, and 30 °C), but all other parameters remain invariant. **C)** Impact of temperature on the Phy:PIF interaction dynamics in the Pfr state. The simulation assumes that only the rate constants *k*_aFR_ and *k*_dFR_ increase with temperature (lines for different temperatures as in panel **B**), but all other parameters stay constant. **D)** Simulation of the full kinetic model (solid red curve) with the parameters determined for the *At*PhyB:PIF6 interaction at 15 °C. All subsequent panels **E** to **I** reproduce the solid red curve as a reference point. The dotted blue line refers to the simplified model, using the same simulation parameters but not considering Pr⇄Pfr interconversion of the *At*PhyB:PIF complex (see panel **A**). The top axis shows the photon flux per area corresponding to a given light intensity (calculated for a wavelength of 660 nm). The vertical dashed line marks the integrated solar spectral power between 550 and 700 nm. **E)** Impact of *At*PhyB concentration on PIF complexation. From top to bottom, the dotted curves represent simulations with relative PhyB concentrations of 0.1, 0.3, 1 (solid red curve), 3, and 10. **F)** Impact of the dissociation rate *k*_dR_ on PIF complexation. From top to bottom, the underlying simulations vary *k*_dR_ by factors of 0.01, 0.1, 0.3, 1 (solid red curve), 3, and 10. **G)** Impact of light quality on PIF complexation. From top to bottom, the curves show simulations assuming ratios of *k*_p_/*k*_q_ of ∞ (i.e. *k*_q_ = 0), 0.11, 0.37 (i.e. 0.27/0.73, solid red curve), 0.67, and 1. **H)** Impact of the interaction kinetics on PIF complexation. From top to bottom, the simulations underlying the curves assume complexation dynamics in the Pfr state (i.e. rate constants *k*_aFR_ and *k*_dFR_) that are slower by factors of 3, 10, 30, and 100 than in the reference curve (solid red line). **I)** Impact of binding affinity on PIF complexation. From top to bottom, the curves use values of the rate constant *k*_dFR_ that are varied by factors of 0.1, 0.3, 3, and 10, relative to the reference curve (solid red line).

The past years have delivered overwhelming evidence that PhyB, and possibly other Phys, too, doubles as a thermoreceptor beyond its well-established and pivotal role as a photoreceptor (Jung et al. 2016; Legris et al. 2016; Hayes et al. 2021). A core component of PhyB thermoreception is the pronounced temperature dependence of the dark-recovery reaction from the photoactivated Pfr state to the Pr state which accelerates by more than 10-fold between 4 and 27 °C (Jung et al. 2016). All other things being equal, a faster dark-reversion rate translates into a lower Pfr population at photostationary state for a given, constant light intensity (Fig. 6A and B). Given that diverse physiological red-light responses hinge on light-dependent Phy:PIF interactions, we here investigated whether these interactions can principally contribute to thermoreception. Using a bottom-up approach, we assessed the kinetics of formation and dissolution of the *At*PhyB:PIF complex upon exposure to red and far-red light, respectively. Whereas the moderate temperature variation of the association reaction could be largely ascribed to changes in solvent viscosity, the dissociation reaction sped up several fold between 15 and 30 °C, thus incurring a concomitant reduction in binding affinity. Evidently, a reduced affinity means that a higher proportion of photoactivated *At*PhyB in its Pfr state is required to achieve binding of the PIF partner to the same extent (Fig. 6C). Increases in light intensity may thus not only be antagonized by the acceleration of the Pfr→Pr reversion with temperature, as noted previously (Jung et al. 2016; Legris et al. 2016), but also by the temperature-induced destabilization of the Phy:PIF complex. Put another way, the Phy:PIF interaction may thus amplify the temperature effect on photoreception. As a caveat, light power may not be increased indiscriminately, lest attenuation of the Phy:PIF interaction sets in, which we discuss further below.

Does it matter? This question can be answered in the affirmative where optogenetics is concerned. The substantial temperature sensitivity of the PhyB:PIF interaction, previously entirely uncharted at the molecular level, impacts on pertinent optogenetic applications which are frequently conducted at or near 37 °C in animal cells. The now available information on the temperature response of the Phy:PIF interaction stands to decisively aid in devising and evaluating such experiments. Although it is considerably harder to assess if and to what extent the Phy:PIF interaction contributes to the processing of temperature signals in plants, at least 2 principal aspects argue in favor. First, significant temperature effects on the interaction equilibria and kinetics presently manifested in a reductionist system comprising merely the *At*PhyB PCM and interacting PIF3/6 variants. The temperature responses hence seem intrinsic and likely of relevance in the much more complex environs in planta as well. At the very least, the influence of temperature on the Phy:PIF interaction should be considered when studying and evaluating the interplay of light and temperature sensing in plants. Second, the temperature effects on the binding kinetics and equilibria markedly differed for the PIF3 and PIF6 variants, with the latter being more strongly affected. This intriguing finding indicates that individual PIFs, and scores of other interactors (Cheng et al. 2021; Kim et al. 2023), may underlie varied and nuanced responses to temperature variations. Taken together, we therefore propose that Phy:PIF interactions significantly contribute to thermoreception in plants and thereby add an extra control layer for physiological adaptations in individual tissues, at different developmental stages, and depending on environmental conditions. Compared to the temperature variation of the dark recovery (Jung et al. 2016; Legris et al. 2016), this additional effect plays out at the level of the Phy:PIF interaction. As not least suggested by the substantially different properties of PIF3 and PIF6 uncovered presently, the temperature response may be individualized across Phy interaction partners and thus enable stratified physiological adaptations.

The temperature dependence of the Phy:PIF interaction may thus add yet another facet to the integrated, decentralized signal network underpinning thermosensing in Arabidopsis and other plants (Kerbler and Wigge 2023). Among numerous other interactors and transcription factors, key roles have been attributed to several PIFs, in particular PIF4 and PIF7. At elevated temperatures, phytochrome B exhibits faster recovery to its Pr state (Jung et al. 2016; Legris et al. 2016) which promotes PIF4 accumulation and thereby enables the expression of temperature-responsive genes (Qiu et al. 2019; Lee et al. 2020). A thermosensitive riboswitch in the 5′ region of the *PIF7* mRNA contributes to rising PIF7 levels at elevated temperatures and the subsequent transcription of target genes (Chung et al. 2020). The action and regulatory activity of PIF4 is further modulated by ELF3 which coalesces into liquid droplets with rising temperature (Jung et al. 2020). Our present study raises the question whether temperature effects akin to those uncovered presently for the interaction of PIF3 and PIF6 with *At*PhyB extend to other PIF components. Although we possess no pertinent data at this point, we anticipate that this is the case. That is because protein:protein interactions are commonly associated with a negative reaction enthalpy, i.e. they are exothermic. A weakening of such interactions, e.g. between *At*PhyB and PIF4, with temperature is hence fully expected. The magnitude of such effects may well differ substantially between PIFs as indicated by the disparate traits of PIF3 vs. PIF6. Future efforts should thus be directed at unraveling in molecular detail how temperature affects the interaction of plant phytochromes with diverse PIFs.

### Phytochrome:PIF interactions offer an adaptable mechanism of photoreception

As outlined above, temperature and light sensing in plants are intricately interrelated, with PhyB as a nexus for signal reception and integration (Legris et al. 2019; Kerbler and Wigge 2023). Put simply, raising the temperature antagonizes PhyB photoactivation and downstream responses. As our above analyses now reveal, the interplay between temperature and light cues not only unfolds at the stage of PhyB dark recovery (Jung et al. 2016; Legris et al. 2016) but extends to interactions with PIF partners which weaken at higher temperatures. While studying the dynamics of these interactions, we discovered a peculiar, hitherto unappreciated phenomenon manifesting under strong light. In this regime, increases in light intensity counterintuitively attenuated Phy:PIF complex formation at photostationary state. Moreover, Phy:PIF binding spiked upon cessation of illumination. A simple mechanistic model could quantitatively account for and thereby rationalize the initially baffling observations (Fig. 6A). Owing to the bathochromic shift of the Pfr absorbance spectrum (see Fig. 1B), red light by necessity not only drives Pr→Pfr photoconversion but also the Pfr→Pr photoreversion, if to lower extent. While an increase in red-light intensity does not significantly alter the Pr:Pfr proportion at photostationary state (see Supplementary Fig. S3B), it speeds up how fast each *At*PhyB molecule transitions between its Pr and Pfr states. Once a *At*PhyB molecule in complex with PIF thereby samples the Pr form, if ever so briefly, it is subject to rapid dissociation because the stability of the complex is low in this state. Evidently, the Phy:PIF complex can be regenerated subsequently by photoconversion to the Pfr state and bimolecular association with the PIF partner. However, if these processes take longer than the preceding unimolecular dissociation of the Phy:PIF complex, a net reduction of the fraction of PIF in complex with *At*PhyB results. When light shuts off, PhyB in its Pfr state associates with the PIF protein unperturbed by the light-driven Pr⇄Pfr interconversion, thus explaining the observed spike in binding.

As before, the question begs whether these processes are specific to the current study or more widely pertain to optogenetics and plant photoreception. Since optogenetic stimulation is often performed at relatively high light intensities, the above effect is expected to also impinge on pertinent experiments. Unless taken into account, this effect may lead to inadvertent application of excessive light doses and concomitant attenuation of the intended physiological perturbation. Therefore, researchers applying plant phytochromes (and other photochromic receptors, see below) for optogenetics should be mindful of the principal phenomenon. As a ready remedy, pulsatile illumination (Hennemann et al. 2018) may be implemented because the attenuation occurs under constant light but quickly dissipates when illumination terminates. Notably, the metastable Pfr state of *At*PhyB persists in darkness for between minutes and hours depending upon temperature (Jung et al. 2016). Truncation to the PCM, as at present and in most optogenetic experiments, incurs a slowdown of dark recovery (Li et al. 2022) which benefits the application of pulsed illumination schemes. The attenuation of the Phy:PIF interaction under high light need not necessarily be a bane for experiments but can be a boon. As a case in point, a recent study leveraged the acceleration of Pr⇄Pfr photoconversion kinetics with light intensity to probe ligand binding to a T-cell receptor (Yousefi et al. 2019). By varying the strength of applied red light, a desired lifetime of the receptor:ligand complex could be dialed in and the effect on downstream processes assessed. With this unparalleled experimental tool at hand, Schamel and colleagues (Yousefi et al. 2019) acquired evidence that T-cell receptors discriminate between self and foreign antigens by a kinetic proof-reading mechanism where not only the overall affinity but foremost the lifetime of the receptor:ligand complex governs downstream immune responses.

An arguably even more interesting question is to which extent the unusual dependence of the *At*PhyB:PIF interaction on light intensity is relevant in nature. We now propose that there are potentially significant contributions to plant photoreception, especially in the R-HIR regime, and to the integration with temperature cues. Notably, the attenuation of the Phy:PIF interaction at high light intensity currently appeared in a defined system with a minimal number of components. To permit principally charting and quantitatively understanding the attenuation effect, our study mainly focuses on truncated versions of *At*PhyB and the PIF proteins rather than the full-length proteins that occur in planta. Apart from a decelerated dark recovery (Li et al. 2022), the isolated PCM also differs from full-length *At*PhyB by being monomeric rather than dimeric. In addition to Pr:Pr and Pfr:Pfr homodimeric states, the full-length photoreceptor can accordingly also assume a mixed Pr:Pfr heterodimer which however recovers to the fully dark-adapted Pr:Pr homodimer comparatively rapidly (Klose et al. 2015; Smith et al. 2016; Legris et al. 2019). Against this backdrop, we expanded our analyses of the PIF interaction to the larger *At*PhyB (1-982) protein which retains several C-terminal domains of the full-length receptor and is hence capable of homodimerization. Strikingly, this *At*PhyB variant exhibited attenuation of its interaction with P6A under continuous strong light, as did the isolated PCM, but to an even more pronounced degree. Moreover, the attenuation effect played out for full-length *At*PhyB embedded within an optogenetic circuit in mammalian cells. Taken together, we consider the attenuation at high light intensity intrinsic to the Phy:PIF interaction and expect it to manifest in other phytochromes, contexts, and cellular environments, too, including inside plants.

To get a deeper mechanistic understanding and to assess in which parameter regimes the attenuation becomes relevant, we derived an analytical solution for the photostationary state which the Phy:PIF interaction system attains under constant light (see Materials and methods). This solution allows to address the pivotal question whether the light-induced attenuation of the Phy:PIF interaction takes place at physiological light intensities that a plant is likely to encounter in nature. To this end, we plugged in the parameters experimentally determined in this work for the *At*PhyB:PIF6 interaction dynamics at 15 °C (Supplementary Table S2). Doing so reveals that the attenuation of the interaction sets in at light powers of around 0.1 mW cm^−2^, roughly corresponding to a photon flux density of 10 *μ*E s^−1^ m^−2^, and levels off at intensities of 10 mW cm^−2^ and above (Fig. 6D). Although on the high side, these light powers are well within the range of relevant intensities in the environment. During the day, the integrated solar power density between 550 and 700 nm, where the Pr state preferentially absorbs, amounts to around 20 mW cm^−2^, as measured at 37° latitude and averaged over the entire year (Gueymard 2004) (Fig. 6D). We conclude from these data that the high-light-mediated attenuation effect principally bears on plant physiology and PhyB photoreception, in particular within the R-HIR regime.

We next investigated how variations in the PhyB concentration, e.g. occurring in plants during diurnal and developmental cycles (Legris et al. 2019), impact on the interaction with PIF and its light dependence. As expected, the extent of PIF complex formation monotonically increases with PhyB concentration at all light intensities (Fig. 6E). Intriguingly, reducing the PhyB concentration sharpens the light-response profile and causes attenuation to set in at lower light powers. We also assessed how the kinetics of Phy:PIF dissociation in the Pr state, reflected by the value of *k*_dR_, affect the degree of PIF binding at photostationary state (Fig. 6F). Strikingly, variation of *k*_dR_ modified the magnitude of the attenuation without changing the intensity regime in which this effect occurred. For *k*_dR_ approaching zero, the attenuation gradually dwindled away. To gauge the influence of light quality on the attenuation effect, we varied the ratio of the forward and reverse rate constants *k*_p_ and *k*_q_ for Pr⇄Pfr photoconversion (Fig. 6G). For instance, if light of longer wavelength is applied, the Pfr→Pr reversion speeds up relative to the Pr→Pfr conversion, and consequently the ratio of *k*_p_ over *k*_q_ decreases. Our simulations reveal that an acceleration of *k*_q_ relative to *k*_p_ amplifies the attenuation effect and shifts it to lower light intensities. More generally, our simulations also demonstrate that the attenuation mechanism is surprisingly robust with regard to light quality. Provided that the incident light drives the reverse Pfr→Pr conversion to at least some extent (i.e. *k*_q_ ≠ 0), attenuation will set in at sufficiently high light doses.

As not least indicated by the present study, plant phytochromes engage with diverse partner proteins that differ in their thermodynamic parameters underpinning the interaction and its dependence on light, temperature, and other factors. We hence asked how the attenuation effect would play out for (hypothetical) interactors that differ from PIF6 in the equilibrium and kinetics of the interaction with *At*PhyB. To this end, we simulated the light response for scenarios of slower association and dissociation kinetics in the Pfr state. We first modulated these kinetics in lockstep by varying *k*_aFR_ and *k*_dFR_ synchronously, thereby changing the interaction dynamics but not the overall affinity (Fig. 6H). Doing so entails a narrowing of the light-response curve and a shift of the attenuation effect toward lower light intensities. For instance, in the case of 10-fold decelerated interaction dynamics, substantial attenuation already occurred at light intensities below 0.1 mW cm^−2^, roughly corresponding to a photon flux rate of 10 *μ*E s^−1^ m^−2^. Interestingly, this is exactly the scenario that we presently observe for *At*PhyB (1-982) which exhibits markedly slower bimolecular PIF association than the isolated *At*PhyB PCM (see Fig. 3F) and accordingly more pronounced attenuation of the PIF interaction under strong light (see Fig. 2C). By contrast, variation of the dissociation rate constant *k*_dFR_ alone left attenuation essentially unchanged but modified the affinity for *At*PhyB. As a corollary, the rising flank of the response curve moved to lower light intensities for smaller *k*_dFR_ values (Fig. 6I). Jointly, these simulations indicate that the rising and falling flanks of the light-response curve can be varied through alterations of the association and dissociation kinetics. Notably, such variations may result inside the cell, where these parameters may be substantially different from the ones in the test tube determined at present. In specific, intracellular compartmentalization, macromolecular crowding, and active transport processes all stand to affect the diffusional encounter and complex formation of the Phy:PIF partners.

### Implications for photoreception in plants and beyond

Taken together, we presently identify and analyze a hitherto disregarded mode of plant phytochrome photoreception at high light intensities. As uncovered by our experimental data and simulations, the attenuation of light responses stands to occur at physiologically relevant light intensities. Light-dependent attenuation emerges as a versatile and malleable photoreception mechanism that can be adapted to different intensity regimes. Intriguingly, plant phytochromes and their interacting factors can be thereby rendered responsive to discrete light bands (see, e.g. Figure 6H), as opposed to eliciting responses that monotonically ramp up with intensity (see, e.g. Figure 6B). Moreover, the nature and magnitude of the attenuation not only depend on the properties of the phytochrome alone but also on those of the interacting factor. Combined with different interacting factors (Pham et al. 2018; Cheng et al. 2021; Kim et al. 2023), a single photoreceptor can thereby elicit diverse, fine-tuned, and stratified responses, analogous to our considerations above for the diverging temperature responsiveness of individual PIF variants. Whether and to what extent plants indeed harness the attenuation mechanism to shape their responses to environmental light cues remains the subject of further investigation. Again, the question begs to what extent the present findings for PIF3 and PIF6 extend to other PIFs and additional interacting factors that jointly form the integrated network responsible for sensing light and temperature cues in plants (Kerbler and Wigge 2023). In this regard, it is important to note that the high-light attenuation effect identified here is engrained in phytochromes as it principally owes to their bidirectional Pr⇄Pfr interconversion under red light. By that token, we expect that other interactions between phytochromes and partner proteins are subject to qualitatively similar attenuation phenomena as currently uncovered. The conditions at which attenuation sets in and its scope will depend on the interaction parameters and the relevant intracellular concentrations. Not least because of that, it is currently unclear to which extent the high-light attenuation effect governs physiological responses in plants.

In closing, we note that the light-dependent attenuation pinpointed at present for plant phytochromes may more broadly apply to photoreceptors. At least 2 core requirements for attenuation emerge. First, the photoreceptor in question be photochromic in that light drives the bidirectional interconversion between 2 functional states. Given the usually considerable overlap of the action spectra associated with these states, a single light color can suffice for driving the bimodal interconversion, if to different extent in each of the 2 directions. Second, the downstream physiological effect occur after a delay such that it cannot instantaneously track the rapid light-driven interconversion between the functional states. In the case of plant phytochromes and their interacting factors, this delay is generated by their bimolecular association reaction, but for other photoreceptors different processes may apply. Equipped with a principal understanding of the underlying processes, researchers may exploit this phenomenon for applications of diverse photoreceptors in optogenetics, biotechnology, and basic research. Likewise, light-dependent attenuation may also contribute to the natural physiological responses elicited by these same photoreceptors.

## Materials and methods

### Molecular biology

The expression plasmids pYC15 and pYC16 encoding Arabidopsis (*A. thaliana*) PIF6 (residues 1 to 100) and PIF3 (1 to 100), respectively, fused to a C-terminal mScarlet-I fluorescent protein via a DSAGSAGSAG linker, were generated by Gibson assembly (Gibson et al. 2009) in a modified pET-19b vector with altered translation-initiation region (Shilling et al. 2020; García de Fuentes and Möglich 2024) (Supplementary Tables S3 and S4). To this end, the relevant gene fragments were amplified by PCR with previous PIF3/6 expression constructs (Golonka et al. 2019) and an *Escherichia coli* codon-optimized mScarlet-I gene (GeneArt, Regensburg, Germany) as templates. Cloning into the pET19b vector added an N-terminal hexa-histidine-SUMO tag and put expression under the control of a T7-*lacO* promoter. The shortened P6A-mScarlet-I (residues 14 to 53) and P3A-mScarlet-I (residues 14 to 55) expression constructs, identifiers pYC14 and pYC19, respectively, were generated by PCR amplification from pYC15 and pYC16 and cloning into the modified pET19b vector via Gibson assembly. An mScarlet-I expression construct (pYC38) was created likewise. A gene encoding Arabidopsis PhyB (residues 1 to 982) was synthesized with codons optimized for *E. coli* expression and cloned by Gibson assembly into the pCDFDuet1 vector (Novagen, Merck, Darmstadt, Germany) to generate the expression plasmid pYC63. The sequences of all plasmids were confirmed by Sanger DNA sequencing (Microsynth seqlab, Göttingen, Germany).

### Protein expression and purification

Protein expression was done in *E. coli* LOBSTR cells (Andersen et al. 2013), followed by purification by immobilized metal ion affinity chromatography (IMAC) as described before (Golonka et al. 2019; Golonka et al. 2020). Briefly, for the expression of the Arabidopsis PhyB PCM and PhyB (1-982) with C-terminal hexa-histidine tags, the plasmids pDG458 and pYC63 were used, respectively, which additionally encode the *Synechocystis* sp. heme oxygenase 1 and PcyA to produce the phycocyanobilin chromophore. LOBSTR cells containing pDG458 or pYC63 were cultivated in terrific broth (TB) medium supplemented with 100 *μ*g mL^−1^ streptomycin at 37 °C and 225 rpm agitation in darkness. After the optical density at 600 nm (OD_600_) reached 0.6, 1 mm β-D-1-thiogalactopyranoside (IPTG) and 0.5 mm δ-aminolevulinic acid were added. The cultivation temperature was lowered to 18 °C, and the cells were incubated for 40 h in darkness. Cells were harvested by centrifugation, resuspended in buffer (50 mm Tris/HCl pH 8.0, 20 mm NaCl, 20 mm imidazole; supplemented with protease inhibitor mix [cOmplete Ultra, Roche Diagnostics, Mannheim, Germany]), and lysed by sonication. The lysate was cleared by centrifugation (30 min, 18,000 rpm) and purified by IMAC on 5-mL Protino Co^2+^-NTA columns (Macherey & Nagel, Düren, Germany) on an Äkta prime instrument. The protein was eluted with an imidazole gradient from 20 to 500 mm, and the elution fractions were analyzed by denaturing polyacrylamide gel electrophoresis (PAGE). The PAGE buffers were supplemented with 1 mm Zn^2+^ to allow detection of covalently incorporated bilin chromophores via zinc-induced fluorescence (Berkelman and Lagarias 1986). Fractions were pooled and dialyzed overnight into AEX buffer (20 mm Tris/HCl pH 8.0, 50 mm NaCl, 5 mm 2-mercaptoethanol). Samples were then applied to a HiTrap Q HP 1-mL anion-exchange column (GE Healthcare Europe GmbH, Freiburg, Germany), and eluted with 2 subsequent gradients from 50 mm to 300 mm NaCl, and from 300 mm to 500 mm NaCl. Eluted fractions were analyzed by PAGE, and suitable fractions were pooled, dialyzed against storage buffer (20 mm Tris/HCl pH 8.0, 20 mm NaCl, 10% [w/v] glycerol), and stored at −80 °C.

The purification of mScarlet-I and the PIF-mScarlet-I variants was conducted similarly with the following exceptions. No δ-aminolevulinic acid was added, and incubation after induction continued at 16 °C for 12 h. After the first IMAC, the N-terminal His_6_−SUMO was cleaved overnight at 4 °C during dialysis into 50 mm Tris/HCl pH 8.0 and 50 mm NaCl using SENP2-protease. The His_6_-SUMO tag and SENP2-protease were removed by a second IMAC, and the flow-through containing the *At*PIF3/6 construct was collected and analyzed by SDS-PAGE. Depending on purity, the proteins were optionally further purified by anion-exchange chromatography as described above. Pure *At*PIF3/6-mScarlet-I variants were dialyzed into storage buffer and stored at −80 °C.

### UV–vis absorbance spectroscopy

The purified proteins were analyzed at 22 °C by absorbance spectroscopy on an Agilent 8453 UV–visible spectrophotometer (Agilent Technologies, Waldbronn, Germany) equipped with Peltier temperature control. Concentrations of mScarlet-I, PIF3-mScarlet-I, PIF6-mScarlet-I, P3A-mScarlet-I, and P6A-mScarlet-I were determined using an extinction coefficient of 104,000 m^−1^ cm^−1^ at 569 nm (Bindels et al. 2017). For the *At*PhyB PCM and *At*PhyB (1-982), the concentration was determined at the isosbestic point at 672 nm using an extinction coefficient of 47,600 m^−1^ cm^−1^ (Golonka et al. 2019). To monitor the Pr⇄Pfr photoconversion (see Fig. 1B and Supplementary Fig. S1A and B), absorbance spectra of the *At*PhyB PCM were recorded prior to and after illumination with 658-nm and 733-nm light-emitting diodes, respectively. Both the red light and far-red light intensities were 30 mW cm^−2^ with an illumination period of 20 s. All light intensities were determined with a model 842-PE power meter (Newport, Darmstadt, Germany) equipped with a model 918D-UV-OD3 silicon photodetector (Newport). The Pr/Pfr ratio after RL illumination was determined according to (Butler et al. 1964).

The red-light-induced photoactivation of the *At*PhyB PCM and *At*PhyB (1-982) was also monitored by absorbance measurements on a Cary 60 UV–visible spectrophotometer (Agilent Technologies), equipped with Peltier temperature control (see Supplementary Fig. S3). Absorbance spectra for the 2 proteins were recorded at temperatures between 15 and 30 °C after saturating illumination with either 69 mW cm^−2^ 658-nm light (to partially populate the Pfr state) or 42 mW cm^−2^ 733-nm light (to convert to the Pr state). The kinetics of the Pr→Pfr photoconversion was recorded for the 2 proteins at 15 °C by absorbance measurements at 715 nm under continuous 658-nm light at intensities of 1, 10, 30, and 69 mW cm^−2^. The data were fitted to single-exponential functions using Fit-o-mat (Möglich 2018):


(1) $$F(t)\;=F_{0}+F_{1}\times \exp(-k_{1}\times t)\;$$

where *F_i_* are absorbance amplitudes and *k*_1_ represents the rate constant for Pr→Pfr photoconversion at a given red-light intensity.

The dark recovery of the *At*PhyB PCM was recorded at wavelengths of 650, 672, and 713 nm on the Cary 60 UV–visible spectrophotometer. Before the measurement, the sample was saturated with red light (658 nm) to partially populate its Pfr state. All measurements were done in 20 mm Tris/HCl pH 8.0, 20 mm NaCl, 10% (v/v) glycerol, and 100 *μ*g mL^−1^ bovine serum albumin at temperatures of 15 °C, 22 °C, and 30 °C. The kinetic data were fitted to a double-exponential equation (2):


(2) $$F(t)\;=F_{0}+F_{1}\times \exp(-k_{1}\times t)+F_{2}\times \exp(-k_{2}\times t)\;$$

where *F_i_* are the absorbance amplitudes and *k_i_* represent the associated rate constants. The slower phase had the greater amplitude, and the corresponding rate constants were evaluated according to the Arrhenius (eq. (3)):


(3) $$k=A\times \exp(-E_{A}/RT)$$

where *A* is the pre-exponential factor, *E_A_* the activation energy, *R* the universal gas constant, and *T* the absolute temperature. Data fits were conducted with the Fit-o-mat software (Möglich 2018).

### Circular dichroism spectroscopy

Secondary-structure content and thermal stability of purified *At*PhyB PCM were assessed by circular dichroism (CD) spectroscopy on a JASCO J710 spectrophotometer equipped with a PTC-348WI Peltier element. CD spectra were recorded in a 1-mm cuvette on 2.6 *μ*m  *At*PhyB in buffer (20 mm NaCl, 50 mm Tris/HCl pH 8.0) at temperatures between 15 and 30 °C. The thermal denaturation was followed by the CD signal at a wavelength of 222 nm while increasing the temperature from 15 °C to 90 °C at a rate of 1 °C min^−1^. All samples were illuminated with a 733-nm LED before the measurements to populate the Pr state. The CD signal as a function of temperature was evaluated according to a 2-state unfolding model (eq. (4)):


(4) $$\Delta G\;=\;\Delta H\;-\;T\;/\;T_{m}\;\times \;\Delta H\;$$

where Δ*H*, Δ*G*, and *T*_m_ denote the enthalpy, the free enthalpy, and the midpoint, respectively, of unfolding. Data analysis was performed with the Fit-o-mat software (Möglich 2018).

### Steady-state fluorescence spectroscopy

Fluorescence analyses were performed on an Eclipse fluorescence spectrophotometer (Agilent Technologies) equipped with a Peltier thermostat. All experiments on PIF-mScarlet-I variants were done in buffer A (50 mm Tris/HCl pH 8.0, 20 mm NaCl). The fluorescence emission spectrum of P6A-mScarlet-I was recorded at 15 °C, at 20 nm protein concentration, an excitation wavelength at (525 ± 10) nm, and an emission slit width of 10 nm.

The binding of *At*PhyB PCM to P6A-mScarlet-I was monitored by the FRET from the mScarlet-I donor to the acceptor *At*PhyB PCM. To this end, 20 nm P6A-mScarlet-I were incubated at 15 °C with *At*PhyB PCM at concentrations between 0 and 2,000 nm. mScarlet-I fluorescence was recorded at excitation and emission wavelengths of (525 ± 10) nm and (590 ± 10) nm, respectively. Prior to the measurement, the samples were illuminated with red light (658 nm). All measurements were performed in buffer A supplemented with 100 *μ*g mL^−1^ bovine serum albumin. The fluorescence intensities were plotted against the total *At*PhyB PCM concentration and fitted to a single-site binding isotherm (eq. (5)).


(5) $$F(t)=F_{0}+(F_{1}-F_{0})\times [\mathrm{AtPhyB}]/([\mathrm{AtPhyB}]+K_{d}).$$

*F*_0_ and *F*_1_ are the fluorescence intensities of free and bound PIF-mScarlet-I, respectively, [AtPhyB] is the total concentration of the *At*PhyB PCM, and *K*_d_ is the dissociation constant. For the data acquired under red-light conditions, the *At*PhyB PCM concentrations were adjusted by a factor of 0.73 to account for the Pr/Pfr photostationary equilibrium of 0.27/0.73 attained upon illumination with 658-nm light, see above.

### Light-induced association and dissociation kinetics

We first determined the velocities of the light-driven Pr⇄Pfr interconversion at the same conditions later used for studying the PhyB:PIF association and dissociation kinetics. To this end, we monitored the weak Pr-state fluorescence of 1 *μ*m  *At*PhyB PCM in buffer A at 15 °C over time at excitation and emission wavelengths of ([650 ± 20] nm) and ([690 ± 10] nm), respectively. To follow the Pr→Pfr reaction, the samples were first illuminated with saturating 733-nm light, before being exposed to 658-nm light at intensities of 1, 10, 30, and 69 mW cm^−2^ for 20 s. For monitoring the Pfr→Pr conversion, the samples were pre-illuminated with saturating 658-nm light and then exposed to 42 mW cm^−2^ 733-nm light. The fluorescence over time, indicative of the Pr-state population, was fitted to single-exponential functions using Fit-o-mat (Möglich 2018) (eq. (6)).


(6) $$F(t)=F_{0}+F_{1}\times \exp(-k_{o}\times t)$$

where *F*_0_ and *F*_1_ are amplitude terms, and *k*_o_ is the observable rate constant.

For enhanced time resolution, we implemented an optical shutter (model SH05/M with controller SC10, Thorlabs, Bergkirchen, Germany) that controlled the exposure of the sample to the light source with a temporal precision of better than 10 ms. Using the shutter setup, 1 *μ*m  *At*PhyB PCM was illuminated at 15 °C for between 0.1 and 1.0 s with 658-nm light at 69 mW cm^−2^ intensity, and fluorescence was recorded as before. The speed of the Pr→Pfr conversion was evaluated by evaluating the decay of the Pr-state fluorescence as a function of the shutter-opening time according to a single-exponential function, see eq. (1). Based on these measurements, an opening time of 0.5 s was used subsequently.

The measurements of the association kinetics were performed in buffer A at temperatures between 15 and 30 °C. In initial experiments under prolonged illumination (see Fig. 2B and C, Supplementary Fig. S5), 20 nm P6A-mScarlet-I were incubated with 1 *μ*m  *At*PhyB PCM or 2 *μ*m AtPhyB (1-982). Prior to the measurements, the samples were exposed to saturating 733-nm light. At the start of the experiment, the samples were irradiated with 658-nm light at intensities of 1, 10, 30, and 69 mW cm^−2^ for 20 s, and the mScarlet-I fluorescence was recorded at excitation and emission wavelengths of (565 ± 20) nm and (600 ± 20) nm. The initial decay of the fluorescence was evaluated by single-exponential functions (eq. (6)) using Fit-o-mat (Möglich 2018).

For subsequent experiments with shutter control (see Fig. 3A, Supplementary Fig. S8), 20 nm of a given PIF-mScarlet-I variant was incubated with *At*PhyB PCM at concentrations between 500 and 2,000 nm. mScarlet-I fluorescence was recorded over time at excitation and emission wavelengths of (565 ± 20) nm and (600 ± 20) nm. For the measurement of the red-light-induced association kinetics, the samples were first illuminated with saturating 733-nm light, before being exposed to 658-nm light for 0.5 s as controlled by the optical shutter, see above. The resultant fluorescence kinetics over time were evaluated according to pseudo-first-order kinetics, i.e. single-exponential functions, see eq. (6). The interaction of 30 nm P6A-mScarlet-I with *At*PhyB (1-982) was studied likewise at phytochrome concentrations between 750 and 2,500 nm. For the dissociation kinetics, the samples were first exposed to saturating 658-nm light and then exposed to continuous far-red light during the entire time course. The resultant dissociation kinetics under far-red light were fitted to a consecutive kinetic model (Golonka et al. 2020) according to eq. (7).


(7) $$\begin{array}{rl} F(t)\;= & F_{0}+(F_{1}-F_{0})\times k_{q}k_{o}/(k_{q}-k_{o})\\ & \times [-1/k_{q}\times \exp(-k_{q}\times t)+1/k_{o}\times \exp(-k_{o}\times t)] \end{array}$$

where *F*_0_ and *F*_1_ are fluorescence amplitudes, *k*_q_ is the rate constant for the initial *At*PhyB PCM Pfr→Pr photoconversion reaction, and *k*_o_ is the rate constant for the subsequent dissociation reaction. The rate constant of photoconversion *k*_q_ was fixed at values of 1.94 and 2.75 s^−1^ for *At*PhyB PCM and *At*PhyB (1-982), respectively, as determined by the above fluorescence measurements.

For both the data acquired upon red-light and far-red-light exposure, the observable rate constants *k*_o_ were evaluated as a function of the total *At*PhyB PCM or *At*PhyB (1-982) concentration according to eq. (8) to determine the bimolecular rate constants *k*_a_ for the association and the unimolecular rate constants *k*_d_ for the dissociation of the PIF:*At*PhyB PCM complex (see Fig. 3B to F, Supplementary Figs. S7 and S9 to S12).


(8) $$k_{o}=k_{a}\times [At\mathrm{PhyB}]+k_{d}.$$

To account for the fractional Pfr population of 73% under red light (see above), the bimolecular rate constant *k*_a_ was divided by a factor of 0.73. In the case of *At*PhyB (1-982), the rate constants *k*_a_ were corrected for the relevant Pfr:Pr ratio at photostationary state determined by UV–vis absorbance measurements (see Supplementary Fig. S3E). For the data acquired under far-red light, the bimolecular rate constant *k*_aR_ was fixed at 0. The temperature dependence of the rate constants *k*_aFR_ and *k*_dFR_ recorded upon red-light exposure was further evaluated according to the Arrhenius equation (eq. (3)) (see Fig. 4 and Supplementary Fig. S13).

### Fluorescence-correlation spectroscopy

The temperature-dependent diffusion of the various PIF variants, *At*PhyB PCM, and *At*PhyB (1-982) was measured on a home-built epi-fluorescence confocal microscope (see Fig. 5 and Supplementary Fig. S14). The microscope was equipped with a water-immersion objective (UPLSAPO 60x, NA 1.2, Olympus) and an avalanche photodiode (PD5C0C, Micro Photon Devices) for single-photon detection. The detection pinhole had a diameter of 100 *μ*m. A 100-*μ*L droplet of the sample was placed in a chamber of a multi-well coverslip (μ Slide 18 Well, ibidi GmbH) with a glass bottom. For excitation of mScarlet-I, covalently linked to the PIF protein, we used a 510-nm pulsed diode laser (BDS-SM 510, Becker & Hickl) running at 20 MHz repetition frequency. For excitation of *At*PhyB variants, we used a 640-nm cw laser diode (λ mini EVO 640-75, RBG Photonics). Fluorescence emission of mScarlet-I and *At*PhyB was detected through long-pass filters with cutoffs of 526 nm (BLP01-514R Edge Basic, Semrock) and 659 nm (647 LP edge basic, AHF Analysentechnik), respectively. The microscope was calibrated with a Rhodamine 110 solution excited at 510 nm and an Atto-655 (Atto-655 carboxy derivative, ATTO-TEC GmbH) solution excited at 640 nm, yielding lateral radii *ω*_r_ of (250 ± 22) nm and (304 ± 7) nm, respectively, of the detection volume. For the measurements on *At*PhyB (1-982), the latter radius amounted to (313 ± 3) nm.

For the experiment with cw excitation, the autocorrelation functions were measured with a TCSPC module (SPC-130, Becker & Hickl GmbH). After-pulsing of the avalanche photodiode was taken into account by correcting the recorded autocorrelation function $G^{\prime }(\tau )$ according to:


(9) $$G(\tau )=G^{\prime }(\tau )-\frac{\left\langle i_{ap}\right\rangle }{\left\langle i\right\rangle }\times (G_{ap}(\tau )-1)$$

where *τ*_D_ is the lag-time and $G_{ap}(\tau )\;$ is the after-pulsing function of the avalanche photodiode (Zhao et al. 2003). $G_{ap}(\tau )$ was recorded with the uncorrelated emission of an LED torch light. The values *i* and $i_{ap}\;$ are the mean count rates of the actual experiment and the measurement of $G_{ap}(\tau )$, respectively.

For the measurement with pulsed excitation, the after-pulsing effects were removed by applying fluorescence lifetime correlation spectroscopy (FLCS) (Enderlein and Gregor 2005; Kapusta et al. 2007). For that purpose, we recorded the micro-times of the photons with the same TCSPC module, and the autocorrelation was subsequently calculated from these data with the FLCS method. The corrected autocorrelation functions were then evaluated by fitting them to the standard 3-dimensional diffusion model:


(10) $$G(\tau )=G_{0}\times {\left(1+\frac{\tau }{\tau_{D}}\right)}^{-1}\times {\left(1+\frac{\tau }{\gamma^{2}\tau_{D}}\right)}^{-1/2}$$

where *τ* is the correlation time, $G_{0}\;$ is the correlation amplitude, and *γ* is the axial ratio of the detection volume. The *γ* value was determined by measuring the autocorrelation function of a Rhodamine 110 solution excited at 510 nm and an Atto-655 solution excited at 640 nm. With *γ* as a free parameter, the autocorrelation functions were then fitted to eq. (10) which yielded values of *γ* = 9 for 510 nm and *γ* = 6 for 640 nm. All calculations were done with custom Python scripts. To confirm the accuracy of this approach, we compared the results of our FLCS algorithm with those obtained with commercial software. To this end, we measured the autocorrelation function of Rhodamine B in water with a commercial confocal microscope (MT200, PicoQuant) and analyzed it with the included SymPhoTime software package. The same data set was then analyzed with the custom Python script. The comparison showed that both results were consistent (Supplementary Fig. S16).

The fluorescence of 20 nm of a given PIF-mScarlet-I variant, 200 nm  *At*PhyB PCM, or 200 nm  *At*PhyB (1-982) was recorded in buffer A. Prior to the measurements of *At*PhyB fluorescence, the sample was illuminated with a 733-nm LED (42 mW cm^−2^). All samples were measured at least 3 times over 3 min at different temperatures from 15 °C to 30 °C. Measurements were done at a laser intensity of 3 *μ*W, measured at the entrance of the microscope. For temperature control, we used a self-made cooling/heating element that allowed adjusting the sample temperature from 16 to 40 °C (Supplementary Fig. S17). The element was machined out of a copper block to fit onto the multi-well coverslip. Through internal water channels, the copper block was connected to a lab thermostat (Julabo F32) and thereby adjusted to the desired value. The multi-well coverslip together with the copper block was placed on an aluminum sheet with a central aperture that was attached to the microscope stage. A 3D-printed plastic spacer between the stage and the aluminum sheet provided thermal insulation, as did a styrofoam cover. During the measurements, the temperature was monitored with a Pt1000 temperature sensor (NB-PTCO-050, TE Connectivity Sensors) directly immersed into the sample.

The transversal diffusion coefficients *D* for the PIF-mScarlet-I variants, *At*PhyB PCM, and *At*PhyB (1-982), determined by FCS at temperatures from 15 °C to 30 °C, were evaluated according to the Stokes–Einstein equation (see Fig. 5B and C and Supplementary Fig. S14):


(11) $$D=k_{b}T/(6\pi \eta R_{h})$$

where *k*_b_ signifies the Boltzmann constant, *T* is the absolute temperature, *η* is the viscosity, and *R*_h_ is the hydrodynamic radius. The viscosities at the different temperatures were assumed to equal those of water (Weast 1974). The diffusional encounter rates *k*_bi_ were calculated according to the von Smoluchowski equation (Smoluchowski 1918):


(12) $$D=4\pi N_{A}\sum R_{h}\sum D\times 1000$$

where the *R*_h_ and *D* are the hydrodynamic radii and diffusion coefficients, respectively, of a given PIF-mScarlet-I variant, *At*PhyB PCM, and *At*PhyB (1-982), and *N*_A_ is the Avogadro constant.

### Reporter-gene assays in mammalian cell culture

The split-transcription factor constructs of the *At*PhyB PCM, full-length *At*PhyB, and the PIF variants were prepared as before (Golonka et al. 2019). To this end, a SEAP (secreted alkaline phosphatase) reporter was placed under control of a synthetic inducible promoter comprising the cognate binding sequence of the E protein (etr_8_) and a minimal CMV promoter (P_min_) (Supplementary Tables S3 and S4). Furthermore, the *Gaussia* luciferase was put under control of a constitutive promoter and placed onto the same plasmid. Thereby, SEAP activity levels can be normalized to the *Gaussia* luciferase signal to correct for variations of cell density, transfection efficiency, and overall expression.

To assess the red-light-inducible reporter expression, the PhyB-VP16/E-PIF vector and the SEAP reporter plasmid were transfected into CHO-K1 cells (DSMZ, Braunschweig, Germany) using polyethyleneimine (PEI; Polysciences Inc. Europe, Hirschberg, Germany; no. 23966–1). All plasmids were transfected in equal amounts (w/w). Constitutively expressed E-VP16 served as a positive control, yielding the maximum reporter expression irrespective of illumination. As a negative control, the reporter construct alone was transfected. CHO-K1 cells were cultivated in HAM's F12 medium (PAN Biotech, Aidenbach, Germany; no. P04–14500) as before (Golonka et al. 2019). After 16 h incubation in the dark, the cells were supplemented with 15 *μ*m phycocyanobilin (stock solution in DMSO; Si-chem, Logan, UT, USA; SC-1800). Cells were then incubated for 24 h in darkness, under 20 *μ*E m^−2^ s^−1^ of 740-nm light, or at different intensities (1 to 100 *μ*E m^−2^ s^−1^) of 660-nm light. SEAP activity and *Gaussia* luciferase assays were performed and analyzed as before (Golonka et al. 2019). Reporter-gene experiments for full-length *At*PhyB connected to VP16 were conducted likewise. Reporter-gene signals at the different light conditions were compared to the maximum signal by 1-way ANOVA.

### Reporter-gene assays in Arabidopsis protoplasts

Arabidopsis protoplast isolation and transformation were done as before (Ochoa-Fernandez et al. 2016; Ochoa-Fernandez et al. 2020). Protoplasts were isolated from 2-wk-old Arabidopsis plantlet leaves grown on 12-cm square plates containing SCA medium (0.32% [w/v] Gamborg's B5 basal salt powder with vitamins, 4 mm MgSO_4_·7H_2_O, 43.8 mm sucrose and 0.8% [w/v] phytoagar in H_2_O, pH 5.8, 0.1% [v/v] Gamborg's B5 Vitamin Mix [bioWORLD]) in a 23 °C plant chamber with 16 h light/8 h dark regime. The protoplasts were isolated by a floatation method with MMM solution (15 mm MgCl_2_, 5 mm MES, 467 mm mannitol, pH 5.8). The protoplasts were collected at the interphase and transferred to a W5 solution (2 mm MES, 154 mm NaCl, 125 mm CaCl_2_·2H_2_O, 5 mm KCl, 5 mm glucose, pH 5.8) prior to transformation. A final amount of 30 *μ*g DNA mixtures of the PhyB, PIF and reporter plasmids in 1:1:1 (v:v:v) ratio, and 3 *μ*g DNA of the constitutive RLuc as normalization were used to transform 500,000 protoplasts by polyethylene-glycol (PEG4000). As a positive control, a plasmid constitutively expressing E-VP16 was co-transformed with the reporter plasmid. The reporter construct alone served as a negative control. Four transformations of each setup were done in parallel and pooled together afterwards. Protoplasts were then distributed into 24-well plates in 600-*μ*L aliquots (ca. 200,000 protoplasts, sufficient for measuring 3 replicates for both FLuc and RLuc). Afterwards, the plates were either illuminated with varying intensities of 660-nm light or 10 *μ*E m^−2^ s^−1^ of 740-nm light, or kept in darkness for 18 to 20 h at room temperature. Firefly (FLuc) and *Renilla* luciferase (RLuc) activities were determined as described before (Ochoa-Fernandez et al. 2020). Reporter-gene signals at the different light conditions were compared to the maximum signal by 1-way ANOVA.

### Modeling of interaction dynamics and equilibria under illumination

The Pr⇄Pfr photoconversion dynamics of *At*PhyB and its light-dependent interactions with the PIF partner protein were cast as a system of ODE according to Fig. 6A.


(13) $$d[R]/dt=-k_{p}[R]-k_{\mathrm{aR}}[R][P]+k_{q}[FR]+k_{\mathrm{dR}}[RP]$$

(14) $$d[FR]/dt=-k_{q}[FR]-k_{\mathrm{aFR}}[FR][P]+k_{p}[R]+k_{\mathrm{dFR}}[FRP]$$

(15) $$d[RP]/dt=-k_{p}[RP]-k_{\mathrm{dR}}[RP]+k_{q}[FRP]+k_{\mathrm{aR}}[R][P]$$

(16) $$d[FRP]/dt=-k_{q}[FRP]-k_{\mathrm{dFR}}[FRP]+k_{p}[RP]+k_{\mathrm{aFR}}[FR][P]$$

(17) $$d[P]/dt=-k_{\mathrm{aR}}[R][P]-k_{\mathrm{aFR}}[FR][P]+k_{\mathrm{dR}}[RP]+k_{\mathrm{dFR}}[FRP].$$

In equations (13–17), [*R*] and [*FR*] denote the concentrations of *At*PhyB in its Pr and Pfr states, [*RP*] and [*FRP*] the concentrations of the Pr and Pfr forms in complex with the PIF protein, and [*P*] that of free PIF. The microscopic rate constants *k*_p_ and *k*_q_ describe the unimolecular photoconversion in the Pr→Pfr and Pfr→Pr directions, respectively, which are assumed to be independent of whether *At*PhyB is in complex with PIF or not. Finally, *k*_a_ and *k*_d_ are the bimolecular association and unimolecular dissociation rate constants for *At*PhyB:PIF complex formation, with the subscripts R and FR denoting the Pr and Pfr states, respectively. The association rate constant within the Pr state, *k*_aR_, was constrained at zero. For times prior to the onset of illumination at time *t*_0_ and after illumination ceases at *t*_1_, the photoconversion rate constants *k*_p_ and *k*_q_ were set to zero. The ODE system was numerically solved via the *integrate* module which is part of the Python library *scipy* (Virtanen et al. 2020). Using Fit-o-mat (Möglich 2018), the experimental data recorded at illumination intensities of 1, 10, 30, and 69 mW cm^−2^ were globally evaluated by Nelder–Mead minimization and nonlinear least-squares fitting.

Under continuous illumination, the reaction network, defined by equations (13–17), assumes a photostationary state. Using boundary conditions for mass conservation of *At*PhyB and PIF, -equations (18–19), respectively, the species concentrations at photostationary state can be calculated.


(18) $$R_{0}=[R]+[FR]+[RP]+[FRP]$$

(19) $$P_{0}=[P]+[RP]+[FRP].$$

The concentration of unbound PIF [*P*] at photostationary state can be obtained as the real third root of the third-order polynomial given in equation (20).


(20) $$\begin{array}{rl} 0\;= & -k_{3}k_{12}k_{5}[P{]}^{3}+[P{]}^{2}(-gk_{26}k_{5}-gk_{1}k_{5}+gk_{6}k_{5}-k_{3}k_{126}k_{12}\\ & -(k_{3}-k_{5})k_{26}k_{12}-k_{3}k_{12}k_{5}R_{0}+k_{3}k_{12}k_{5}P_{0}-k_{6}k_{12}k_{5})\\ & +[P](gk_{26}k_{5}P_{0}-gk_{26}k_{12}-gk_{1}k_{5}R_{0}-gP_{0}(k_{6}-k_{1})k_{5}\\ & -(k_{3}-k_{5})k_{26}k_{126}k_{12}/k_{5}-k_{3}k_{126}k_{12}R_{0}+k_{3}k_{126}k_{12}P_{0}-k_{6}k_{126}k_{12}\\ & +(k_{3}-k_{5})k_{26}P_{0}k_{12}+k_{6}k_{12}k_{5}P_{0})+gk_{26}k_{12}P_{0}\\ & +(k_{3}-k_{5})k_{26}k_{126}k_{12}P_{0}/k_{5}+k_{6}k_{126}k_{12}P_{0}. \end{array}$$

For simplicity, the microscopic rate constants *k*_p_, *k*_q_, *k*_aR_, *k*_dR_, *k*_aFR_, and *k*_dFR_ have been replaced by the rate constants *k*_1_ through *k*_6_. The rate constants *k*_12_, *k*_26_, and *k*_126_ refer to the sum of the individual rate constants specified in the subscript, e.g.:


(21) $$k_{126}=k_{1}+k_{2}+k_{6}.$$

The variable *g* is defined as:


(22) $$g=-k_{126}(k_{3}-k_{5})/k_{5}+k_{4}-k_{6}.$$

The third-order polynomial can be solved for [*P*] by Cardano's formula (Braunstein et al. 2001). With the knowledge of [*P*] at photostationary state, the remaining species concentrations can be calculated according to equations (23–24) and (18–19).


(23) $$\begin{array}{rl} {}[RP]= & \;-(-k_{26}k_{12}P_{0}/k_{5}[P]-k_{26}P_{0}+k_{26}k_{12}/k_{5}+k_{12}[P]+k_{1}R_{0}\\ & +P_{0}(k_{6}-k_{1}))/(k_{126}k_{12}/k_{5}[P]+k_{12}) \end{array}$$

(24) $$[FR]=(-k_{1}[RP]+k_{26}(R_{0}-[P]-[RP]))/k_{5}[P].$$

The analytical solution at photostationary state was validated against the numerical solution of the above ODE system, see eqs. (13–17). All calculations and simulations were carried out with Fit-o-mat (Möglich 2018).

### Accession numbers

Sequence data from this article can be found in the GenBank/EMBL data libraries under accession numbers CAA35222.1 (*At*PhyB), AAC33213.1 (PIF3), BAC10690.1 (PIF6), and APD76536 (mScarlet-I).

## Supplementary Material

koae249_Supplementary_Data

## Data Availability

The data underlying this article will be shared on reasonable request to the corresponding author.

## Supplementary data

The following materials are available in the online version of this article.

**Supplementary Figure S1.** Absorbance spectra and dark-recovery kinetics of the AtPhyB PCM, and emission spectra of light-emitting diodes and mScarlet-I.

**Supplementary Figure S2.** Binding equilibrium between *At*PhyB and P6A-mScarlet-I at 15 °C.

**Supplementary Figure S3.** Influence of red-light power and temperature on the Pr⇄Pfr photoconversion of *At*PhyB PCM and *At*PhyB982.

**Supplementary Figure S4.**  *At*PhyB PCM Pr⇄Pfr photoconversion kinetics and implementation of an optical shutter.

**Supplementary Figure S5.** Light-dependent *At*PhyB:PIF interaction dynamics.

**Supplementary Figure S6.** Light-inducible transgene expression in mammalian cells and Arabidopsis protoplasts.

**Supplementary Figure S7.** Bimolecular association and unimolecular dissociation kinetics of the *At*PhyB:P6A complex upon red-light activation or under far-red light.

**Supplementary Figure S8.** Bimolecular association and unimolecular dissociation kinetics of the *At*PhyB:PIF complex.

**Supplementary Figure S9.** Bimolecular association and unimolecular dissociation kinetics of the *At*PhyB:PIF6 complex upon red-light activation or under far-red light.

**Supplementary Figure S10.** Bimolecular association and unimolecular dissociation kinetics of the *At*PhyB:P3A complex upon red-light activation or under far-red light.

**Supplementary Figure S11.** Bimolecular association and unimolecular dissociation kinetics of the *At*PhyB:PIF3 complex upon red-light activation or under far-red light.

**Supplementary Figure S12.** Bimolecular association and unimolecular dissociation kinetics of the *At*PhyB982:P6A complex upon red-light activation or under far-red light.

**Supplementary Figure S13.** Temperature dependence of the *At*PhyB (1-982):P6A interaction dynamics.

**Supplementary Figure S14.** Hydrodynamics of *At*PhyB and PIF variants at different temperatures.

**Supplementary Figure S15.** Analysis of *At*PhyB thermal stability by circular dichroism.

**Supplementary Figure S16.** Benchmarking of custom Python script for fluorescence lifetime correlation spectroscopy.

**Supplementary Figure S17.** Customized cooling/heating device for temperature-dependent diffusion measurements.

**Supplementary Table S1.** Hydrodynamic properties of the *At*PhyB, PIF3, and PIF6 variants.

**Supplementary Table S2.** Parameters used in the analysis of the attenuation effect.

**Supplementary Table S3.** Oligonucleotide primers used in the study.

**Supplementary Table S4.** Identity of the protein constructs used in the study.

## Dive Curated Terms

The following phenotypic, genotypic, and functional terms are of significance to the work described in this paper:

pif7 Gramene: AT5G61270
pif7 Araport: AT5G61270

PIF4 Gramene: AT2G43010
PIF4 Araport: AT2G43010

ELF3 Gramene: AT2G25930
ELF3 Araport: AT2G25930

PIF3 Gramene: AT1G09530
PIF3 Araport: AT1G09530

heme CHEBI: CHEBI:30413
