# Supplementary material for: Plant phytochrome interactions decode light and temperature signals
Source: Plant Cell. 2024 Sep 11;36(12):4819–39. doi: 10.1093/plcell/koae249 (PMC11638003; doi:10.1093/plcell/koae249)
Supplement: koae249_Supplementary_Data [file koae249_supplementary_data.zip › PNASNEXUS-PNASNEXUS-2024-00713-TRR-s01.pdf]

Supplementary Information

Plant Phytochrome Interactions Decode Light and Temperature Signals

Chengwei Yi[1,‡], Uwe Gerken[2,‡], Kun Tang[3,‡], Michael Philipp[2,‡],

Matias D. Zurbriggen[3,4‡], Jürgen Köhler[2,5,6,‡], Andreas Möglich[1,7,8,*,‡,†]

[1] Department of Biochemistry, University of Bayreuth, 95447 Bayreuth, Germany.
[2] Lehrstuhl für Spektroskopie weicher Materie, Universität Bayreuth, 95447 Bayreuth, Germany.
[3] Institute of Synthetic Biology, Heinrich Heine University Düsseldorf, 40225 Düsseldorf, Germany.
[4] CEPLAS - Cluster of Excellence on Plant Sciences, Heinrich Heine University Düsseldorf, 40225 Düsseldorf, Germany
[5] Bayerisches Polymer Institut, Universität Bayreuth, 95447 Bayreuth, Germany.
[6] Bayreuther Institut für Makromolekülforschung, 95447 Bayreuth, Germany.
[7] Bayreuth Center for Biochemistry & Molecular Biology, Universität Bayreuth, 95447 Bayreuth, Germany.
[8] North-Bavarian NMR Center, Universität Bayreuth, 95447 Bayreuth, Germany.

* Corresponding author. Email: andreas.moeglich@uni-bayreuth.de
‡ ORCID identifiers: C.Y. 0000-0002-2167-4536; U.G. 0000-0002-6447-2803; K.T. 0000-0002-4796-9869; M.P. 0009-0007-8717-7317; M.D.Z. 0000-0002-3523-2907; J.K. 0000-0002-4214-4008; A.M. 0000-0002-7382-2772
† The author responsible for distribution of materials integral to the findings presented in this article in accordance with the policy described in the Instructions for Authors (https://academic.oup.com/plcell/pages/General-Instructions) is Andreas Möglich (andreas.moeglich@uni-bayreuth.de).

Table of Contents

Supplementary Figure S1 ____ 3

Supplementary Figure S2 ____ 4

Supplementary Figure S3 ____ 5

Supplementary Figure S4 ____ 7

Supplementary Figure S5 ____ 9

Supplementary Figure S6 ____ 11

Supplementary Figure S7 ____ 13

Supplementary Figure S8 ____ 14

Supplementary Figure S9 ____ 15

Supplementary Figure S10 ____ 16

Supplementary Figure S11 ____ 17

Supplementary Figure S12 ____ 18

Supplementary Figure S13 ____ 19

Supplementary Figure S14 ____ 20

Supplementary Figure S15 ____ 21

Supplementary Figure S16 ____ 22

Supplementary Figure S17 ____ 23

Supplementary Table S1 – Hydrodynamic properties of the *At*PhyB, PIF3, and PIF6 variants. ____ 24

Supplementary Table S2 – Parameters used in the analysis of the attenuation effect. ____ 25

Supplementary Table S3 – Oligonucleotide primers used in the study. ____ 26

Supplementary Table S4 – Identity of the protein constructs used in the study. ____ 27

References ____ 29

Supplementary Figure S1

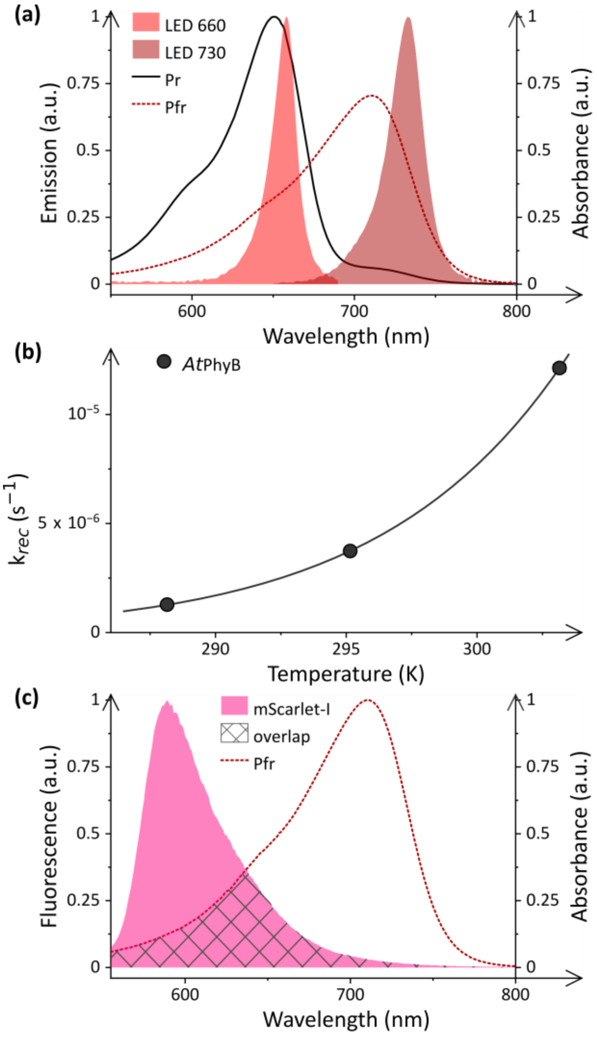

Supports Fig. 1. Absorbance spectra and dark-recovery kinetics of the *At*PhyB PCM, and emission spectra of light-emitting diodes and mScarlet-I. **a**, Normalized absorbance spectra of *At*PhyB in its Pr and Pfr states (solid black and dashed brown lines). Emission spectra of the 658-nm and 733-nm LEDs used throughout the study (red and brown shaded areas). **b**, Kinetics of Pfr→Pr recovery of the *At*PhyB PCM at 15°C, 22°C, and 30°C. The line denotes an Arrhenius fit and yields a preexponential factor of (8.3 ± 1.1) × 10^{13} s^{-1} and an activation energy of (1.09 ± 0.02) × 10^5 J mol^{-1}. **c**, Normalized absorbance spectrum of *At*PhyB in its Pfr state (dashed brown line) and normalized mScarlet-I emission spectrum (magenta shading). The region of spectral overlap is highlighted by the checkered fill.

Supplementary Figure S2

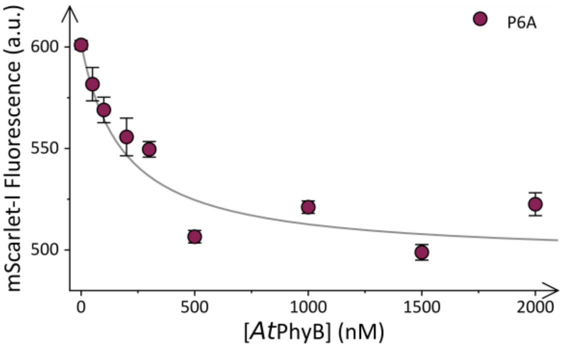

Supports Figs. 1 and 2. Binding equilibrium between *At*PhyB and P6A-mScarlet-I at 15°C. The fluorescence intensity of mScarlet-I was measured at different concentrations of red-light-exposed *At*PhyB (658 nm). Data represent mean ± s.d. of three independent measurements. The line represents a fit to a single-site binding isotherm. The *x* axis denotes the total *At*PhyB concentration, and the dissociation constant, K_d, determined by data fitting was adjusted for the fractional Pfr population of around 73% upon exposure to red light (see Fig. 1b).

Supplementary Figure S3

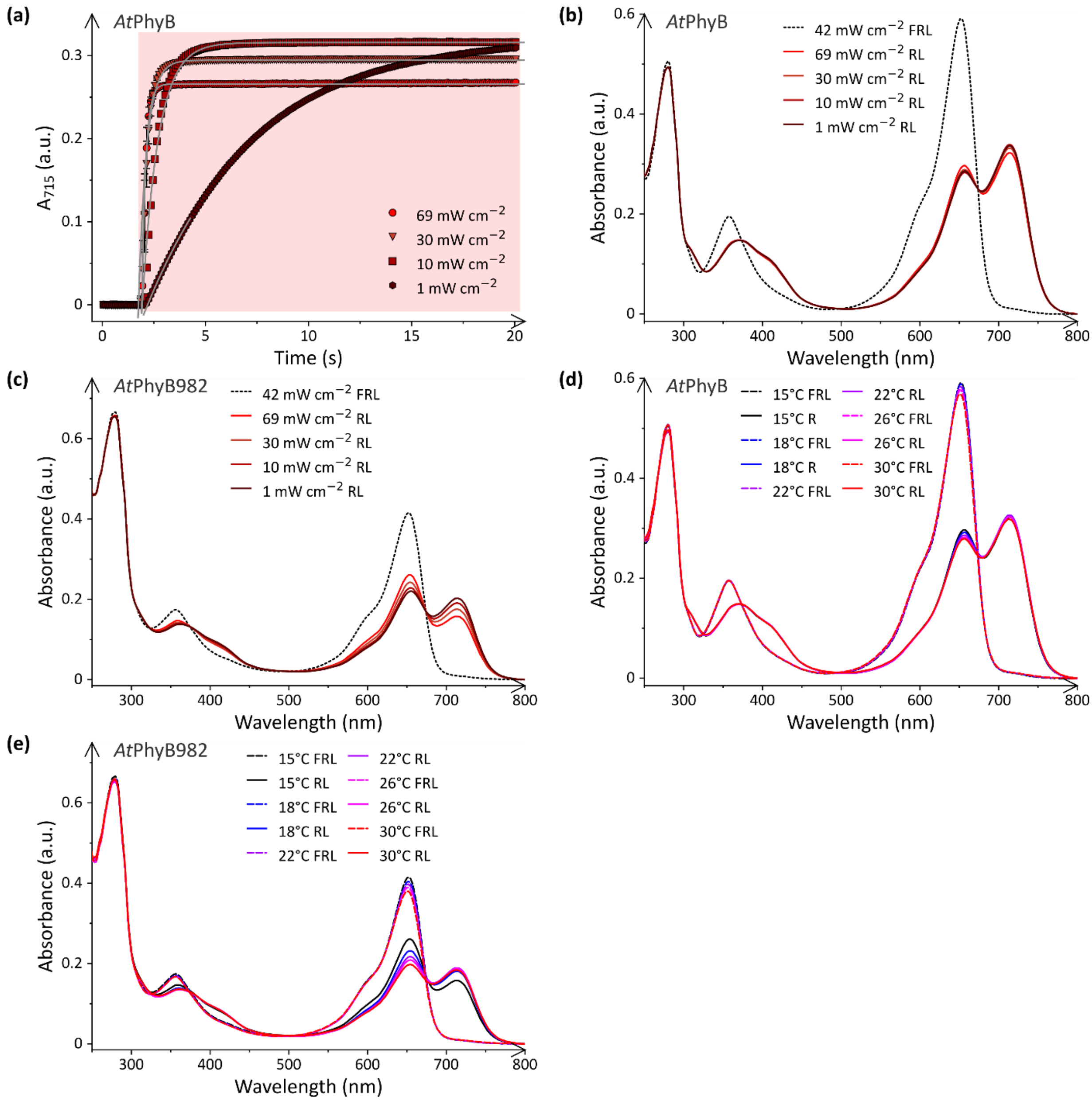

Supports Fig. 2. Influence of red-light power and temperature on the Pr⇄Pfr photoconversion of *At*PhyB PCM and *At*PhyB982. **a**, Pr→Pfr photoconversion of the *At*PhyB PCM monitored by absorbance at 715 nm upon illumination with red-light powers of 1 mW cm^{-2} (brown hexagons), 10 mW cm^{-2} (reddish-brown squares), 30 mW cm^{-2} (dark red triangles), and 69 mW cm^{-2} (red circles). Light exposure began at around 2 s after the start of the recording (red shading). The grey lines denote fits to single-exponential functions. The differences in the asymptotic absorbance values for the individual traces owe to scattering of the red red light used for driving the photoconversion. **b**, Absorbance spectra of the *At*PhyB PCM at 15°C following saturating exposure to far-red light (FRL, dashed line, 733 nm, 42 mW cm^{-2}) or red light (RL) at intensities of 1 mW cm^{-2} (brown line), 10 mW cm^{-2} (reddish-brown line), 30 mW cm^{-2} (dark red line), and 69 mW cm^{-2} (red line). **c**, As panel b but

for *At*PhyB982. **d**, Absorbance spectra for the *At*PhyB PCM following saturating with 42 mW cm^{-2} 733-nm light (FRL, dashed curves) or 69 mW cm^{-2} 658-nm light (RL, solid curves) at temperatures of 15°C (black lines), 18°C (blue lines), 22°C (purple lines), 26°C (pink lines), and 30°C (red lines). **e**, As panel d but for *At*PhyB982. In panels b-e, the illumination times for red light and far-red light were 5 minutes and 2 minutes, respectively.

Supplementary Figure S4

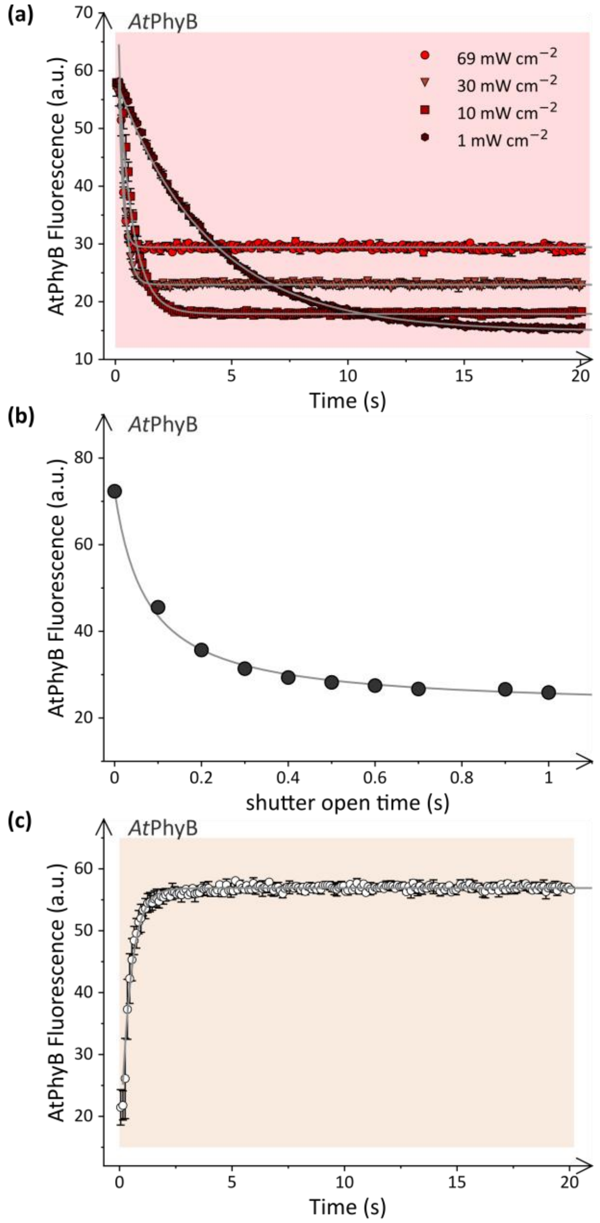

Supports Fig. 2. *At*PhyB PCM Pr⇄Pfr photoconversion kinetics and implementation of an optical shutter. **a**, 1,000 nM *At*PhyB were incubated at 15°C, and the Pr-state fluorescence was measured at 650 nm excitation and 690 nm emission. During the measurement, the samples were exposed to 658-nm light of different intensity [marked by red shading; from bottom to top 1 mW cm^{-2} (dark brown symbols), 10 mW cm^{-2} (medium brown), 30 mW cm^{-2} (light brown), and 69 mW cm^{-2} (red)]. Notably, the fluorescence plateau after 20 s scales with the light intensity because the red light used for driving the Pr→Pfr photoconversion by necessity also excites the Pr fluorescence in parallel. The grey lines represent fits to single-exponential functions. **b**, Using an optical shutter, 1,000 nM *At*PhyB PCM were exposed to defined pulses of 658-nm light (at 69 mW cm^{-2} intensity) at 15°C. The decay of the resultant Pr-state fluorescence with shutter-opening time was fitted to a single-

exponential function (grey line). **c**, As in panel a, but *At*PhyB was preconditioned by saturating illumination with 658-nm light prior to the experiment. Then, the Pfr→Pr reversion kinetics under 42 mW cm^{-2} 733-nm light (brown shading) were monitored by the Pr-state fluorescence and fitted to a single exponential. Data in panels a-c represent mean ± s.d. of three independent measurements.

Supplementary Figure S5

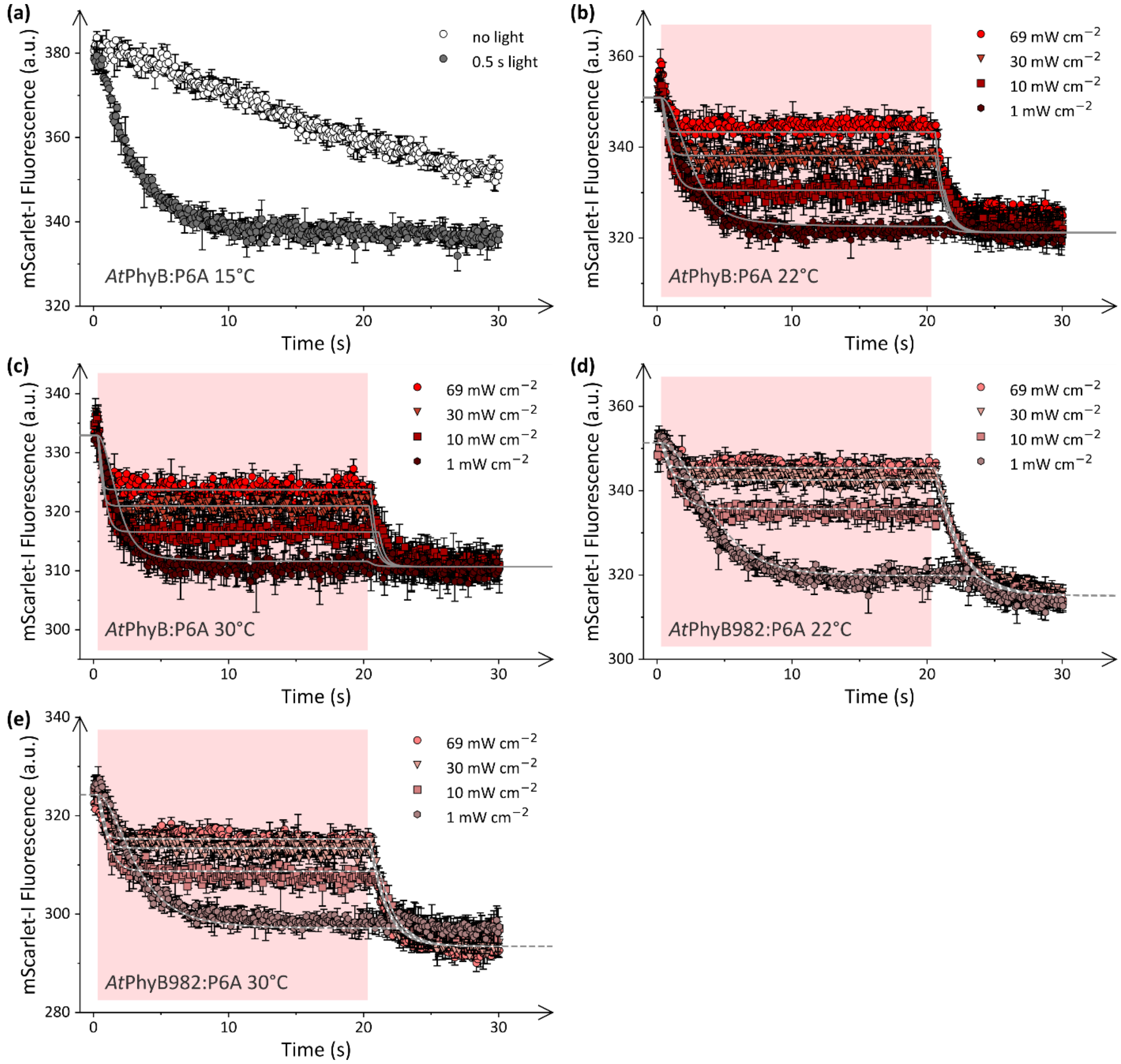

Supports Fig. 2. Light-dependent *At*PhyB:PIF interaction dynamics. **a**, 20 nM P6A-mScarlet-I and 1,000 nM *At*PhyB were incubated at 15°C, and the mScarlet-I fluorescence was measured at (565 ± 20) nm excitation and (600 ± 20) nm emission. The grey dots denote an experiment in which the samples were illuminated with 658-nm light at 69 mW cm^{-2} intensity for 0.5 s at the start of the experiment. The white symbols are for a sample not illuminated with 658-nm light. The slow decrease of the mScarlet-I fluorescence signal reflects the gradual Pr→Pfr photoconversion triggered by the probe light of the fluorescence spectrophotometer. **b**, 20 nM P6A-mScarlet-I and 1,000 nM *At*PhyB were incubated at 22°C, and the mScarlet-I fluorescence was measured at (565 ± 20) nm excitation and (600 ± 20) nm emission. During the initial 20 s of the experiment, the samples were illuminated with 658-nm light at different intensities [from bottom to top 1 mW cm^{-2} (dark brown symbols), 10 mW cm^{-2} (medium brown), 30 mW cm^{-2} (light brown), and 69 mW cm^{-2} (red)]. The solid lines represent a global fit of the data to the numeric solution of the reaction scheme in

Fig. 2a. **c**, As in panel b but at a temperature of 30°C. **d**, As in panel b but for 20 nM P6A-mScarlet-I mixed with 2,000 nM *At*PhyB (1-982). The global fit is shown as the set of dashed lines. **e**, As in panel d but at a temperature of 30°C. Data in panels a-e represent mean ± s.d. of three independent experiments.

Supplementary Figure S6

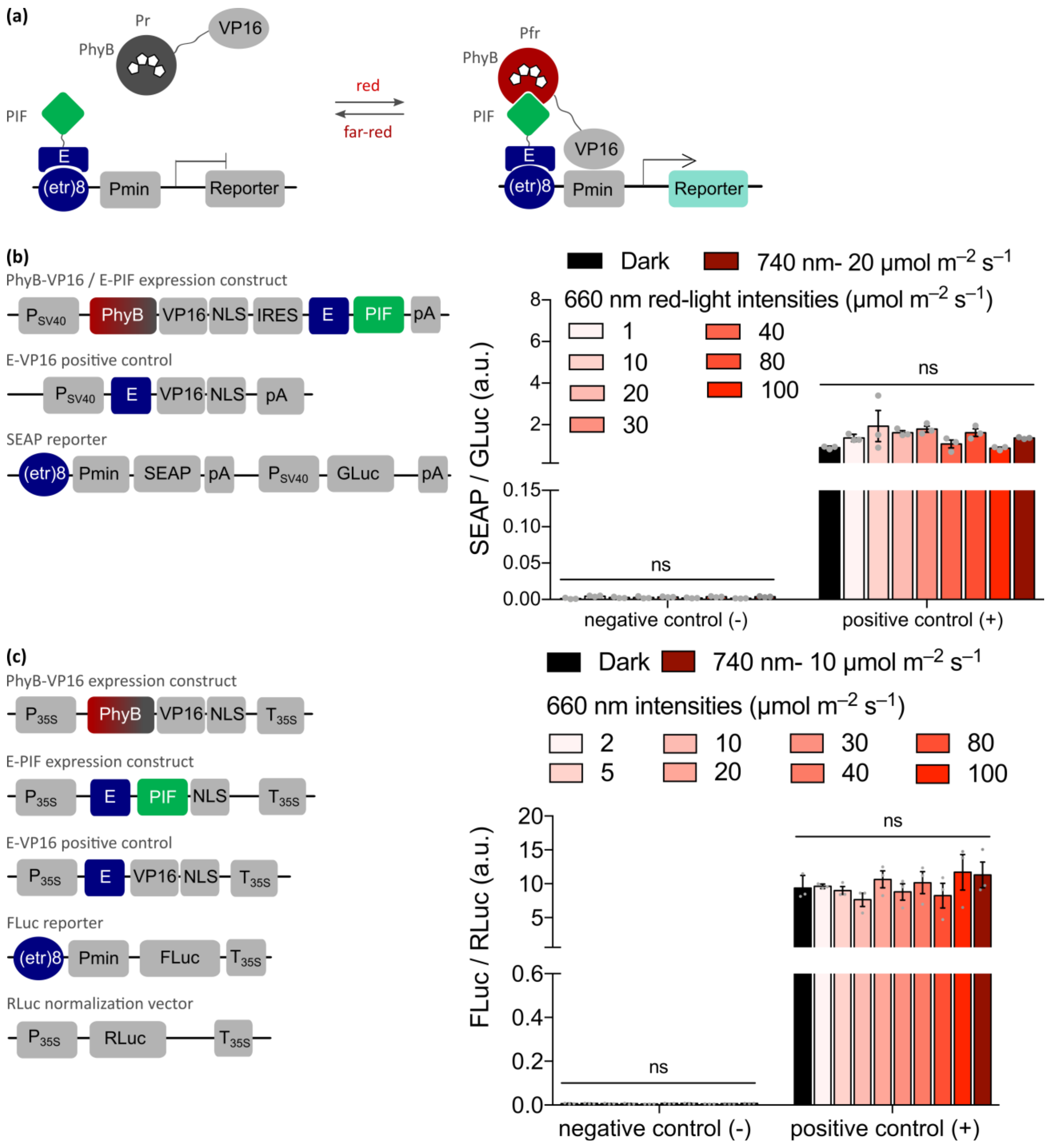

Supports Fig. 2. Light-inducible transgene expression in mammalian cells and Arabidopsis protoplasts. **a**, Schematic of the setup for PhyB/PIF-based light-inducible gene expression. The erythromycin repressor (E)-PIF fusion protein is tethered to a reporter construct harboring eight etr repeats upstream of a minimal CMV promoter (P_{min}). Under red light, the Pfr state of PhyB can interact with PIF, thereby recruiting PhyB-VP16 to trigger reporter expression. Under far-red light, PhyB converts to its Pr state and dissociates from PIF, thus resulting in termination of gene expression. **b**, Gene expression in CHO-K1 cells. (left) Schematics of the constructs for co-expression of PhyB-VP16 and E-PIF from the same promoter, the positive control constitutively expressing E-

VP16, and the SEAP reporter. Abbreviations are NLS, nuclear localization sequence; IRES, internal ribosome entry site; pA, polyadenylation signal. (right) SEAP reporter activity normalized by the *Gaussia* luciferase signal for the positive (+) and negative (-) controls upon 24 h incubation in darkness, under varying red-light intensities (660 nm, 1-100 µE m^{-2} s^{-1}), or under 20 µE m^{-2} s^{-1} 740-nm light. **c**, Gene expression in Arabidopsis protoplasts. (left) Schematics of the constructs for PhyB-VP16 and E-PIF expression, the positive control expressing E-VP16, the firefly luciferase (FLuc) reporter construct, and a construct expressing *Renilla* luciferase (RLuc) for normalization. (right) FLuc reporter activity normalized by the RLuc signal for the positive (+) and negative (-) controls upon 24 h incubation in darkness, under varying red-light intensities (660 nm, 2-100 µE m^{-2} s^{-1}), or under 10 µE m^{-2} s^{-1} 740-nm light. Data represent mean ± s.e.m. of three replicates. Significance values were calculated by one-way ANOVA and revealed no significant differences (ns) between the individual illumination conditions.

Supplementary Figure S7

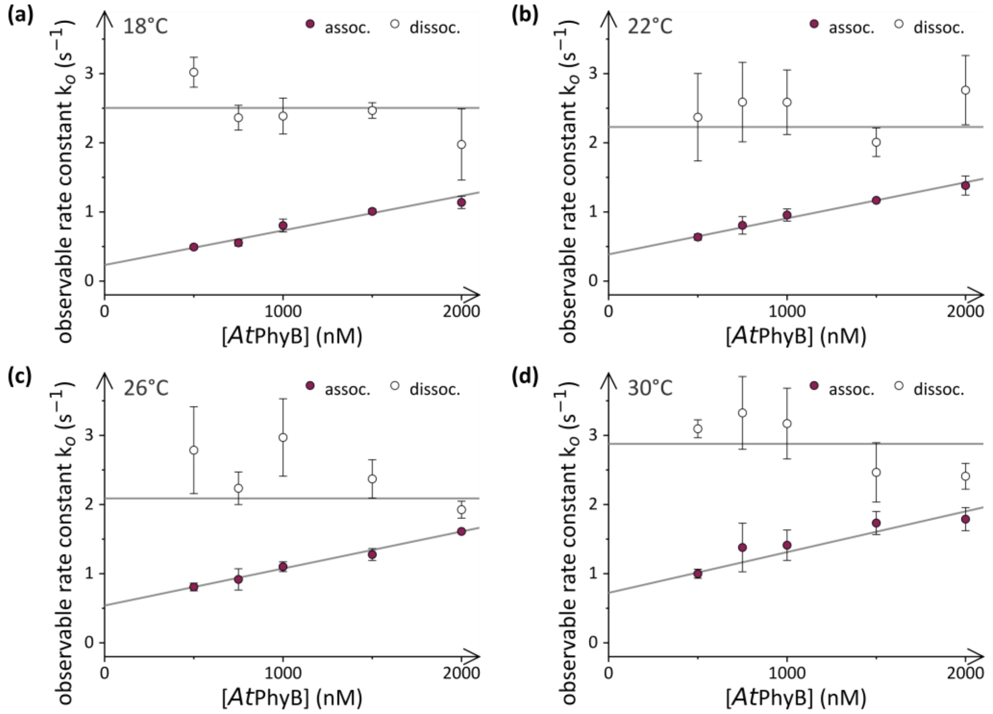

Supports Figs. 3 and 4. Bimolecular association and unimolecular dissociation kinetics of the *At*PhyB:P6A complex upon red-light activation (purple) or under far-red light (white). As in Fig. 3b but at temperatures of 18°C (panel **a**), 22°C (panel **b**), 26°C (panel **c**), and 30°C (panel **d**). Data correspond to mean ± s.d. of three independent measurements. The lines show fits to linear functions. The bimolecular association rate constant upon red-light exposure, k_{aFR}, determined by data fitting was adjusted for the fractional Pfr population of around 73% under these conditions (see Fig. 1b and Table 1).

Supplementary Figure S8

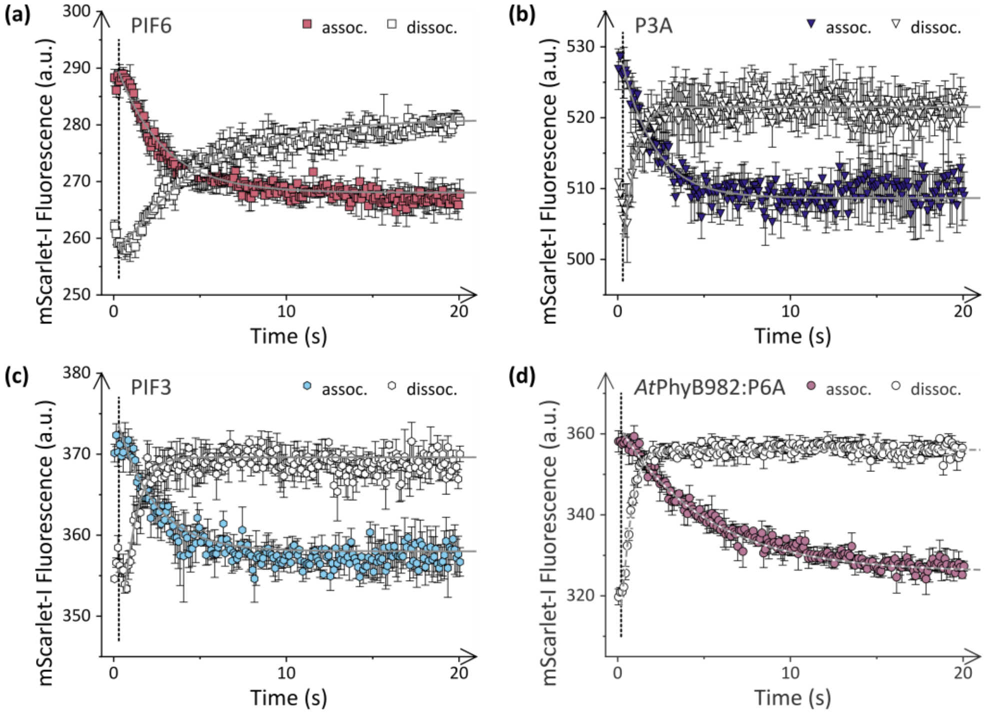

Supports Figs. 3 and 4. Bimolecular association and unimolecular dissociation kinetics of the *At*PhyB:PIF complex. **a**, 20 nM PIF6-mScarlet-I were incubated at 15°C in the presence of 1,000 nM *At*PhyB. Using mScarlet-I fluorescence as readout, the association kinetics (red symbols) were recorded upon red-light illumination (658 nm, 0.5 s, 69 mW cm^{-2}), where the dashed line marks the onset of illumination. A fit of the data to a single-exponential function yielded the observable rate constant k_{oFR}. The dissociation kinetics (white) were acquired under constant 733-nm light (42 mW cm^{-2}) and evaluated according to a sequential reaction model to determine the observable rate constant k_{oR}. **b**, As in panel a but for 20 nM P3A-mScarlet-I mixed with 1,000 nM *At*PhyB. **c**, As in panel a but for 20 nM PIF3-mScarlet-I mixed with 1,000 nM *At*PhyB. **d**, As in panel a but for 30 nM P6A-mScarlet-I mixed with 2,000 nM *At*PhyB (1-982). Data in panels a-d represent mean ± s.d. of three independent experiments.

Supplementary Figure S9

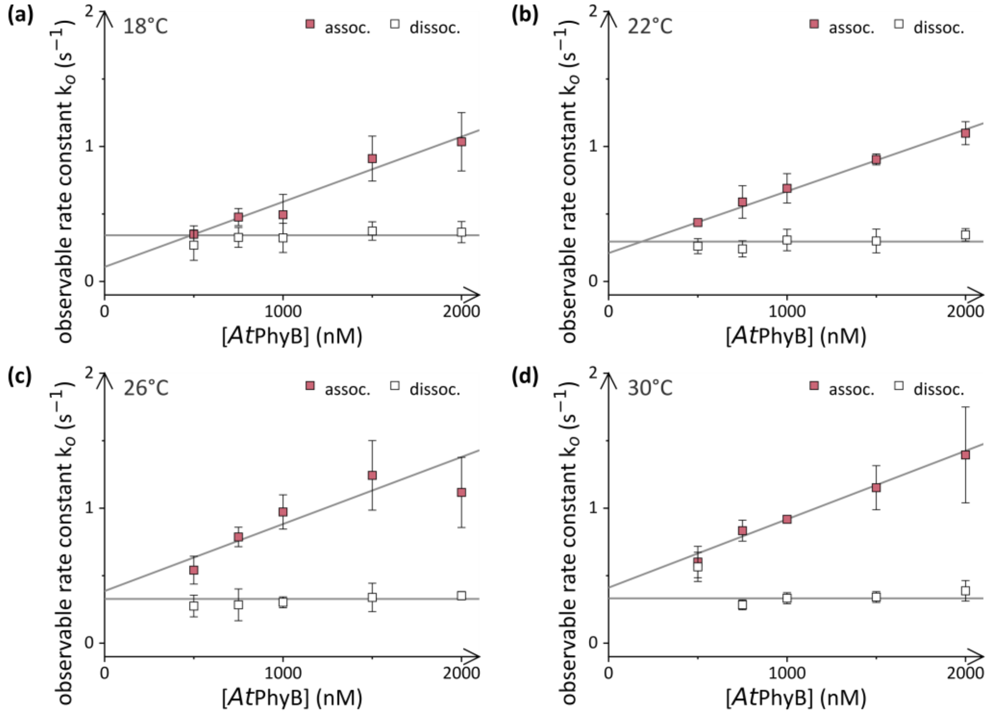

Supports Figs. 3 and 4. Bimolecular association and unimolecular dissociation kinetics of the *At*PhyB:PIF6 complex upon red-light activation (red) or under far-red light (white). As in Fig. 3c but at temperatures of 18°C (panel **a**), 22°C (panel **b**), 26°C (panel **c**), and 30°C (panel **d**). Data correspond to mean ± s.d. of three independent measurements. The lines show fits to linear functions. The bimolecular association rate constant upon red-light exposure, k_{aFR}, determined by data fitting was adjusted for the fractional Pfr population of around 73% under these conditions (see Fig. 1b and Table 1).

Supplementary Figure S10

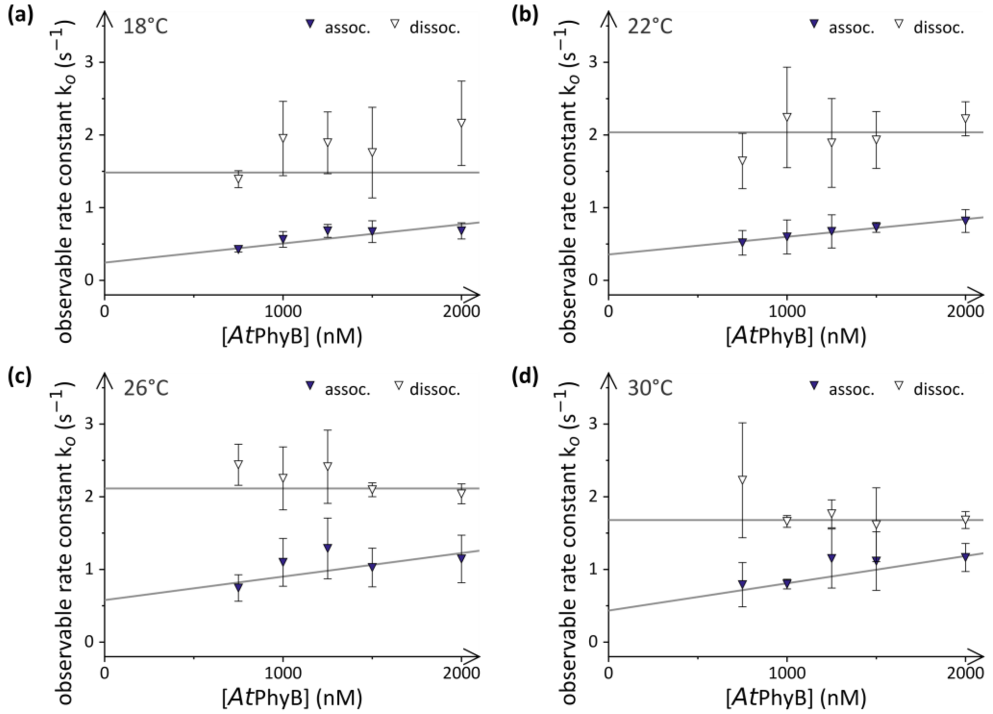

Supports Figs. 3 and 4. Bimolecular association and unimolecular dissociation kinetics of the *At*PhyB:P3A complex upon red-light activation (blue) or under far-red light (white). As in Fig. 3d but at temperatures of 18°C (panel **a**), 22°C (panel **b**), 26°C (panel **c**), and 30°C (panel **d**). Data correspond to mean ± s.d. of three independent measurements. The lines show fits to linear functions. The bimolecular association rate constant upon red-light exposure, k_{aFR}, determined by data fitting was adjusted for the fractional Pfr population of around 73% under these conditions (see Fig. 1b and Table 1).

Supplementary Figure S11

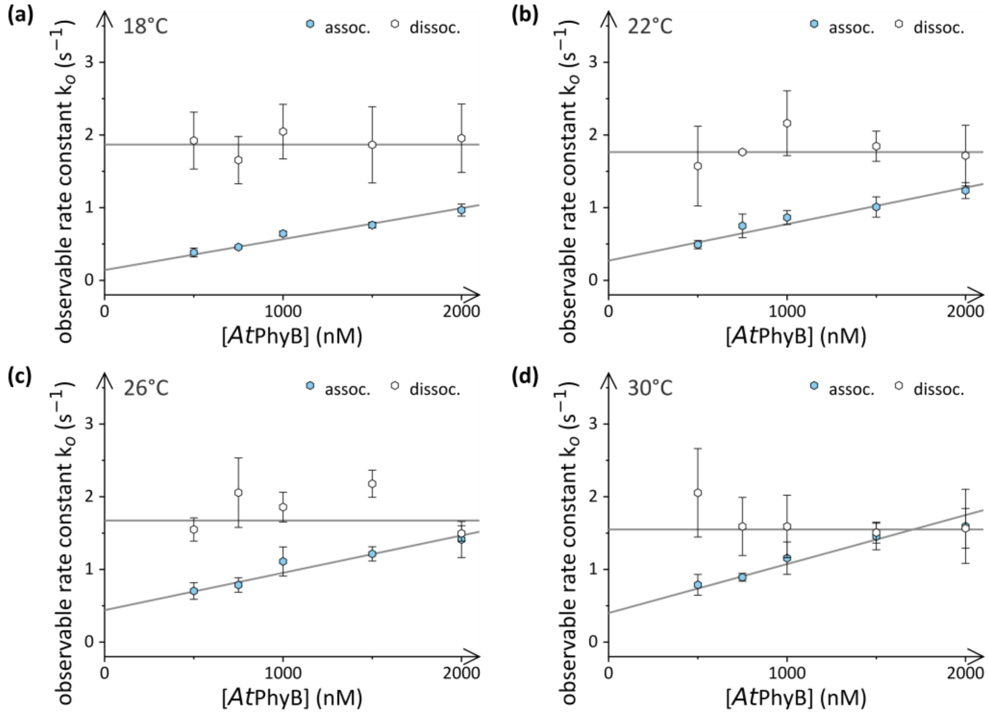

Supports Figs. 3 and 4. Bimolecular association and unimolecular dissociation kinetics of the *At*PhyB:PIF3 complex upon red-light activation (light blue) or under far-red light (white). As in Fig. 3e but at temperatures of 18°C (panel **a**), 22°C (panel **b**), 26°C (panel **c**), and 30°C (panel **d**). Data correspond to mean ± s.d. of three independent measurements. The lines show fits to linear functions. The bimolecular association rate constant upon red-light exposure, k_{aFR}, determined by data fitting was adjusted for the fractional Pfr population of around 73% under these conditions (see Fig. 1b and Table 1).

Supplementary Figure S12

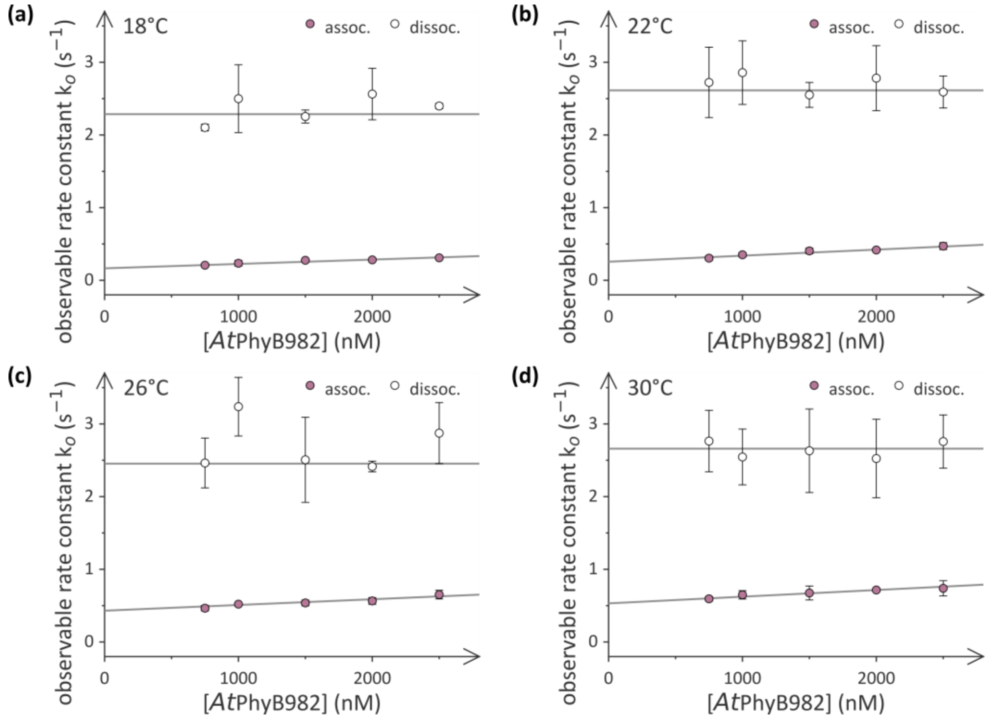

Supports Figs. 3 and 4. Bimolecular association and unimolecular dissociation kinetics of the *At*PhyB982:P6A complex upon red-light activation (pale purple) or under far-red light (white). As in Fig. 3f but at temperatures of 18°C (panel **a**), 22°C (panel **b**), 26°C (panel **c**), and 30°C (panel **d**). Data correspond to mean ± s.d. of three independent measurements. The lines show fits to linear functions. The bimolecular association rate constant upon red-light exposure, k_{aFR}, determined by data fitting was adjusted for the fractional Pfr populations in *At*PhyB982 under these conditions (see Suppl. Fig. S3e).

Supplementary Figure S13

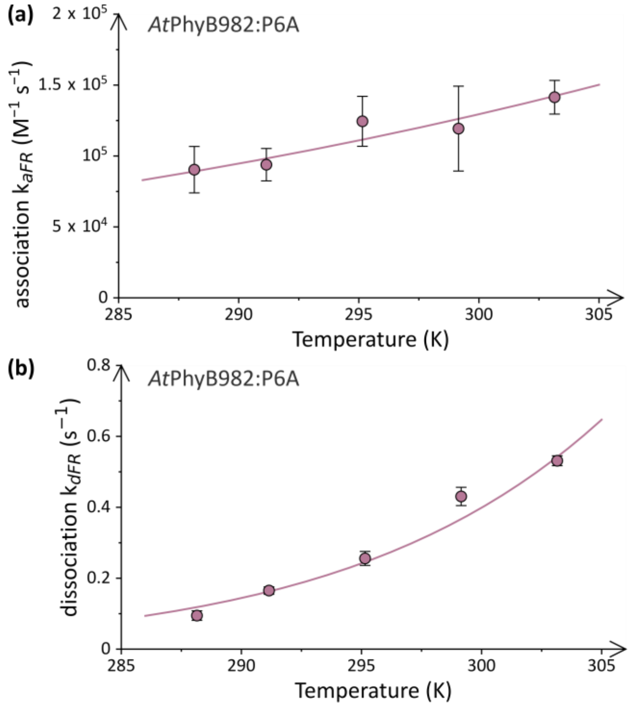

Supports Fig. 4. Temperature dependence of the *At*PhyB (1-982):P6A interaction dynamics. **a**, The bimolecular association rate constant k_{aFR} upon red-light activation as a function of temperature. The line denotes fits to the Arrhenius equation. **b**, As in panel a but for the unimolecular dissociation rate constant k_{dFR} upon red-light activation. Data in panels a and b represent the results from nonlinear least-squares fitting of the underlying data with the error bar denoting the asymptotic standard errors.

Supplementary Figure S14

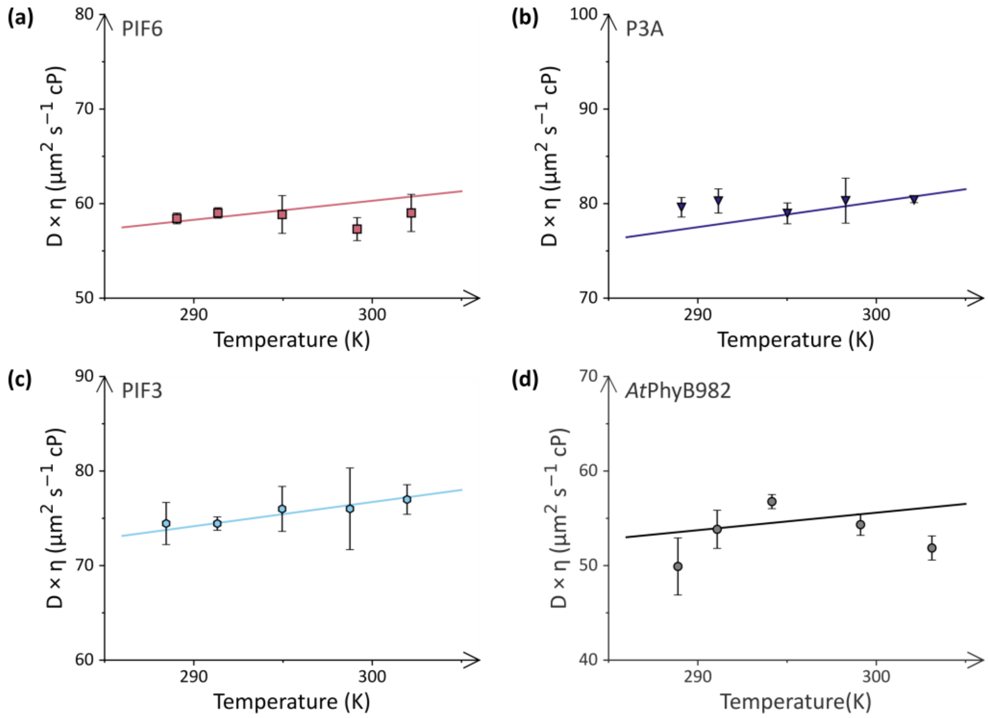

Supports Fig. 5. Hydrodynamics of *At*PhyB and PIF variants at different temperatures. **a**, Diffusion coefficients of PIF6A-mScarlet-I multiplied by viscosity at different temperatures. Fluorescence was detected at 510 nm excitation through a 526-nm long-pass emission filter, and data represent mean ± s.d. of three measurements. The line denotes a fit to the Stokes-Einstein equation. **b**, As in panel a but for P3A-mScarlet-I. **c**, As in panel a but for PIF3-mScarlet-I. **d**, As in panel a but for *At*PhyB (1-982). Fluorescence was excited at 640 nm and detected through a 659-nm long-pass filter.

Supplementary Figure S15

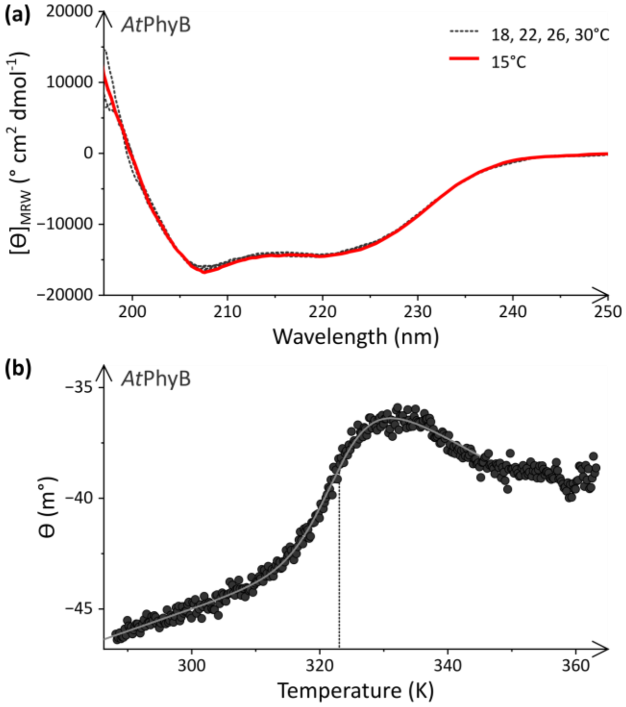

Supports Fig. 5. Analysis of *At*PhyB thermal stability by circular dichroism (CD). **a**, Far-UV CD spectra of dark-adapted *At*PhyB PCM at 15°C (red curve). The dotted grey curves show CD spectra acquired at 18, 22, 26, and 30°C. **b**, The thermal stability of dark-adapted *At*PhyB PCM was investigated by monitoring the CD signal at 222 nm while raising the temperature gradually. The grey line represents a fit to a two-state unfolding model. The vertical dotted line marks the midpoint of unfolding at 49.9°C.

Supplementary Figure S16

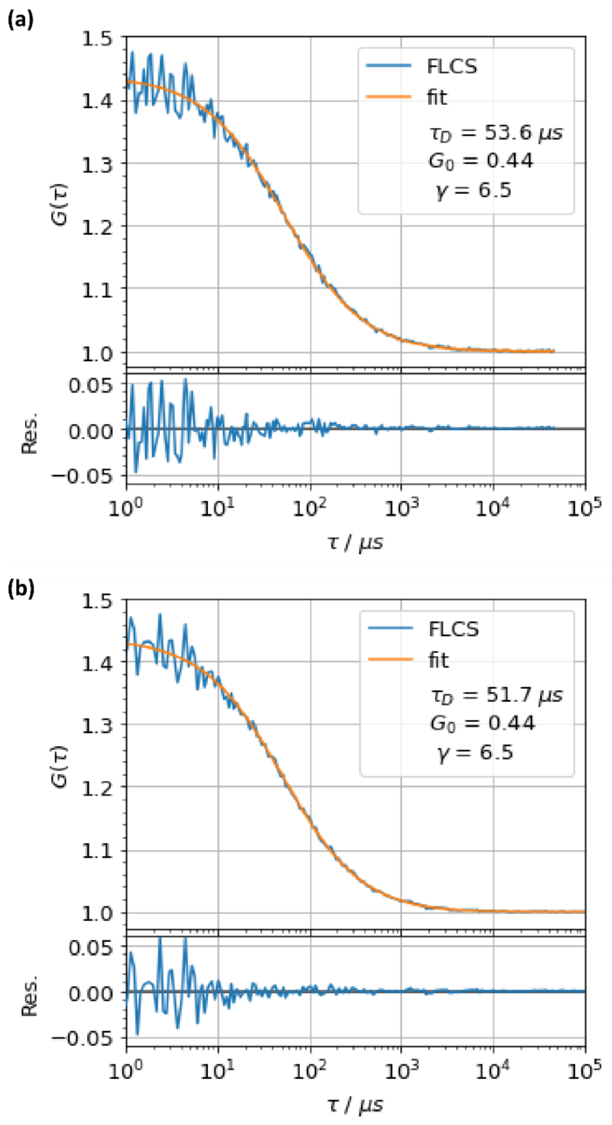

Supports Fig. 5. Benchmarking of custom Python script for fluorescence lifetime correlation spectroscopy (FLCS). The autocorrelation function of Rhodamine B in water was calculated with FLCS to remove the influence of the detector after-pulsing. **a**, The upper panel shows the autocorrelation function calculated with the custom algorithm, and the lower panel reports the residuals between the autocorrelated data and a fit to eq. (10) with γ fixed at a value of 6.5. **b**, As in panel a, but the data were autocorrelated with the SymPhoTime software package.

Supplementary Figure S17

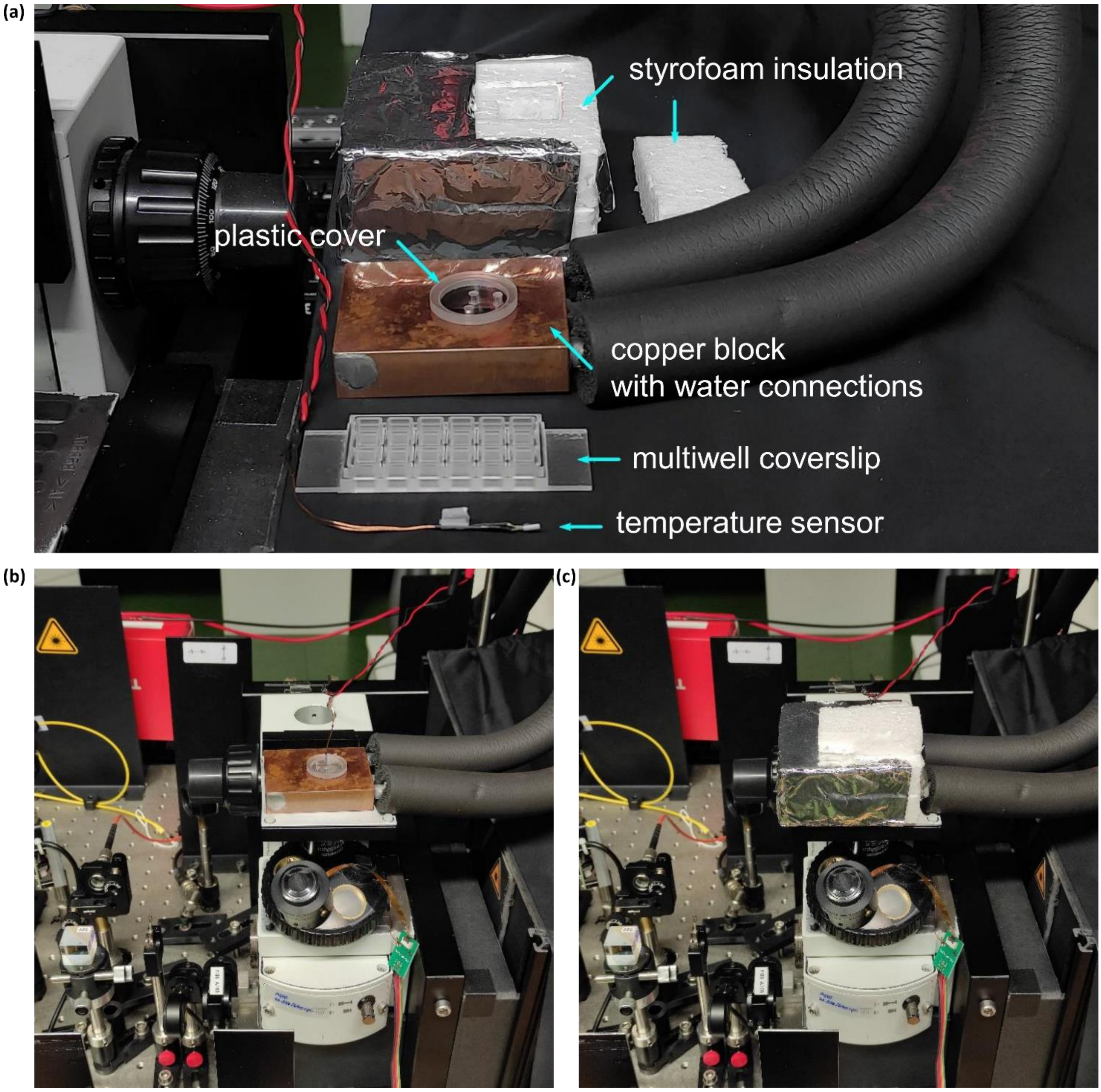

Supports Fig. 5. Customized cooling/heating device for temperature-dependent diffusion measurements. **a**, Overview of all components. The copper block has an opening on the top that allows access to the sample chamber in the multi-well coverslip. This opening is closed with a transparent plastic cover. **b**, Assembled cooling/heating device on the microscope stage. The multi-well coverslip is completely enclosed by the copper block, and the temperature sensor is fed through the plastic cover. **c**, An additional styrofoam cover provided thermal insulation during the measurements.

Supplementary Table S1 – Hydrodynamic properties of the *At*PhyB, PIF3, and PIF6 variants.

**Protein**	R_h **(nm)**	D **(µm² s⁻¹)**[a]	k_{bi} **(M⁻¹ s⁻¹)**[b]
P6A	2.58 ± 0.02[c]	74.7 ± 1.2	$(5.6 \pm 0.1) \times 10^6$
PIF6	3.64 ± 0.03	52.6 ± 0.5	$(5.8 \pm 0.1) \times 10^6$
P3A	2.74 ± 0.01	71.2 ± 0.9	$(5.7 \pm 0.1) \times 10^6$
PIF3	2.86 ± 0.01	59.6 ± 1.8	$(5.3 \pm 0.1) \times 10^6$
*At*PhyB PCM	2.36 ± 0.04	74.4 ± 1.5	n.a.
*At*PhyB (1-982)	3.95 ± 0.04	49.9 ± 3.0	$(6.2 \pm 0.2) \times 10^6$

[a]: Values determined at 15°C.

[b]: Bimolecular encounter rates calculated according to von Smoluchowski with diffusion coefficients determined at 15°C. The first four rows pertain to the interaction with the *At*PhyB PCM. The final row shows refers to the interaction of *At*PhyB (1-982) with P6A.

[c]: Confidence intervals denote the asymptotic standard errors from nonlinear least-squares fitting of the underlying data.

Supplementary Table S2 – Parameters used in the analysis of the attenuation effect.

**Parameter ‡**	**Value**	**Dimension**
k_p #	0.15	mW^{-1} cm^2 s^{-1}
k_q / k_p	0.27 / 0.73	
k_{aR} *	10^{-7}	nM^{-1} s^{-1}
k_{dR} †	18	s^{-1}
k_{aFR}	5.87×10^{-4}	nM^{-1} s^{-1}
k_{dFR}	0.07	s^{-1}
k_{rec} ‖	4.5×10^{-5}	s^{-1}
$[At\text{PhyB}]_{total}$	1,000	nM
$[\text{PIF}]_{total}$	20	nM

‡: All rate constants were determined at 15°C.

#: The actual unimolecular rate constant for the Pr→Pfr conversion at a given light intensity *I* (in units of mW cm^{-2}) equates to $k_p \times I$.

*: To prevent division by zero when solving eq. (20), a small value was used for k_{aR} rather than zero.

†: The value for k_{dR} was taken from the global analysis of the association kinetics for P6A at 15°C.

‖: The values for k_{rec} at 15°C, listed in the table, and the associated Arrhenius activation energy of 9.4×10^4 J mol^{-1} K^{-1} were derived from the values reported for full-length *At*PhyB *in vivo* (36).

Supplementary Table S3 – Oligonucleotide primers used in the study.

Name	Sequence
**i. Constructs for bacterial protein production**	
mScarlet-I-f	5’-TGGTTCTGCCGGCTCCGCTGGTATGGTGAGCAAAGGTGAAGCCGT-3’
mScarlet-I-r	5’-GTCGACCCCGGGTCCCATTTATTTATACAGTTCATCCATACCACC-3’
pET19b-SUMO-backbone-f	5’-GTATGGATGAACTGTATAAATAAATGGGACCCGGGGTCGACCTCGA-3’
pET19b-SUMO-backbone-r	5’-CTTCACCTTTGCTCACCATACCAGCGGAGCCGGCAGAACCAGCA-3’
P3A-f	5’-AACAGATCGGTGGTATGGTTGATGAAGTTGTTGAACTG-3’
P3A-r	5’-CCTTTGCTCACCATACCAGCGGAGCCGGCAGAACCAGC-3’
P6A-f	5’-AACAGATCGGTGGTATGGATCAAGAATATATGGAACTG-3’
P6A-r	5’-GAACCAGCACTATCGGCTTCATACAGATCCATAATGCT-3’
PIF3-f	5’-AACAGATCGGTGGTATGCCGCTGTTTGAACTGTTTCGT-3’
PIF3-r	5’-GAACCAGCACTATCGTGATGATTCAGCCACGGAACAAA-3’
PIF6-f	5’-AACAGATCGGTGGTATGATGTTTCTGCCGACCGATTAT-3’
PIF6-r	5’-GAACCAGCACTATCATCCACGTGTTTATTGCTTTCCAG-3’
PhyB N982-f	5’-CATCACCATCACCATCACTAGAA-3’
PhyB N982-r	5’-ATCACCAACAATGCGGCTAAT-3’
**ii. Constructs for reporter-gene assays**	
oKT016	5‘-GCGCCGTCTCGCTCAAAGCCTACACCTTCCTCTTCTTCTTTGG-3‘
oKT083	5‘-GCGCCGTCTCGCTCGTTCGGCGCGTACGAAAAACAATTACGG-3‘
oKT090	5‘-GCGCCGTCTCGCTCGAATGGTTTCCGGAGTCG-3‘
oKT091	5‘-GCGCCGTCTCGCTCACGAACCACCTAACTCATCAATCCCC-3‘
oKT413	5‘-TCTGCCGGCTCTGCCGGCTCTGCCGGCTGTACAATGCCTCTGTTTGAGC-3‘
oKT414	5‘-AAGCCTACACCTTCCTCTTCTTCTTTGGATGATGATTCAACCATGG-3‘
oKT415	5‘-TCTGCCGGCTCTGCCGGCTCTGCCGGCTGTACAATGGTAGATGAAGTTGTGGA-3‘
oKT416	5‘-AAGCCTACACCTTCCTCTTCTTCTTTGGATTTCCAATCTCTCTAG-3‘
oKT493	5‘-ATTTCAGGTCCCGGATCGGAATTGCGGCCGCCCACCATGGTTTCCGGAGTCGGGGGTA-3‘
oKT494	5‘-CCAGCACTACCAGCACTATCGAATTC-3‘
oROF580	5‘-GCGCCGTCTCGCTCGAATGCCCCGCCCCAAGCT-3‘
oROF581	5‘-GCGCCGTCTCGCTCACGAACCGCTGTACGCGGACGC-3‘
oROF582	5‘-GCGCCGTCTCGCTCGTTCGAGTGCTGGTAGTGCTGGTAG-3‘
oROF583	5‘-GCGCCGTCTCGCTCAAAGCCTACACCTTCCTCTTCTTCTT-3‘
oROF584	5‘-GCGCCGTCTCGCTCGTTCGAGTGCTGGTAGTGCTGGTAGTGCTGGTTGCAGGTT AAGCGATCAA-3‘
oROF585	5‘-GCGCCGTCTCGCTCAAAGCCTACACCTTCCTCTTCTTCTTTGGTGCCTCATACAAATCCATG-3‘
oROF586	5‘-GCGCCGTCTCGCTCGTTCGTCTGCCGGCTCTGCCGGCTC-3‘

Supplementary Table S4 – Identity of the protein constructs used in the study.

Construct	Description	Reference
**i. Constructs for bacterial protein production**		
pYC14	pET19-SUMO, expression His$_6$-TEV-SUMO-P6A-linker-mScarlet-I<br>HHHHHHAENLYFQGHTGSDSEVNQEAKPEVKPEVKPETHINLKVSDGSSEIFFKIKKTTPLRRLMEAFA<br>KRQGKEMDSLRFLYDGIRIQADQTPEDLDMEDNDIIEAHREQIGGMDQEYMELVFENGQILAKGQRS<br>NVSLHNQRTKSIMDLYEADSAGSAGSAGMVSKGEAVIKEFMRFKVHMEGSMNGHEFEIEGEGEGRP<br>YEGTQTAKLKVTKGGPLPFSWDILSPQFMYGSRAFIKHPADIPDYYKQSFPEGFKWERVMNFEDGGA<br>VTVTQDTSLEDGTLIYKVKLRGTNFPPDGPVMQKKTMGWEASTERLYPEDGVLKGDIKMALRLKDGG<br>RYLADFKTTYKAKKPVQMPGAYNVDRKLDITSHNEDYTVVEQYERSEGRHSTGGMDELYK	This work
pYC15	pET19-SUMO, expression His$_6$-TEV-SUMO-PIF6(1-100)-linker-mScarlet-I<br>HHHHHHAENLYFQGHTGSDSEVNQEAKPEVKPEVKPETHINLKVSDGSSEIFFKIKKTTPLRRLMEAFA<br>KRQGKEMDSLRFLYDGIRIQADQTPEDLDMEDNDIIEAHREQIGGMMFLPTDYCCRLSDQEYMELVF<br>ENGQILAKGQRSNVSLHNQRTKSIMDLYEAEYNEDFMKSIIHGGGGAITNLGDTQVVPQSHVAAAHE<br>TNMLESNKHVDDSAGSAGSAGMVSKGEAVIKEFMRFKVHMEGSMNGHEFEIEGEGEGRPYEGTQT<br>AKLKVTKGGPLPFSWDILSPQFMYGSRAFIKHPADIPDYYKQSFPEGFKWERVMNFEDGGAVTVTQD<br>TSLEDGTLIYKVKLRGTNFPPDGPVMQKKTMGWEASTERLYPEDGVLKGDIKMALRLKDGGRYLADF<br>KTTYKAKKPVQMPGAYNVDRKLDITSHNEDYTVVEQYERSEGRHSTGGMDELYK	This work
pYC16	pET19-SUMO, expression His$_6$-TEV-SUMO-PIF3(1-100)-linker-mScarlet-I<br>HHHHHHAENLYFQGHTGSDSEVNQEAKPEVKPEVKPETHINLKVSDGSSEIFFKIKKTTPLRRLMEAFA<br>KRQGKEMDSLRFLYDGIRIQADQTPEDLDMEDNDIIEAHREQIGGMPLFELFRLTKAKLESAQDRNPS<br>PPVDEVVELVWENGQISTQSQSSRSRNIPPPQANSSRAREIGNGSKTTMVDEIPMSVPSLMTGLSQD<br>DDFVPWLNHHDSAGSAGSAGMVSKGEAVIKEFMRFKVHMEGSMNGHEFEIEGEGEGRPYEGTQTA<br>KLKVTKGGPLPFSWDILSPQFMYGSRAFIKHPADIPDYYKQSFPEGFKWERVMNFEDGGAVTVTQDT<br>SLEDGTLIYKVKLRGTNFPPDGPVMQKKTMGWEASTERLYPEDGVLKGDIKMALRLKDGGRYLADFK<br>TTYKAKKPVQMPGAYNVDRKLDITSHNEDYTVVEQYERSEGRHSTGGMDELYK	This work
pYC19	pET19-SUMO, expression His$_6$-TEV-SUMO-P3A-linker-mScarlet-I<br>HHHHHHAENLYFQGHTGSDSEVNQEAKPEVKPEVKPETHINLKVSDGSSEIFFKIKKTTPLRRLMEAFA<br>KRQGKEMDSLRFLYDGIRIQADQTPEDLDMEDNDIIEAHREQIGGMVDEVVELVWENGQISTQSQSS<br>RSRNIPPPQANSSRAREIGNDSAGSAGSAGMVSKGEAVIKEFMRFKVHMEGSMNGHEFEIEGEGEG<br>RPYEGTQTAKLKVTKGGPLPFSWDILSPQFMYGSRAFIKHPADIPDYYKQSFPEGFKWERVMNFEDG<br>GAVTVTQDTSLEDGTLIYKVKLRGTNFPPDGPVMQKKTMGWEASTERLYPEDGVLKGDIKMALRLKD<br>GGRYLADFKTTYKAKKPVQMPGAYNVDRKLDITSHNEDYTVVEQYERSEGRHSTGGMDELYK	This work
pYC38	pET19-SUMO, expression His$_6$-TEV-SUMO-mScarlet-I<br>HHHHHHAENLYFQGHTGSDSEVNQEAKPEVKPEVKPETHINLKVSDGSSEIFFKIKKTTPLRRLMEAFA<br>KRQGKEMDSLRFLYDGIRIQADQTPEDLDMEDNDIIEAHREQIGGMVSKGEAVIKEFMRFKVHMEGS<br>MNGHEFEIEGEGEGRPYEGTQTAKLKVTKGGPLPFSWDILSPQFMYGSRAFIKHPADIPDYYKQSFPE<br>GFKWERVMNFEDGGAVTVTQDTSLEDGTLIYKVKLRGTNFPPDGPVMQKKTMGWEASTERLYPED<br>GVLKGDIKMALRLKDGGRYLADFKTTYKAKKPVQMPGAYNVDRKLDITSHNEDYTVVEQYERSEGRH<br>STGGMDELYK	This work
pYC63	pCDF-Duet, expression *At*PhyB (1-982)<br>MVSGVGGSGGGRGGGRGGEEEPSSSHTPNNRRGGEQAQSSGTKSLRPRSNTESMSKAIQQYTVDA<br>RLHAVFEQSGESGKSFDYSQSLKTTTYGSSVPEQQITAYLSRIQRGGYIQPFGCMIAVDESSFRIIGYSEN<br>AREMLGIMPQSVPTLEKPEILAMGTDVRSLFTSSSSILLERAFVAREITLLNPVWIHSKNTGKPFYAILHRI<br>DVGVVIDLEPARTEDPALSIAGAVQSQKLAVRAISQLQALPGGDIKLLCDTVVESVRDLTGYDRVMVYK<br>FHEDEHGEVVAESKRDDLEPYIGLHYPATDIPQASRFLFKQNRVRMIVDCNATPVLVVQDDRLTQSM	This work

	CLVGSTLRAPHGCHSQYMANMGSIASLAMAVIINGNEDDGSNVASGRSSMRLWGLVVCHHTSSRCI<br>PFPLRYACEFLMQAFGLQLNMELQLALQMSEKRVLRTQTLLCDMLLRDSPAGIVTQSPSIMDLVKCD<br>GAAFLYHGKYYPLGVAPSEVQIKDVVEWLLANHADSTGLSTDSLGDAGYPGAAALGDAVCGMAVAYI<br>TKRDFLFWFRSHTAKEIKWGGAKHHPEDKDDGQRMHPRSSFQAFLEVVKSRSQPWETAEMDAIHSL<br>QLILRDSFKESEAAMNSKVVDGVVQPCRDMAGEQGIDELGAVAREMVRLIETATVPIFAVDAGGCIN<br>GWNAKIAELTGLSVEEAMGKSLVSDLIYKENEATVNKLLSRALRGDEEKNVEVKLKTFSPELQGKAVFV<br>VVNACSSKDYLNNIVGVCFVGQDVTSQKIVMDKFINIQGDYKAIVHSPNPLIPPIFAADENTCCLEWN<br>MAMEKLTGWSRSEVIGKMIVGEVFGSCCMLKGPDALTKFMIVLHNAIGGQDTDKFPFPFFDRNGKF<br>VQALLTANKRVSLEGKVIGAFCFLQIPSPELQQALAVQRRQDTECFTKAKELAYICQVIKNPLSGMRFA<br>NSLLEATDLNEDQKQLLETSVSCEKQISRIVGDHHHHHH	
pDG458	pCDF-Duet, expression *At*PhyB (1-651)	Golonka *et al.* (1)
	MVSGVGGSGGGRGGGRGGEEEPSSSHTPNNRRGGEQAQSSGTKSLRPRSNTESMSKAIQQYTVDA<br>RLHAVFEQSGESGKSFDYSQSLKTTTYGSSVPEQQITAYLSRIQRGGYIQPFGCMIAVDESSFRIIGYSEN<br>AREMLGIMPQSVPTLEKPEILAMGTDVRSLFTSSSSILLERAFVAREITLLNPVWIHSKNTGKPFYAILHRI<br>DVGVVIDLEPARTEDPALSIAGAVQSQKLAVRAISQLQALPGGDIKLLCDTVVESVRDLTGYDRVMVYK<br>FHEDEHGEVVAESKRDDLEPYIGLHYPATDIPQASRFLFKQNRVRMIVDCNATPVLVVQDDRLTQSM<br>CLVGSTLRAPHGCHSQYMANMGSIASLAMAVIINGNEDDGSNVASGRSSMRLWGLVVCHHTSSRCI<br>PFPLRYACEFLMQAFGLQLNMELQLALQMSEKRVLRTQTLLCDMLLRDSPAGIVTQSPSIMDLVKCD<br>GAAFLYHGKYYPLGVAPSEVQIKDVVEWLLANHADSTGLSTDSLGDAGYPGAAALGDAVCGMAVAYI<br>TKRDFLFWFRSHTAKEIKWGGAKHHPEDKDDGQRMHPRSSFQAFLEVVKSRSQPWETAEMDAIHSL<br>QLILRDSFKESEAAMNSKVVDGVVQPCRDMAGEQGIDELGAHHHHHH	
**ii. Constructs for reporter-gene assays**		
pPF035	etr$_8$-P$_{hCMVmin}$-SEAP-T$_{BGH}$-P$_{SV40}$-GLuc-T$_{SV40}$	Golonka *et al.* (1)
PIF3	P$_{SV40}$-PhyB(1-650)-VP16-NLS-IRES-E-PIF3(1-100)-T$_{SV40}$	Golonka *et al.* (1)
P3A	P$_{SV40}$-PhyB(1-650)-VP16-NLS-IRES-E-PIF3(26-66)-T$_{SV40}$	Golonka *et al.* (1)
PIF6	P$_{SV40}$-PhyB(1-650)-VP16-NLS-IRES-E-PIF6(1-100)-T$_{SV40}$	Golonka *et al.* (1)
P6A	P$_{SV40}$-PhyB(1-650)-VP16-NLS-IRES-E-PIF6(10-52)-T$_{SV40}$	Golonka *et al.* (1)
pWW035	P$_{SV40}$-E-VP16-T$_{SV40}$	Müller *et al.* (2)
pROF537	P$_{35S}$-*At*PhyB(1-650)-VP16-NLS-T$_{35S}$	Ochoa-Fernandez *et al.* (3)
pKT1022(PIF3)	P$_{35S}$-E-*At*PIF3(1-100)-NLS-T$_{35S}$	This work
pKT1024(P3A)	P$_{35S}$-E-*At*PIF3(26-66)-NLS-T$_{35S}$	This work
pROF490 (PIF6)	P$_{35S}$-E-*At*PIF6(1-100)-NLS-T$_{35S}$	Ochoa-Fernandez *et al.* (3)
pROF491(P6A)	P$_{35S}$-E-*At*PIF6(10-52)-NLS-T$_{35S}$	Ochoa-Fernandez *et al.* (3)
pGB0109	P$_{35S}$-RLuc-T$_{nos}$<br>GoldenBraid plasmid used for normalization in protoplast assays	Sarrion-Perdigones *et al.* (4)
pRSET	stuffer DNA vector used for unexpressed transformation control in protoplasts and CHO cells	Thermo Fisher
pROF052 (pMZ836)	etr$_8$–P$_{hCMVmin}$-FLuc-pA	Müller *et al.* (2)
pKT531	P$_{35S}$-E-VP16-NLS-T$_{35S}$	This work

pKT1026 (FL_PIF6)	P_{SV40}-PhyB$_{(1\text{-}1172)}$-VP16-NLS-IRES-E-PIF6$_{(1\text{-}100)}$-T_{SV40}	This work
pKT1027 (FL_P6A)	P_{SV40}-PhyB$_{(1\text{-}1172)}$-VP16-NLS-IRES-E-PIF6$_{(10\text{-}52)}$-T_{SV40}	This work
pKT1028 (FL_PIF3)	P_{SV40}-PhyB$_{(1\text{-}1172)}$-VP16-NLS-IRES-E-PIF3$_{(1\text{-}100)}$-T_{SV40}	This work
pKT1029 (FL_P3A)	P_{SV40}-PhyB$_{(1\text{-}1172)}$-VP16-NLS-IRES-E-PIF3$_{(26\text{-}66)}$-T_{SV40}	This work

References

1. Golonka, D., P. Fischbach, S.G. Jena, J.R.W. Kleeberg, L.-O. Essen, J.E. Toettcher, M.D. Zurbriggen, and A. Möglich. 2019. Deconstructing and repurposing the light-regulated interplay between Arabidopsis phytochromes and interacting factors. *Commun. Biol.* 2:448.

2. Müller, K., R. Engesser, S. Schulz, T. Steinberg, P. Tomakidi, C.C. Weber, R. Ulm, J. Timmer, M.D. Zurbriggen, and W. Weber. 2013. Multi-chromatic control of mammalian gene expression and signaling. *Nucleic Acids Res.* 41:e124.

3. Ochoa-Fernandez, R., N.B. Abel, F.-G. Wieland, J. Schlegel, L.-A. Koch, J.B. Miller, R. Engesser, G. Giuriani, S.M. Brandl, J. Timmer, W. Weber, T. Ott, R. Simon, and M.D. Zurbriggen. 2020. Optogenetic control of gene expression in plants in the presence of ambient white light. *Nat. Methods*. 17:717–725.

4. Sarrion-Perdigones, A., M. Vazquez-Vilar, J. Palací, B. Castelijns, J. Forment, P. Ziarsolo, J. Blanca, A. Granell, and D. Orzaez. 2013. GoldenBraid 2.0: A Comprehensive DNA Assembly Framework for Plant Synthetic Biology. *Plant Physiol.* 162:1618–1631.
