# Supplementary material for: Plant phytochrome interactions decode light and temperature signals
Source: Plant Cell. 2024 Sep 11;36(12):4819–39. doi: 10.1093/plcell/koae249 (PMC11638003; doi:10.1093/plcell/koae249)
Supplement: koae249_Supplementary_Data [file tpc.00891.2023-s06.pdf › tpc.00891.2023-s06.pdf]

THE PLANT CELL

Plant Phytochrome Interactions Decode Light and Temperature Signals

Chengwei Yi, Uwe Gerken, Kun Tang, Michael Philipp, Matias Zurbriggen, Jürgen Köhler, and Andreas Möglich

Review Timeline:

Submission Date:	26-Jul-2023
Editorial Decision:	19-Sep-2023
Resubmission Received:	03-May-2024
Editorial Decision:	23-May-2024
Revision Received:	05-Aug-2024
Accepted:	21-Aug-2024

Prof. Dr. Andreas Möglich
Universität Bayreuth, Lehrstuhl für Biochemie II
Bayreuth 95447
Germany

Dear Dr. Möglich:

Thank you for choosing to send your manuscript entitled "Plant Phytochrome Interactions Decode Light and Temperature Signals" for consideration at The Plant Cell. Your submission has been evaluated by members of the editorial board as well as expert reviewers in your field, and we regret to inform you that we are not able to recommend the publication of this manuscript in its current form. We have had input from multiple scientists and we have solicited post-review comments.

Our decision is based on a number of considerations. First, the manuscript is heavily computational and concern was raised by Reviewer 1 that questions the robustness of the modeling and the potential need to consider other models. Modeling approaches beyond traditional curving fitting along with mechanistic insights that would connect "local" kinetic parameters to "global" behavior of the dynamic photoconversion process would greatly enhance the significance and general interest of such studies. The emphasis on computational evidence also requires the TPC readership without such expertise, e.g. Reviewer 2 amongst other non-computational plant biologists, to accept the robustness of such models. Hence the target audience in this type of work might be better served by communication in another journal rather than revision of this manuscript. Second, the use of the N-terminal truncated phyB (PhyBN) is problematic for modeling since its structure differs substantially from the structure of the full-length protein based on recent cryoEM studies. A comment or discussion therein is also warranted even though the structure for the full-length Pfr form of plant phytochromes is not yet known.

That said, we would be willing to re-consider a new manuscript that fully addressed the concerns raised during this review process and outlined above. If you decide to resubmit to The Plant Cell, it may be evaluated as a new submission subject to full assessment by the editorial board, and if sent for external review, new reviewers might be chosen. Reviewers will be asked to assess your work as a new manuscript (i.e. are the claims fully supported by the data and do the results presented move the field forward?), and not only whether previous reviewer comments have been addressed.

The Plant Cell now requires authors to complete and submit an author revisions checklist upon submission of a revised manuscript. The aim of the checklist is to aid authors in preparing a high-quality manuscript, facilitate the review and assessment of revised manuscripts, and help to ensure that journal standards are maintained across the board. If your manuscript is accepted, the completed checklist will be published as supplemental material attached to the article online. Please download a copy of the checklist (a fillable PDF form) at this link, for submission with your revised manuscript: https://tpc.msubmit.net/html/Author_Revisions_Checklist.pdf.

Note also that supplemental materials should be restricted to large datasets and tables, presentation of replicates, and validation of reagents, methods, or genotypes. Any data that are used to support the main claims must be in the main manuscript. Supplemental figure legends must indicate what figure in the main manuscript is supported by the supplemental data presented.

We thank you for your interest in and support of The Plant Cell. We hope we will be able to render a more positive decision on future work.

On behalf of the editorial board,

Reviewing Editor, Board of Reviewing Editors
George Coupland, Senior Editor
Blake Meyers, Editor-in-Chief

The Plant Cell
****
Reviewer comments:

Reviewer #1 (Comments for the Author):

The manuscript by Yi et al. investigates the temperature dependence of photoreception and signaling behaviors in plant phytochrome PhyB. Using a minimalist approach together with a fluorescence reporter, the authors systematically examined how the interactions between PhyB and its downstream signaling partners (specifically, PIF3 and PIF6) are affected by temperature as well as the fluence and quality of light. The topic of this work is interesting, the experimental design is thoughtful, and the kinetic data (from both in vitro and in vivo assays) is comprehensive. As pointed out by the authors, the findings regarding the attenuation effects under high-light conditions and the temperature dependence of the PhyB:PIF interactions may have far-reaching implications on optogenetics applications based on phytochrome photoreceptors.

While using fluorescence quenching of mScarlet-I to probe the PhyB:PIF interactions is a good idea, its entanglement with the reversible photoconversion of PhyB and its temperature-dependent dark reversion process PhyB (Fig. 2a) could complicate data analysis and data interpretation. As such, the processes and kinetic behaviors seem a bit convoluted. To this end, the traditional data fitting and analytical strategy as presented in this work may not be the best way to reveal the underlying drivers and associated kinetics captured by these many time series of in vitro data (e.g. traces on Fig. 2b, Fig. 3). Specifically, I would suggest to perform a joint data analysis (via principal component analysis or singular value decomposition) on these time series . Such a joint analysis would be more informative in 1) providing a global view of the comprehensive data presented in this work. 2) examining the effects and interplay of temperature and light fluence in PhyB signaling, 3) providing cross-validation of the proposed kinetic model and kinetic parameters obtained by global fitting.

Reviewer #2 (Comments for the Author):

Authors adopted fluorescence-based monitoring system to analyze the detailed kinetics of phyBN and PIF6/PIF3 in vitro as the function of light intensity and temperature. Authors found that the interaction itself decreases as temperature increases due to more rapid increase of kd than ka, adding an additional layer of thermosensing mechanism by phyB. Surprisingly, but expectedly in a retrospect, authors found that the interaction decreases at high intensity of light due to the rapid conversion between Pr<>Pfr. The 'high intensity' used here is within a typical range of hypocotyl red fluence response curve exp (0 to 100 umole m-2 s-1), the in vivo interaction could be more complicated as, discussed in the manuscript, the current conclusion is based on phyBN and PIF in vitro. I have only two minor comments.

1. It would be useful to add a Kd graph as the function of temperature in Figure 4. That will help readers to see the change of Kd over the range of temperature.
2. Though figure legends have all necessary information, it will be helpful to have what shapes/colors represent also directly on the graph.

Dear Dr. Coupland, dear Dr. Meyers

we thank you and the reviewers for the thorough study of our manuscript, the valuable comments, and the positive assessment. In light of your and the reviewer's comments, we have conducted additional experiments that address the raised points. We enclose a revised version of our manuscript which addresses these comments as detailed below (original comments in italics, responses in blue):

Editor:

(...)

First, the manuscript is heavily computational and concern was raised by Reviewer 1 that questions the robustness of the modeling and the potential need to consider other models. Modeling approaches beyond traditional curving fitting along with mechanistic insights that would connect "local" kinetic parameters to "global" behavior of the dynamic photoconversion process would greatly enhance the significance and general interest of such studies.

Reviewer #1 raises valid points which we address in our detailed response below. Briefly, we add in the revised manuscript a better explanation of our approach and reasoning for data evaluation. Also, we include additional experiments at other temperatures and using a more extended *At*PhyB variant. Crucially, all these experiments can be fully explained by the same mechanistic model, thereby greatly strengthening and validating it. Said model hinges on a slight and intuitive extension of canonical models generally used for analyzing phytochrome:PIF interactions. Loosely paraphrasing reviewer #2's comments, the experimentally observed light effects are "surprising" but can be explained by the model and are hence "expected in retrospect".

The emphasis on computational evidence also requires the TPC readership without such expertise, e.g. Reviewer 2 amongst other non-computational plant biologists, to accept the robustness of such models. Hence the target audience in this type of work might be better served by communication in another journal rather than revision of this manuscript.

It is true that our manuscript relies on mechanistic modeling to rationalize the counterintuitive and initially bewildering experimental observations (see Fig. 2b-c, Suppl. Fig. S5). Notwithstanding that, at a fundamental level, our study is experimental in nature, not computational. Crucially, our experiments and their mechanistic rationalization advance our knowledge about the interplay of light and temperature cues, an area of paramount importance for plants and the subject of vigorous study. Only by pursuing this integrated approach are we able to elucidate the molecular mechanism underlying the light-dependent phytochrome interactions which is relevant to plant physiology and biotechnological application alike.

Although the precise details of the data evaluation may be demanding to recapitulate in each and every detail, the salient findings and their important implications are not. That is because even at a qualitative level, it is apparent that the current models for describing light-induced Phy:PIF interactions (see blue box in Fig. 6a) utterly fail to account for the experimental data in Fig. 2b. As detailed in the text, the conventional models can neither explain the attenuation of the interaction under continuous strong light nor the secondary association reaction occurring immediately after light exposure ceases. A slight extension of the mechanistic model (see Figs. 2a and 6a) suffices to fully rationalize the experimental observations at both the qualitative and quantitative levels.

As the attenuation and temperature effects that we unravel play out at the level of the PhyB:PIF interaction, they stand to substantially impact on light and temperature reception and signaling

responses of plants. Given that these important effects have been largely neglected to date, we consider our study highly relevant and timely for the broad TPC readership. To convey the key implications and importance of our study, we conduct and discuss simulations for different parameter regimes (see Fig. 6). At least two key aspects can be immediately taken away from the figure, even without knowledge of the mathematical underpinnings. First, the temperature dependence of the PhyB:PIF interaction modulates the light sensitivity of PhyB-dependent responses (see Fig. 6c). Put another way, the PhyB:PIF interaction can contribute to phytochrome thermoreception. Second, contrary to expectations, the extent of the PhyB:PIF interaction (and hence, of the downstream physiological responses) may not monotonically increase with red-light intensity but traverse a maximum at intermediate light intensities before dropping again (see, e.g., Fig. 6d).

To reiterate, we consider the main findings of the study amenable without delving into the full details of the underlying model. At the same time, these details matter, and our manuscript duly includes them in the Methods section, thus allowing scientists to follow and build on our work. Specifically, by providing a steady-state solution to the mechanistic model in analytical form (see equation (18)), we enable the readers to recapitulate Fig. 6 and conduct simulations of their own device, while sparing them the yet-more demanding mathematical aspects, e.g., the numerical solution of differential equations.

To sum up, our findings have profound implications for phytochromes in plants and in optogenetics, as also recognized by the reviewers. Despite the admitted complexity of the study, we therefore consider it highly relevant and important for the general TPC readership.

All that said, we have taken to heart the editor's comments and have striven to streamline our manuscript and make it more palatable for a general audience. In specific, we have relegated several panels of Figs. 3 and 5 to the supplementary information (SI); likewise, we have moved the former table 3 to the SI (now Suppl. Table S1); we have expanded our description of the data analysis (see response to reviewer #1); and we have more clearly labeled the figures throughout the entire manuscript and SI (see response to reviewer #2).

Second, the use of the N-terminal truncated phyB (PhyBN) is problematic for modeling since its structure differs substantially from the structure of the full-length protein based on recent cryoEM studies. A comment or discussion therein is also warranted even though the structure for the full-length Pfr form of plant phytochromes is not yet known.

We fully concur and include additional experiments as follows:

First, we expand our fluorescence-based studies of the PIF interaction to a more extended *At*PhyB construct (see Fig. 2c). Although in repeat attempts, we did not manage to produce the full-length *At*PhyB in sufficient quantity and purity, we prepared a slightly shorter *At*PhyB construct comprising residues 1-982 and including the PAS1, PAS2, and DHp domains in addition to the PhyB N-terminal part (PhyBN). As shown by Vierstra and colleagues (Li *et al. Nature* 2022), this *At*PhyB (1-982) construct can adopt a homodimeric state, as does the full-length receptor. When combining the *At*PhyB (1-982) protein with a PIF variant, we observed attenuation of the interaction under strong red light, similar to our findings for the PhyBN construct. Intriguingly, the attenuation effect was more dramatic and led to near-complete suppression of the interaction at the highest light intensity tested. These findings suggest that the attenuation mechanism may be even more important in the near full-length receptor than in the isolated photosensory module. Further analyses principally tie the more pronounced attenuation to slower PIF association kinetics

(Fig. 3f and Suppl. Fig. S11) in the *At*PhyB (1-982) protein compared to the much-smaller PhyBN construct. In response to reviewer #1, see below, we also include interaction measurements for the *At*PhyB (1-982) protein at higher temperatures and again find attenuation of the PIF interaction under continuous light (Suppl. Fig. S4). Taken together, we conclude that our mechanistic model (Fig. 2a) also applies to the light-dependent interaction of the more extended *At*PhyB variant.

Second, we expand the reporter-gene experiments probing the light-responsive phytochrome interactions to include the full-length *At*PhyB and its interaction with different PIF variants for optogenetic expression control (Fig. 2e). Like for the shorter PhyBN variant (see Fig. 2d), the reporter-gene levels increased under low to moderate red-light exposure but dropped again at higher light intensities. These data indicate that the attenuation effect is also relevant to full-length *At*PhyB in a cellular context.

Taken together, we conclude that the previously undocumented attenuation also plays out for *At*PhyB at or near full length. If anything, attenuation may be even more drastic in this context which is rationalized by the slower association dynamics compared to PhyBN and is fully explained by our mechanistic model.

Reviewer #1:

The manuscript by Yi et al. investigates the temperature dependence of photoreception and signaling behaviors in plant phytochrome PhyB. Using a minimalist approach together with a fluorescence reporter, the authors systematically examined how the interactions between PhyB and its downstream signaling partners (specifically, PIF3 and PIF6) are affected by temperature as well as the fluence and quality of light. The topic of this work is interesting, the experimental design is thoughtful, and the kinetic data (from both in vitro and in vivo assays) is comprehensive. As pointed out by the authors, the findings regarding the attenuation effects under high-light conditions and the temperature dependence of the PhyB:PIF interactions may have far-reaching implications on optogenetics applications based on phytochrome photoreceptors.

We thank the reviewer for the positive comments.

While using fluorescence quenching of mScarlet-I to probe the PhyB:PIF interactions is a good idea, its entanglement with the reversible photoconversion of PhyB and its temperature-dependent dark reversion process PhyB (Fig. 2a) could complicate data analysis and data interpretation.

This reviewer raises several important points which we address as follows.

To examine a possible influence of the mScarlet-I fluorescence measurements on the reversible PhyB photoconversion, we first determined the intensity of the excitation light (wavelength 565 nm) of the fluorescence spectrophotometer. At 0.05 mW cm^{-2}, the intensity of this 565-nm probe light was considerably less than even the lowest red-light (wavelength 658 nm) intensity of 1 mW cm^{-2} used for driving Pr⇄Pfr photoconversion in Fig. 2b. Next, we performed mock experiments like those in Fig. 2b but without applying red light. As shown in the new Suppl. Fig. S4a, under these conditions, the mScarlet-I signal gradually decreased over time which indicates that the probe light can principally drive the Pr→Pfr photoconversion, as this reviewer may have already suspected. However, as the photoconversion kinetics induced by the probe light are slow compared to those driven by the 658-nm light used in our experiments, the measurements are not significantly affected.

Regarding a potential adverse effect of Pfr→Pr dark recovery, we note that all fluorescence experiments in Figs. 2 and 3 and the Suppl. Figs. S4-S11 are based on fluorescence kinetics of 30 s duration or less. Even at 30°C, the highest temperature investigated, the Pfr→Pr dark recovery plays out on much longer timescales over the course of several minutes to hours (see Suppl. Fig. S1b). We hence conclude that dark recovery does not significantly impact on our experiments. On a general note, we however concur that the Pfr→Pr dark recovery is intricately linked to the effective light sensitivity of PhyB under constant illumination, see, e.g., Fig. 6b, as first reported in the pioneering work by Legris *et al. Science* 2016 and Jung *et al. ibid*.

To address this reviewer's comment, we include new experiments as Suppl. Fig. S4a, and we expand the manuscript along the above points.

As such, the processes and kinetic behaviors seem a bit convoluted. To this end, the traditional data fitting and analytical strategy as presented in this work may not be the best way to reveal the underlying drivers and associated kinetics captured by these many time series of in vitro data (e.g. traces on Fig. 2b, Fig. 3). Specifically, I would suggest to perform a joint data analysis (via principal component analysis or singular value decomposition) on these time series . Such a joint analysis would be more informative in 1) providing a global view of the comprehensive data presented in this work. 2) examining the effects and interplay of temperature and light fluence in PhyB signaling, 3) providing cross-validation of the proposed kinetic model and kinetic parameters obtained by global fitting.

Regarding the best strategy for data analysis, we appreciate this reviewer's suggestions. As powerful as singular value decomposition (SVD) and principal component analysis (PCA) doubtless are, we note that they generally apply to sizeable sets of experiments conducted at systematically varied values of one or several independent parameters, e.g., temperature. However, these conditions are not met for the experiments in Fig. 2b that underlie the mechanistic model presented in Fig. 2a. Said experiments were conducted at a single temperature and but four different light intensities. Thus, we maintain that a global analysis of these data by non-linear least-squares fitting is the method of choice here. As noted above, the mechanistical model basically extends the conventional and widely accepted model for describing phytochrome:PIF interactions.

Regarding the subsequent experiments reported in Fig. 3 and Suppl. Figs. S6-S11, we point out that they used an optical shutter to achieve a comparatively short illumination period, which contrasts with the prolonged illumination used in the experiments in Fig. 2. At least without making further assumptions, a global analysis of the different types of experiments does not appear meaningful.

All that said, we realize that the description of the data analysis approach in our initial manuscript left wanting. In the revised manuscript, we therefore include more details on the approach. Specifically, it is important to note that the entire set of experiments shown in Fig. 2b can be described remarkably well by just six global fit parameters (i.e., the rate constants k_{p}, k_{aFR}, k_{dFR}, and k_{dR}, [see Fig. 2a] and two amplitude terms). This observation lends strong credence to the underlying mechanistic model. Moreover, the fitted rate constants k_{aFR} and k_{dFR} from the global fit shown in Fig. 2b are of similar magnitude as the values determined for these rate constants in the subsequent experiments detailed in Fig. 3a-b.

Piqued by this reviewer's comment, we expanded our experiments of the PhyB:PIF interaction dynamics, originally restricted to 15°C (see Fig. 2b), to higher temperatures of 22°C and 30°C (see Suppl. Fig. S4b-c). Strikingly, the data can again be globally described by the same mechanistic

model. Consistent with the study of the temperature dependence of the association reaction (see Fig. 4a and Suppl. Fig. S6), the bimolecular association reaction (rate constant k_{aFR}) moderately sped up at 30°C compared to 15°C.

Lastly, as noted in our above response to the editor, we also include pertinent experiments on an extended *At*PhyB (1-982) construct. Its association dynamics with a PIF protein under constant illumination (see Fig. 2c, Suppl. Fig. S4d-e) conform to the same mechanistic model. Compared to the *At*PhyB PCM, the attenuation effect is even more pronounced for *At*PhyB (1-982) which is readily accounted for by the slower association kinetics (see Fig. 3f and Suppl. Fig. S11) compared to the PCM.

Taken together, the new results further strengthen the mechanistic model which can fully describe the experimental data for both the *At*PhyB PCM and *At*PhyB (1-982), and at all temperatures investigated. As arguably most prominently seen for *At*PhyB (1-982) (see Fig. 2c and Suppl. Fig. S4d-e), temperature impacts on the light-dependent PIF interaction dynamics and the attenuation. Specifically, at elevated temperatures the association reaction speeds up and the extent of attenuation diminishes.

<u>*Reviewer #2:*</u>

Authors adopted fluorescence-based monitoring system to analyze the detailed kinetics of phyBN and PIF6/PIF3 in vitro as the function of light intensity and temperature. Authors found that the interaction itself decreases as temperature increases due to more rapid increase of kd than ka, adding an additional layer of thermosensing mechanism by phyB. Surprisingly, but expectedly in a retrospect, authors found that the interaction decreases at high intensity of light due to the rapid conversion between Pr<>Pfr. The 'high intensity' used here is within a typical range of hypocotyl red fluence response curve exp (0 to 100 umole m-2 s-1), the in vivo interaction could be more complicated as, discussed in the manuscript, the current conclusion is based on phyBN and PIF in vitro. I have only two minor comments.

We appreciate the positive verdict on our manuscript.

As detailed above in our response to the editor, we expand the revised manuscript by experiments on more extended *At*PhyB constructs (see Fig. 2 and Suppl. Figs. S4 and S11).

1. It would be useful to add a Kd graph as the function of temperature in Figure 4. That will help readers to see the change of Kd over the range of temperature.

Done.

2. Though figure legends have all necessary information, it will be helpful to have what shapes/colors represent also directly on the graph.

We concur and adjust the figures throughout the manuscript and supplementary information.

Note that these revisions necessitated a renumbering of the supplementary figures. Beyond the above changes, we have slightly edited and reformatted the manuscript for accuracy and consistency. We hope that the revised manuscript is now suitable for publication in *The Plant Cell*. Thank you and the reviewers for your consideration and best regards,

Andreas Möglich

Prof. Dr. Andreas Möglich
Universität Bayreuth, Lehrstuhl für Biochemie II
Bayreuth 95447
Germany

Dear Dr. Möglich:

We have received reviews of your manuscript entitled "Plant Phytochrome Interactions Decode Light and Temperature Signals." Thank you for submitting your best work to The Plant Cell. The editorial board agrees that the work you describe is substantive, falls within the scope of the journal, and may become acceptable for publication, pending revision and potential re-review.

Your manuscript was reviewed by one new reviewer, two of the previous reviewers and the editor, the latter of whom think that this new submission has improved significantly, especially with respect to addition of a truncated dimeric phyB construct and some full-length phyB data. All of us agree that the proposed model is supported by your data and predicts the observed optogenetic signaling strength in mammalian cells and in a heterologous plant system. As such, this manuscript will be of particular interest to the optogenetic community. That said, we urge the authors to address all three reviewer's concerns (and those of this editor below) most likely requiring a revision of Discussion and inclusion of additional references. We do not require that addition data requested by Reviewer 1 is needed unless it is already available. As suggested by Reviewers 2 and 3, we advise you to provide a more nuanced (or downplayed) discussion of the relevance to phyB function in planta where there are many other phyB interactors to contend with. The issue of structural and spectral heterogeneity of both Pr and Pfr forms rasied by the reviewing editor (see below) also warrants acknowledgement in your revision.

Please contact us if there are ambiguous comments or if you wish to discuss the revision.

Given the nature of the comments, we are offering you 60 days from when we have issued this decision to complete the revision. If a revision is not returned within this time frame, and if you have not been granted an extension, we will withdraw the manuscript, which will leave you free to submit the work elsewhere. If you need an extension, we encourage you to contact us at any point before the 60 days have passed.

When you are ready to submit the revised version, please upload a highlighted copy that indicates all changes made in response to the editor and reviewer recommendations. Include an itemized list of all changes made in response to each of the reviewer's suggestions in the "Response to Reviewers" section; please note that reviewers do not have access to your cover letter, nor was this decision letter shared with them.

Thank you for the privilege of reviewing your work. We look forward to receiving your revised manuscript.

On behalf of the editorial board,

J. Clark Lagarias, Board of Reviewing Editors
George Coupland, Senior Editor
Blake Meyers, Editor-in-Chief

The Plant Cell

Please note the following:

-The Plant Cell now requires authors to complete and submit an author revisions checklist upon submission of a revised manuscript. The aim of the checklist is to aid authors in preparing a high-quality manuscript, facilitate the review and assessment of revised manuscripts, and help to ensure that journal standards are maintained across the board. If your manuscript is accepted, the completed checklist will be published as supplemental material attached to the article online. Please download a copy of the checklist (pdf fillable form) at this link, for submission with your revised manuscript: https://tpc.msubmit.net/html/Author_Revisions_Checklist.pdf.

-Supplemental materials should be restricted to large datasets and tables, presentation of replicates, and validation of reagents, methods, or genotypes. Any data that are used to support the major claims must be in the main manuscript. Supplemental figure legends must indicate what figure in the main manuscript is supported by the supplemental data presented. Please justify how each of the supplemental figures meet the criteria.

-Sampling methods and nature of "biological replicates" should be described precisely (i.e. different plants, parts of plants, pooled tissue, independent pools of tissue, sampled at different times, etc), along with a clear description of and rationale for any statistical analyses conducted. The reader should know exactly what was sampled; what forms the basis of the calculation of any means and statistical parameters reported. This is also necessary to ensure that

proper statistical analysis was conducted.

-Want to add this revision deadline to your calendar? Click below!

****
Reviewer Comments:

Reviewer #1 (Comments for the Author): The revised manuscript is much improved and has addressed my concerns. Clearly, this work is all about kinetic measurements: mScarlet-I fluorescence is used to probe the AtPhyB:PIF interaction dynamics under varying light intensity and/or temperature while the AtPhyB fluorescence is utilized to follow the Pr/Pfr photoconversion kinetics (Fig. S3).

I have one concern, however. Given that AtPhyB can adopt either the Pr (fluorescent) and Pfr (less or non-fluorescent) states, it is unclear what [AtPhyB] actually represents in Fig. 3, and Figs. S6, S8, S9. In this regard, there seems a missing puzzle piece - time series of absorption spectra that would go with these kinetic traces. Such spectral series (if coupled with joint SVD analysis) would help inform 1) the population change of [AtPhyB:Pr] and [AtPhyB:Pfr] as function of temperature or light intensity; 2) correlation between the Pfr/Pr population and "fraction of PIF in PhyB complex" (Fig. 6) or traces of mScarlet fluorescence (Fig. 2b,c; Fig. S2), or 3) presence of [AtPhyB:Pr/Pfr] (if any) and involvement of such asymmetric dimer in forming PhyB:PIF complex. Perhaps, it is unrealistic to address this missing link at this point if the authors do not already have such spectral data in hand. I am curious whether the distinction between [AtPhyB:Pr] and [AtPhyB:Pfr] in data fitting/analysis would make any difference in the major conclusions of this work. Please clarify.

Reviewer #2 (Comments for the Author): Authors have addressed the most of my concerns in the revised manuscript. One discussion point I'm curious to hear is as follows:

Many reported experiments indicate that hypocotyl length decreases continuously with the increase of red light fluence rates (typical experimental setting: from 1 µmole/m²/sec to 100 µmole/m²/sec). However, according to Figures 2d, 2e, and 2f, the degree of PIF inhibition by phyB peaks at 1 to 10 µmole/m²/s and decreases with further increases in red light fluence rates. Although authors have not shown it, it is likely that authors would observe a similar decrease for phyB and other phy-interacting proteins such as COP1/SPA. Similarly, hypocotyl length increases as temperature rises beyond 299.15K, at least up to 302.15K. However, dissociation constants for PIF3 peak at 299.15K but decrease at 303.15K (Figure 4C). I wonder if the authors could discuss their thoughts on this issue in the Discussion section, starting with "Does it matter?"

Reviewer #3 (Comments for the Author):

A critical signaling mechanism of the photoreceptor and thermosensor phytochrome B (phyB) is through the light-inducible interaction between the N-terminal photosensory module (PSM) of phyB and the active-phyB-binding motif (APB) of PIFs. This interaction is vital to phyB-mediated regulation of both PIF stability and activity. Based on theory, increases in red light intensity - which increases the fraction of the Pfr active phyB - would be expected to enhance the PSM-APB interaction, whereas high temperatures - which is expected to decrease the fraction of Pfr via an enhanced thermal reversion - should reduce the PSM-APB interaction. This manuscript investigated how light intensity and temperature changes modulate the PSM-APB interaction. The authors devised a fluorescence quenching method to assess the association and dissociation kinetics of the PSM-APB interactions, using the APBs of PIF3 and PIF6 as models. Surprisingly, the authors showed that high-light intensities attenuate the PSM-APB interaction due to significantly enhanced dissociation. Temperature increases also reduce the PSM-APB interactions, as expected. However, interestingly, temperature increases affected the binding of PSM to PIF3 and PIF6 to different extents, indicating that temperature changes affect the PSM-APB interaction differently among different PIFs. Together, this study provides novel insights into the light and temperature regulation of the PSM-APB interaction, which is important to understanding the PSM-APB's function in phyB signaling and optogenetics applications.

As computational modeling is beyond my expertise, my comments would mainly focus on interpreting the findings in the context of phyB signaling, from the perspective of a non-computational plant molecular biologist. Overall, I highly appreciate the detailed characterization of the PSM-APB interaction and found the high-light-induced attenuation of the PSM-APB interaction extremely intriguing. Although the PSM-APB interaction alone might not be sufficient to explain entirely how phyB senses light intensity and temperature, the new findings would be of great interest to the photobiology community to assess the functional role of the PSM-APB in regulating the PIF family of transcription factors. My only suggestion, in agreement with the other reviewers, would be to tune down the interpretation of the findings in phyB signaling, and particularly please reconsider the main scientific premise for the study put forward in both the introduction and Discussion sections.

- Line 87: "Specifically, in both AtPhyA and AtPhyB, the isolated PCMs and an N-terminal extension (NTE) suffice for mediating light-dependent interactions with phytochrome interesting factors (PIF).

This statement-the main scientific premise on which the study was based-is not entirely correct. The prototypic PIF3 interacts with both PSM and OPM of phyB. PIF3 was identified via a yeast two-hybrid screen using the OPM as the bait [Ni et al. (1998) Cell 85:657-67; Ni et al. (1999) Nature 400:781-4). It was recently shown that the PSM alone represses the transactivation activity of PIF3 (likely other PIFs also) and is insufficient for triggering light-dependent PIF3 degradation, which requires interaction with the OPM [Park et al. (2012) Plant J 72:537-46; Park et al. Plant Cell 20:1277-92; Qiu et al. (2017) Nat Commun 10:140; Yoo et al. (2021) Nat Commun 12:5614]. Based on the current literature, changes in the PSM-PIF interaction alone cannot explain the entire function of phyB in controlling both PIF degradation and stability, let alone phyB's role in forming the phyB-containing nuclear bodies mediated by the OPM. Also, the responses to light intensity and high temperatures in Arabidopsis are mediated primarily by PIF4, PIF5, and PIF7. Discussing whether the temperature and high-light effects could be extrapolated to those relevant PIFs would be helpful.

Reviewing Editor comments:

To me - what is different between low and high fluence rate photoequilibria - is the number of forward and reverse cycles experienced by individual phyB proteins. In other words, the amount of cycled Pr and cycled Pfr is larger under higher fluence rate than lower fluence rate equilibria. Should there be an accumulation of photoinactive 'off pathway' intermediates which are similar or overlapping with the spectra of photoactive Pr-dark and initial Pfr populations, this might provide an alternative explanation for their data, i.e. an increased heterogeneity hypothesis. In this regard, we have reported that Cph1-Pr is comprised of at least three ground state components (Kirpich et al, 2018 J Phys Chem Lett 9(12): 3454-3462), and presumably the same holds for plant phyB. If the population of non-photoactive Pr components changes upon cycling then one would see reduced PIF binding. What is particularly interesting is the observation that the same photoequilibrium is achieved after turning off the red light at all fluence rates; this suggests that 'off-pathway' intermediate(s) may be accumulating in both directions that can decay in darkness to restore the same final Pr/Pfr ratio.

Dear Dr. Coupland, dear Dr. Meyers, dear Clark,

we thank you and the reviewers again for the careful review of our manuscript and the valuable remarks. In response to the reviewers' comments, we have conducted new experiments. We enclose a revised version of our manuscript which addresses these comments as detailed below (original comments in italics, responses in blue):

Reviewer #1

The revised manuscript is much improved and has addressed my concerns. Clearly, this work is all about kinetic measurements: mScarlet-I fluorescence is used to probe the AtPhyB:PIF interaction dynamics under varying light intensity and/or temperature while the AtPhyB fluorescence is utilized to follow the Pr/Pfr photoconversion kinetics (Fig. S3).

I have one concern, however. Given that AtPhyB can adopt either the Pr (fluorescent) and Pfr (less or non-fluorescent) states, it is unclear what [AtPhyB] actually represents in Fig. 3, and Figs. S6, S8, S9.

The *x* axes in Figs. 3, S7, S9, S10, and S11 denote the total [*At*PhyB] concentration, i.e., the sum of [*At*PhyB:Pr] plus [*At*PhyB:Pfr]. (Note that the numbering of the Suppl. Figs. slightly changed during revision.) The observable rate constants k_o were evaluated as a function of the total [*At*PhyB] concentration. To account for the fractional Pfr population upon red-light exposure (ca. 73% for the *At*PhyB PCM, as determined by absorbance spectroscopy, see Fig. 1b), we corrected the resultant bimolecular association rate constants under red light, k_{aFR}, by a factor of 0.73.

To address this comment, we expand the corresponding figure legends and methods section to convey the above aspects. Likewise, we add related descriptions to the legends of Suppl. Figs. S2 (binding equilibrium *At*PhyB and P6A) and S12 (association dynamics *At*PhyB982 and P6A at different temperatures).

In this regard, there seems a missing puzzle piece - time series of absorption spectra that would go with these kinetic traces. Such spectral series (if coupled with joint SVD analysis) would help inform 1) the population change of [AtPhyB:Pr] and [AtPhyB:Pfr] as function of temperature or light intensity; 2) correlation between the Pfr/Pr population and "fraction of PIF in PhyB complex" (Fig. 6) or traces of mScarlet fluorescence (Fig. 2b,c; Fig. S2), or 3) presence of [AtPhyB:Pr/Pfr] (if any) and involvement of such asymmetric dimer in forming PhyB:PIF complex. Perhaps, it is unrealistic to address this missing link at this point if the authors do not already have such spectral data in hand. I am curious whether the distinction between [AtPhyB:Pr] and [AtPhyB:Pfr] in data fitting/analysis would make any difference in the major conclusions of this work. Please clarify.

We agree with the reviewer that time-resolved absorbance can provide additional insight into the Pr/Pfr interconversion kinetics and a possible occurrence of mixed Pr:Pfr heterodimers. Due to instrumental limitations, we are unable to record entire absorbance spectra at the required time resolution of at least 100 ms or faster. Rather, we resort to time-resolved absorbance measurements at a single wavelength of 715 nm to track the rise of the Pfr signal upon exposure to red light of varying intensities, now included as the new Suppl. Fig. S3.

Red light triggered an increase in the Pfr-state absorbance signal of the *At*PhyB PCM (Suppl. Fig. S3a) with kinetics matching those originally determined for the same light intensities by monitoring the drop of the Pr-state fluorescence (see Suppl. Fig. S4a-b). These data argue that the fluorescence measurements of the *At*PhyB PCM Pr state give an accurate account of the Pr→Pfr photoconversion kinetics (see the second part of the reviewer's point 2). Put another way, the Pr-state fluorescence signal is correlated with the Pr:Pfr distribution. As noted in the manuscript,

tracking the Pr→Pfr photoconversion by the Pr-state fluorescence has the advantage that the same instrument can be used as for the subsequent analyses of the PIF interaction dynamics.

Absorbance measurements (Suppl. Fig. S3b, d) showed that the Pfr:Pr ratio after saturating red-light illumination was around 0.73:0.27 for the *At*PhyB PCM, essentially irrespective of the applied light intensity (1, 10, 30, or 69 mW cm^{-2}) and temperature (15, 18, 22, 26, and 30°C). By contrast, for the extended *At*PhyB982 construct the Pfr:Pr ratio at photostationary state slightly increased from its value of 0.55:0.45 at 15°C and 1 mW cm^{-2} irradiation intensity to around 0.65:0.35 when increasing the temperature or light intensity (Suppl. Fig. S3c, e). Although the origin of this dependence is unclear at present, we duly consider these effects in the data evaluation. Collectively, these data address point 1) raised by the reviewer in that they reveal no significant changes in the Pr/Pfr proportions with light intensity or temperature for the *At*PhyB PCM, and a moderate influence for *At*PhyB982.

As for point 3), we consider in the following the potential occurrence and impact of Pr:Pfr heterodimers for *At*PhyB982. (By contrast, the *At*PhyB PCM is monomeric at the concentrations used, and hence no heterodimers form.) Although the lack of time-resolved absorbance spectra precludes an SVD analysis, it is worth noting that the red-light-induced Pr-state fluorescence change of *At*PhyB982 follows a single-exponential time-course. This suggests that the two subunits within the dimeric *At*PhyB982 convert between Pr and Pfr largely independently of each other. (Notably, positive or negative cooperativity between the two subunits would cause deviations from single-exponential behavior.) As a direct consequence, we expect Pr:Pfr heterodimers of *At*PhyB982 to be populated under red light. However, based on our data, we cannot reliably assess whether the Pr:Pfr heterodimer differs in its PIF affinity from that of the Pfr:Pfr homodimer.

Against this backdrop, we use the simple model to analyze the PIF association kinetics of *At*PhyB982, as we did in the case of the monomeric *At*PhyB PCM. One should note that doing so makes the implicit assumptions that i. the two subunits interconvert between Pr and Pfr independently of each other; and ii. each subunit binds PIF when in its Pfr state, irrespective of the state of the partner subunit. As the simple model adequately accounts for the experimental data, we deem more complex models unwarranted at this point in time. To address this comment, we expand the description of the analysis for *At*PhyB982.

Finally, we address the ‘correlation between the Pfr/Pr population and "fraction of PIF in PhyB complex" (Fig. 6)’ (see first part of the reviewer’s point 2). Owing to the considerably higher PIF affinity of the Pfr state vs. the Pr state, essentially all *At*PhyB molecules within PIF complexes dwell in their Pfr state. We clarify this aspect by expanding the legend to Fig. 6.

Reviewer #2

Authors have addressed the most of my concerns in the revised manuscript. One discussion point I'm curious to hear is as follows:

Many reported experiments indicate that hypocotyl length decreases continuously with the increase of red light fluence rates (typical experimental setting: from 1 µmole/m²/sec to 100 µmole/m²/sec). However, according to Figures 2d, 2e, and 2f, the degree of PIF inhibition by phyB peaks at 1 to 10 µmole/m²/s and decreases with further increases in red light fluence rates. Although authors have not shown it, it is likely that authors would observe a similar decrease for phyB and other phy-interacting proteins such as COP1/SPA. Similarly, hypocotyl length increases

as temperature rises beyond 299.15K, at least up to 302.15K. However, dissociation constants for PIF3 peak at 299.15K but decrease at 303.15K (Figure 4C). I wonder if the authors could discuss their thoughts on this issue in the Discussion section, starting with "Does it matter?"

We welcome this comment and concur that similar principal effects should be expected for other interactors such as COP1/SPA, too. The precise magnitude of these effects and the light-intensity regime in which they occur depend on the interaction parameters which await in-depth elucidation.

More generally and also noted by the editor, the situation *in planta* is more complex, not least owing to the presence of multiple other cellular constituents and subcellular architecture. Multiple factors, including other interactors, plant hormones, and energy metabolism, jointly inform and govern adaptive responses to light and temperature cues. We therefore do not expect a full correspondence between our present data and physiological responses at the quantitative level. Rather, we maintain that the fundamental light and temperature effects uncovered at present underlie cellular events and contribute to the physiological responses inside plants.

Regarding the PIF3 affinity data in Fig. 4c, we note that the precision of this evaluation is limited, and the parameter confidence intervals are hence sizeable. Although the reviewer is correct in noting a slight apparent affinity increase for both PIF3 and P3A at 303 K vs. 299 K, the values for the dissociation constants are not significantly different at these two temperatures considering the parameter confidence. We thus cannot reliably say whether there genuinely is an affinity increase for PIF3 once going beyond a certain temperature threshold.

To address this reviewer's comments, we add a paragraph to the Discussion section that considers expected effects for different PIFs and other interactors (also see our response to reviewer #3). Moreover, to better convey our data, we update Fig. 4c and now include confidence intervals for the determined dissociation constants.

Reviewer #3

A critical signaling mechanism of the photoreceptor and thermosensor phytochrome B (phyB) is through the light-inducible interaction between the N-terminal photosensory module (PSM) of phyB and the active-phyB-binding motif (APB) of PIFs. This interaction is vital to phyB-mediated regulation of both PIF stability and activity. Based on theory, increases in red light intensity - which increases the fraction of the Pfr active phyB - would be expected to enhance the PSM-APB interaction, whereas high temperatures - which is expected to decrease the fraction of Pfr via an enhanced thermal reversion - should reduce the PSM-APB interaction. This manuscript investigated how light intensity and temperature changes modulate the PSM-APB interaction. The authors devised a fluorescence quenching method to assess the association and dissociation kinetics of the PSM-APB interactions, using the APBs of PIF3 and PIF6 as models. Surprisingly, the authors showed that high-light intensities attenuate the PSM-APB interaction due to significantly enhanced dissociation. Temperature increases also reduce the PSM-APB interactions, as expected. However, interestingly, temperature increases affected the binding of PSM to PIF3 and PIF6 to different extents, indicating that temperature changes affect the PSM-APB interaction differently among different PIFs. Together, this study provides novel insights into the light and temperature regulation of the PSM-APB interaction, which is important to understanding the PSM-APB's function in phyB signaling and optogenetics applications.

As computational modeling is beyond my expertise, my comments would mainly focus on interpreting the findings in the context of phyB signaling, from the perspective of a non-

computational plant molecular biologist. Overall, I highly appreciate the detailed characterization of the PSM-APB interaction and found the high-light-induced attenuation of the PSM-APB interaction extremely intriguing. Although the PSM-APB interaction alone might not be sufficient to explain entirely how phyB senses light intensity and temperature, the new findings would be of great interest to the photobiology community to assess the functional role of the PSM-APB in regulating the PIF family of transcription factors. My only suggestion, in agreement with the other reviewers, would be to tune down the interpretation of the findings in phyB signaling, and particularly please reconsider the main scientific premise for the study put forward in both the introduction and Discussion sections.

We concur and thank the reviewer for raising these points. We revise the manuscript as detailed below.

- Line 87: "Specifically, in both AtPhyA and AtPhyB, the isolated PCMs and an N-terminal extension (NTE) suffice for mediating light-dependent interactions with phytochrome interesting factors (PIF).

This statement-the main scientific premise on which the study was based-is not entirely correct. The prototypic PIF3 interacts with both PSM and OPM of phyB. PIF3 was identified via a yeast two-hybrid screen using the OPM as the bait [Ni et al. (1998) Cell 85:657-67; Ni et al. (1999) Nature 400:781-4). It was recently shown that the PSM alone represses the transactivation activity of PIF3 (likely other PIFs also) and is insufficient for triggering light-dependent PIF3 degradation, which requires interaction with the OPM [Park et al. (2012) Plant J 72:537-46; Park et al. Plant Cell 20:1277-92; Qiu et al. (2017) Nat Commun 10:140; Yoo et al. (2021) Nat Commun 12:5614]. Based on the current literature, changes in the PSM-PIF interaction alone cannot explain the entire function of phyB in controlling both PIF degradation and stability, let alone phyB's role in forming the phyB-containing nuclear bodies mediated by the OPM.

We concur and regret the sloppy wording which is prone to misinterpretation.

To address this reviewer's comment, we expand our introductory description of plant phytochrome interactions. Specifically, we stress the key role of the OPM for the interaction and include references to the above papers.

Also, the responses to light intensity and high temperatures in Arabidopsis are mediated primarily by PIF4, PIF5, and PIF7. Discussing whether the temperature and high-light effects could be extrapolated to those relevant PIFs would be helpful.

This reviewer raises an important point that we address in the Discussion section of the revised manuscript as outlined in the following.

Although we currently do not have concrete data on the light and temperature dependence of the phytochrome interaction with other interactors and PIFs besides PIF3 and PIF6, we expect the principal phenomena to also recur for PIFs 4, 5, and 7. Two fundamental considerations speak in favor of this notion:

First, the high-light attenuation uncovered presently is engrained in the phytochromes as it owes to their bidirectional, red-light-driven Pr⇄Pfr interconversion. Under strong constant light, we thus anticipate the phytochrome interaction with other PIFs and interaction partners to weaken as well. As revealed by our investigation, the magnitude of this effect and the light intensity it occurs at stand to differ across interaction partners and thus await detailed characterization.

Second, most protein:protein association reactions exhibit a negative reaction enthalpy, i.e., they are exothermic. Thus, we expect that the phytochrome interactions with other interactors and PIFs also weaken with temperature as currently evidenced for PIFs 3 and 6. Again, the precise manifestation of this effect will vary across interaction partners.

Our work illustrates that the temperature and light regimes at which these effects play out depend on the interaction parameters and the intracellular concentrations of the phytochrome and PIFs. Future efforts could thus be directed at analyzing in more detail the phytochrome interactions with additional PIFs, and how they vary with temperature and light intensity.

Reviewing Editor

To me - what is different between low and high fluence rate photoequilibria - is the number of forward and reverse cycles experienced by individual phyB proteins. In other words, the amount of cycled Pr and cycled Pfr is larger under higher fluence rate than lower fluence rate equilibria. Should there be an accumulation of photoinactive 'off pathway' intermediates which are similar or overlapping with the spectra of photoactive Pr-dark and initial Pfr populations, this might provide an alternative explanation for their data, i.e. an increased heterogeneity hypothesis. In this regard, we have reported that Cph1-Pr is comprised of at least three ground state components (Kirpich et al, 2018 J Phys Chem Lett 9(12): 3454-3462), and presumably the same holds for plant phyB. If the population of non-photoactive Pr components changes upon cycling then one would see reduced PIF binding. What is particularly interesting is the observation that the same photoequilibrium is achieved after turning off the red light at all fluence rates; this suggests that 'off-pathway' intermediate(s) may be accumulating in both directions that can decay in darkness to restore the same final Pr/Pfr ratio. .

We appreciate the insightful comment. Indeed, several studies revealed heterogeneity in both the Pr and Pfr states of bacterial, cyanobacterial, and plant phytochromes. Heterogeneity not least arises from protonation and conformational differences around the chromophore and has for instance been documented by nuclear magnetic resonance and vibrational spectroscopy. We concur with the reviewing editor that heterogeneity likely exists in *At*PhyB as well.

While the existence of heterogeneity appears firmly established, how it impacts on light-dependent signal transduction and downstream physiological responses is much less so. A key question is whether heterogenous states in *At*PhyB substantially differ in Pr⇄Pfr photoconversion on physiologically relevant timescales and in their interactions with PIF proteins. Put another way, it is unclear whether heterogeneity gives rise to 'off-pathway' intermediates that would be incapable of or heavily impaired in undergoing light-induced protein:protein interactions. Alternatively, heterogeneity may give rise to states that do not significantly differ functionally and/or rapidly equilibrate with each other, and that therefore all maintain photoconversion and PIF interaction.

Our analyses employ the latter model and effectively disregard heterogeneity. Rather, they consider Pr and Pfr as functionally homogenous states in terms of their steady-state absorbance and PIF interaction. Several arguments speak in favor of this approach:

First, absorbance spectra acquired at photostationary state after red-light illumination reveal no changes with temperature and light intensity for the *At*PhyB PCM and moderate changes for the *At*PhyB982 variant (see Suppl. Fig. S3b-e). In all cases, the absorbance spectra exhibit isosbestic points which indicates that they differ in the Pr:Pfr proportion but not in the spectral nature of these

states. Heterogeneity cannot be ruled out but if it occurs, these heterogenous Pr and Pfr substates must have very similar electronic structures of their chromophores (otherwise one would not observe isosbestic points anymore). Moreover, the absence of systematic changes in the Pr and Pfr spectra indicates that light does not substantially alter the absorbance properties of the Pfr state. Hence, the efficiency of FRET from mScarlet-I to the Pfr state, underlying the interaction measurements (see, e.g., Fig. 2b-c) should be unaffected.

Second, as noted by the reviewing editor, in the interaction studies (e.g., Fig. 2b-c) the Pr:Pfr photoequilibria reached after illumination ceases appear to be independent of the light intensity, as, e.g., indicated by the fast convergence of the fluorescence traces once light shuts off. These data suggest that 'off-pathway' intermediates, if populated in the first place, are comparatively short-lived and rapidly equilibrate with the 'on-pathway' species. Back 'on path', these *At*PhyB molecules could then enter light-induced PIF interactions. Certainly, subsequent light absorption could again convert these *At*PhyB molecules to a 'non-interacting' state and prompt PIF release. This, however, corresponds to the central premise of our model: light absorption drives the Pfr→Pr reversion and thereby actively promotes PIF dissociation.

Third, the PIF interaction experiments used an at least 50-fold molar excess of *At*PhyB (or, *At*PhyB982) over the PIF protein. Even if a certain *At*PhyB fraction were to be converted to a 'non-photoactive'/'non-interacting' state and thus temporarily incapacitated, the remaining, active fraction could pick up the slack and associate with PIF. Notably, the experiments used an *At*PhyB concentration way above the relevant K_d for the PIF interaction. Even if part of the *At*PhyB molecules is temporarily taken out of the game, enough active species remain to saturate the PIF binding.

Fourth, an additional argument can be fielded. Our current model is the arguably simplest mechanistic model that can adequately describe the experimental data. If one were to allow for heterogeneity, additional species would need to be introduced, thus making for a more complex model. Given the success of the simpler model, we feel that such more complex models are not warranted at present.

All that said, we note that we cannot rule out a contribution of Pr and Pfr heterogeneity to the observable experimental data. We therefore highly appreciate the reviewing editor's astute comment and have added a brief description of heterogeneity in phytochromes at the end of the section on 'Light Fluence Rate Modulates Kinetics and Extent of the *At*PhyB:PIF Interaction'.

Note that these revisions necessitated a renumbering of the supplementary figures; all figure references in the above text refer to the new numbering. Beyond the above changes, we have slightly edited and reformatted the manuscript for accuracy and consistency. We hope that the revised manuscript is now suitable for publication in *The Plant Cell*. Thank you and the reviewers for your consideration and best regards,

Andreas Möglich

Dear Andreas:

We are pleased to inform you that your paper entitled "Plant Phytochrome Interactions Decode Light and Temperature Signals" has been accepted for publication in The Plant Cell, pending a final editorial review by a science editor. At this stage, your manuscript will be evaluated by a science editor with respect to its presentation of scientific content, compliance with journal policies, and presentation for a broad readership. The Plant Cell has contracted with Plant Editors (planteditors.com) to provide this service to our authors, and you will soon receive additional information on this process.

Please note that each author needs to link their ORCID identifier to their account in the system before your manuscript can be published. If any authors do not have an ORCID linked to their account, they will receive a message with a link to complete this task. Please ensure that ALL of your coauthors have completed this task as soon as possible.

LICENSE INFORMATION Shortly after your paper is sent to production, you will receive an email from our publisher's author support team, **SciPris**, with information on article processing charges, as well as the types of Open Access license you would be able to purchase. The accepted version of your manuscript will not appear on our Advance Articles page until the license is selected and signed, so please look out for an email from SciPris. For more information see the Article Publication Fees section of our author guidelines: https://academic.oup.com/plcell/pages/General-Instructions

The Plant Cell and The Arabidopsis Information Resource (TAIR) are collaborating to collect functional annotation data about Arabidopsis genes from authors. This includes information about the gene's molecular function (e.g., kinase activity, ATP synthetase activity), the biological process/es it is involved in (e.g., endosperm development, threonine biosynthesis), its subcellular location (e.g., nucleus, ER), anatomical or developmental expression pattern (e.g., leaf, ovule, flower stage 10, seedling stage), or its partner in a protein-protein interaction (e.g., AT1G01010 interacts with AT1G01020).

If your paper contains results falling into one or more of these categories for Arabidopsis genes, we request that you now submit these data for inclusion in TAIR by filling in the form provided at the following URL: https://www.arabidopsis.org/doc/submit/functional_annotation/123. If you need further clarification on what types of data can be submitted please contact curator@arabidopsis.org.

Finally, we encourage your submission of artwork for the journal cover. If there is an image or illustration related to your paper that you would like to have considered, please email it in .pdf or .tif format to clowe@aspb.org. An image title, brief, 2-3 sentence description of the image, and the name of the person credited for the image are also encouraged. If your image is selected to appear on the online cover of the journal, you will be contacted with the scheduled issue date and further information.

We look forward to seeing your paper published.

On behalf of the editorial board,

J. Clark Lagarias, Board of Reviewing Editors
George Coupland, Senior Editor
Blake Meyers, Editor-in-Chief

The Plant Cell

=====
IMPORTANT REMINDER: PEER REVIEW REPORTS
=====

If you opted to publish a peer review report along with your article during the original submission process, it will be prepared by the editorial staff and publicly posted with your manuscript, inside the zip file that contains any other supplemental material. As a reminder, the peer review report is a public record of all comments from editors and reviewers, as well as your prior responses, as you received them in the decision letters for each draft of your manuscript. If you agreed to publish this report and have changed your mind, or are not sure if you selected this option, please contact the editorial office as soon as possible before signing the license agreement from our publisher.

=====
REVIEWER COMMENTS
=====

Reviewer #1 (Comments for the Author):

This revision has addressed my concerns. Overall, I think this work is an important addition to the phytochrome field. With more conformational states are captured and/or reported for the full-length structures of plant phytochromes and bacteriophytochromes (published and unpublished), I expect that many kinetic data and findings presented in this work (along with the implicit assumptions) will be scrutinized, extensively revisited and cited by the field that is still short of THE mechanistic understanding of the phytochrome action as a dimer. Therefore, this publication is timely and important. I support its publication in Plant cell.

Reviewer #2 (Comments for the Author):
The authors have carefully and thoroughly revised the manuscript. This finding will prompt researchers in the field to reconsider the overlooked nature of Pr<>Pfr in light and its consequences on interactions with other proteins.

Reviewer #4 (Comments for the Author):
The authors have addressed all my comments. I have no further questions.

Editor (Comments to Author):
This is a very nice contribution that highlights critical issues surrounding light intensity- and temperature-mediated phyB-PIF3/6 interaction relevant to optogenetic applications. The authors have done a nice job of integrating these findings with their potential relevance to phyB signaling in plants, making this a contribution that should be of broad interest to the plant biology community.

----FOR ASPB OFFICE USE ONLY (DO NOT EDIT)----
MSID: 36270
Corresponding Author: Prof. Dr. Möglich
